# The UPR Branch IRE1-*bZIP60* in Plants Plays an Essential Role in Viral Infection and Is Complementary to the Only UPR Pathway in Yeast

**DOI:** 10.1371/journal.pgen.1005164

**Published:** 2015-04-15

**Authors:** Lingrui Zhang, Hui Chen, Federica Brandizzi, Jeanmarie Verchot, Aiming Wang

**Affiliations:** 1 Southern Crop Protection and Food Research, Agriculture and Agri-Food Canada, London, Ontario, Canada; 2 Department of Energy Plant Research Laboratory and Department of Plant Biology, Michigan State University, East Lansing, Michigan, USA; 3 Department of Entomology and Plant Pathology, Oklahoma State University, Stillwater, Oklahoma, United States of America; University of Alabama at Birmingham, UNITED STATES

## Abstract

The unfolded protein response (UPR) signaling network encompasses two pathways in plants, one mediated by inositol-requiring protein-1 (IRE1)-*bZIP60* mRNA and the other by site-1/site-2 proteases (S1P/S2P)-bZIP17/bZIP28. As the major sensor of UPR in eukaryotes, IRE1, in response to endoplasmic reticulum (ER) stress, catalyzes the unconventional splicing of *HAC1* in yeast, *bZIP60* in plants and XBP1 in metazoans. Recent studies suggest that IRE1p and *HAC1* mRNA, the only UPR pathway found in yeast, evolves as a cognate system responsible for the robust UPR induction. However, the functional connectivity of IRE1 and its splicing target in multicellular eukaryotes as well as the degree of conservation of IRE1 downstream signaling effectors across eukaryotes remains to be established. Here, we report that IRE1 and its substrate *bZIP60* function as a strictly cognate enzyme-substrate pair to control viral pathogenesis in plants. Moreover, we show that the S1P/S2P-bZIP17/bZIP28 pathway, the other known branch of UPR in plants, does not play a detectable role in virus infection, demonstrating the distinct function of the IRE1-*bZIP60* pathway in plants. Furthermore, we provide evidence that *bZIP60* and *HAC1*, products of the enzyme-substrate duet, rather than IRE1, are functionally replaceable to cope with ER stress in yeast. Taken together, we conclude that the downstream signaling of the IRE1-mediated splicing is evolutionarily conserved in yeast and plants, and that the IRE1-*bZIP60* UPR pathway not only confers overlapping functions with the other UPR branch in fundamental biology but also may exert a unique role in certain biological processes such as virus-plant interactions.

## Author Summary

The unfolded protein response (UPR) is crucial to life as it regulates gene expression in response to stress in the endoplasmic reticulum (ER). There are two functionally overlapping UPR branches in plants, e.g., IRE1-*bZIP60* and S1P/S2P-bZIP17/bZIP28, but only one, IRE1p-*HAC1*, in yeast. Despite recent significant progress in understanding UPR, a functional connectivity of IRE1 and its splicing target has not been established in multicellular eukaryotes. It is unknown if a single UPR branch in plants has any unique biological functions. Given that all eukaryotes are equipped with the IRE1-mediated pathway, are IRE1 downstream signaling effectors conserved among kingdoms and at what degree? Here, we show that IRE1 and its substrate *bZIP60* function as a matched enzyme-substrate pair to mediate virus-host interactions in plants. We further provide evidence that a single UPR branch, IRE1-*bZIP60*, rather than S1P/S2P-bZIP17/bZIP28, determines viral pathogenesis, indicating that the two UPR arms may have distinct functions in plants. Finally, we demonstrate that the spliced form bZIP60 and HAC1p, rather than IRE1, are functionally replaceable to cope with abiotic stress in yeast, suggesting that the downstream signaling of the IRE1-mediated splicing is evolutionary conserved in plants and yeast. These data shed new lights into UPR in multicellular eukaryotes.

## Introduction

The accumulation of unfolded protein in the endoplasmic reticulum (ER) results in ER stress that triggers the unfolded protein response (UPR), a complex signal transduction cascade that orchestrates adaptation to ER stress or induces apoptosis if ER stress remains unmitigated [1–6]. In mammalian cells, the UPR is mediated by three classes of membrane-associated sensor transducers including inositol-requiring transmembrane kinase/endonuclease (IRE1), protein kinase RNA (PKR)-like ER kinase (PERK), and activating transcription factor 6 (ATF6) [2,3]. In contrast to animals, the UPR in yeast is controlled by only the IRE1p-mediated signaling pathway, which triggers the expression of over 5% genes mainly encoding chaperones and ER-associated protein degradation components in the genome [7]. The IRE1 lumenal domain at the N-terminus serves as a UPR sensor domain and the C-terminal cytoplasmic portion encompassing serine/threonine protein kinase and endoribonuclease domains functions as an effector domain [3]. Upon sensing ER stress, IRE1 dimerizes or oligomerizes in the plane of the ER membrane through the binding of unfolded proteins to its UPR sensor domain or the release of oligomerization-repressing chaperones, or both, allowing for *trans*-autophosphorylation of juxtaposed kinase domains [8–10]. Based on the crystal structure of the dual catalytic region of IRE1, the *trans*-autophosphorylation of the kinase domain of IRE1 is suggested to permit unfettered binding to nucleotides (nt), which in turn promotes dimerization of IRE1 to compose the active ribonuclease site, thus unmasking the dormant endoribonucleolytic activity [3,11].

The allosteric activation of IRE1 entails the sequence-specific cleavage of a single known messenger RNA encoding a basic leucine zipper (bZIP) transcription factor—ATF/CREB1 (HAC1) in yeast [12] or X-box binding protein-1 (XBP1) in metazoans [13]. The IRE1-dependent mRNA cleavage is an unconventional splicing, which occurs predominantly in the cytoplasm in a spliceosome-independent manner [14]. While the unconventional splicing mediated by IRE1 does not comply with Chambon’s rule (GU-AG rule) at the exon-intron border, it requires the existence of a pair of characteristic stem–loop structures in mRNA, which drive the projected splicing sites close to the ribonuclease catalytic sites in the cytosolic domain of IRE1 [15,16]. IRE1 catalyzes the cleavage at the conserved sites in both of the 7-nt loops of the mRNA precursors unspliced *HAC1* (*HAC1* U) and *XBP1* (*XBP1* U), excising a 252- and 26-bp intervening intron to produce the spliced form of *HAC1* (*HAC1* S) and *XBP1* (*XBP1* S), respectively [17], leading to a frame-shift and introduction of a new termination codon in both coding sequences. Owing to the frame-shift, the encoded HAC1 S and XBP1 S proteins both gain a transcriptional activation domain (AD) at their C-termini, which is necessary for the transcription of downstream genes [18–20]. In yeast, the translation of *HAC1* U mRNA is hampered due to the presence of a translational inhibitor in the intron, and relief of this repression via producing *HAC1* S is the key activating event for the yeast UPR [20]. By contrast, in metazoans both *XBP1* U and *XBP1* S are translated [13,19]. Nevertheless, XBP1 U complexes with the XBP1 S protein, which is more stable and transcriptionally active for UPR target genes, and exports it to the cytoplasm for proteasome-dependent degradation because a nuclear export signal and a degradation domain are present in the C-terminus of XBP1 U [13,19]. As a result, XBP1 S action is shut down during the later phase of ER stress, and XBP1 U is thus regarded as an inhibitor of the UPR in higher eukaryotes [13,19,21].

In plants, two UPR pathways have recently been identified, one mediated by IRE1-*bZIP60*, and the other by site-1/site-2 proteases (S1P/S2P)-bZIP17/bZIP28, which is analogous to the animal ATF6 pathway [15,22–25]. Although two genes, *IRE1A* and *IRE1B* in the genome of *Arabidopsis* (*Arabidopsis thaliana*), were found to encode IRE1 homologs a decade ago [26,27], their involvement in the plant UPR remained undetermined until most recently when *bZIP60* mRNA was identified as the RNA target of IRE1A/IRE1B for unconventional splicing [1,15,22,28–30]. *bZIP60* does not share a high sequence identity with *HAC1* and *XBP1* at both nucleotide and protein levels. However, like *HAC1* and *XBP1* mRNA, *bZIP60* mRNA can fold into an IRE1 recognition site composed of two stem loops, each containing the bases at three positions remarkably conserved from yeast to mammalians [15,22,31,32]. Although the second loop from *bZIP60* mRNA consists of 8 instead of 7 nt, the “kissing” stem loops in which the two stems are capable of base-pairing with each other are still formed to protrude the cleavage sites to the catalytic sites of IRE1, which is essential for IRE1-dependent splicing of *HAC1* and *XBP1* mRNAs [11,15,22,31,33]. In response to pathogen infection (i.e., *Pseudomonas syringae*), heat or salicylic acid stimuli, as well as ER stress agents, such as tunicamycin (Tm) and dithiothreitol (DTT), *bZIP60* mRNA is spliced to remove a 23-bp fragment in *Arabidopsis* [15,22,30]. As a result, a translational frame-shift occurs downstream of the splicing sites and eliminates a single transmembrane domain (TMD) encoded by unspliced *bZIP60* (*bZIP60* U) to produce bZIP60 S, which is an active transcription factor that up-regulates the UPR target genes, such as *BiP* (coding for lumenal binding proteins), *CAM* (calmodulin), *CRT* (calreticulin) and *PDI* (protein disulphide isomerase) [15,22,30,33]. Although the IRE1-mediated mRNA splicing apparently is a conserved strategy for the IRE1 signaling across eukaryotes [34], functional inter-kingdom equivalence of bZIP60 with HAC1 and XBP1 is yet to be demonstrated.

Analyses of three independent homozygous transfer DNA (T-DNA) insertion lines of *IRE1A* (*ire1a-2*, SALK_018112; *ire1a-3*, WiscDsLox420D09, *ire1a-4*, SAIL_1256_F04) and a knockout mutant of *IRE1B* (*ire1b-4*; SAIL_238_F07) showed that IRE1A has little effect on the *bZIP60* mRNA splicing in *Arabidopsis* seedlings in response to DTT or Tm treatment, whereas IRE1B plays a major role in the *bZIP60* mRNA processing [15,30]. However, the findings obtained in a different *ire1b* mutant (*ire1b-1*, GABI_638B07) demonstrated that the single *IRE1B* mutation does not affect the *bZIP60* splicing caused by Tm treatment, whereas stress-induced *bZIP60* splicing is eliminated in the *ire1a-2 ire1b-1* or *ire1a-3 ire1b-4* double mutant [22,30]. Thus, IRE1A and IRE1B seem functionally redundant through splicing *bZIP60* mRNA. In addition, the *ire1a-3 ire1b-4* double mutant, but not the single mutant lines, develops a short-root phenotype as a result of a disorder in cell elongation in the transition zone/elongation zone [28], whereas the mutation in their RNA target *bZIP60* does not lead to the similar short-root phenotype [35]. Therefore, the function of IRE1A and IRE1B with respect to the *bZIP60* mRNA splicing in specific biological event(s) still remains mysterious. To make it more complex, several recent studies have also demonstrated that the two arms of the UPR in plants functionally overlap in abiotic stress [24,25,32,36,37], which motivates us to ask whether the single branch of the two UPR arms has any unique functions.

Here, we report that IRE1A and IRE1B are fully functionally redundant for the production of *bZIP60* S to determine the extent of plant diseases caused by *Turnip mosaic virus* (TuMV) infection. Moreover, the IRE1-*bZIP60* mRNA pair mediating viral infection is independent of the S1P/S2P-bZIP17/bZIP28 pathway. To the best of our knowledge, this is the first report showing that a biological process in plants is regulated by a single UPR pathway, in which IRE1 and *bZIP60* mRNA function in a projected linear manner. We further show that *bZIP60* and *HAC1*, rather than IRE1, are functionally replaceable to cope with abiotic stress response in yeast. Taken together, our data unravel an evolutionarily conserved role of the IRE1-*bZIP60* pathway in regulation of abiotic and biotic stresses, shedding new lights on the complex UPR signaling pathways.

## Results

### *bZIP60* Is Spliced in Response to TuMV Infection

**Fig 1:**
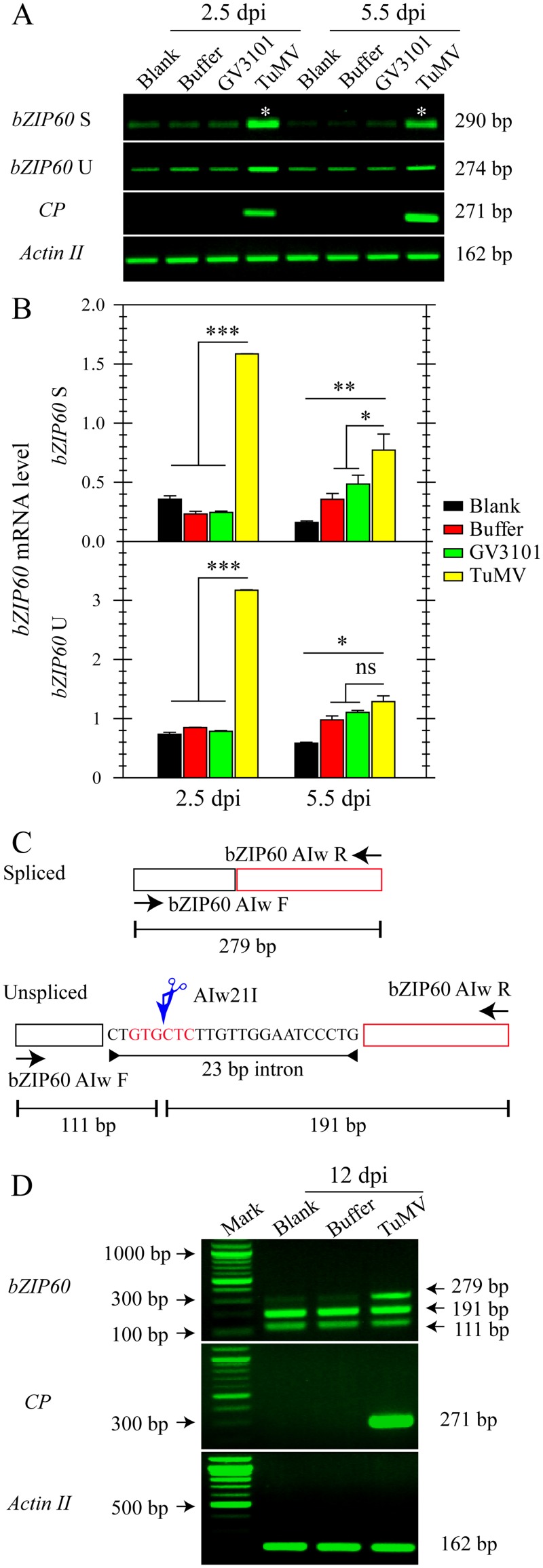
The splicing of *bZIP60* is induced in response to TuMV infection. **(A)** Semi-quantitative RT-PCR analysis of *bZIP60* U, *bZIP60* S and *coat protein* (*CP*) expression at 2.5 and 5.5 dpi in the local rosette leaves from the indicated treatments. *Actin II* was analyzed as a loading control. Note that we loaded 4 times the amount of control for *bZIP60* S since its level is much lower *in vivo* less than *bZIP60* U [22]. PCR product sizes are indicated at right. Note that colony diagnostic test and sequencing showed that the two bands marked with asterisks do not contain the 23-bp intron (see S1 Fig). **(B)** The mRNA level of *bZIP60* S and *bZIP60* U was determined by qRT-PCR. *Actin II* was used as an internal control for qRT-PCR. Data represent means with SD of three biological replicates. * *P*<0.05, ** *P*<0.01, *** *P*<0.001, unpaired two-tailed Student’s test. ns, non-significant. **(C)** and **(D)** Flanking assay for detecting *bZIP60* S in systemically infected leaves. **(C)** Schematic representation of the flanking approach used in this study [30]. The primer sets flanking the 23-bp intron amplify the *bZIP60* S (top) and *bZIP60* U (bottom). Due to the absence of Alw21I restriction site in spliced fragments, only un-spliced fragments are digested by Alw21I restriction enzyme, thus producing two smaller fragments. **(D)** A unique fragment corresponding to spliced forms of *bZIP60* could only be detected under TuMV attack, whereas the other two smaller fragments corresponding to the Alw21I digested products were detectable in all groups. *Actin II* and *CP* were analyzed as a control and an indicator of TuMV infection. PCR product sizes are indicated at right.

Recent studies have shown that viral infection may trigger UPR in plants [38,39]. To investigate how the UPR is implicated in viral infection, we first examined whether the IRE1-*bZIP60* pathway is activated under viral attack. A pair of primers that specifically captures *bZIP60* S transcripts was designed to detect *bZIP60* splicing by RT-PCR (S1A Fig, S1 Table). In *Arabidopsis* local leaves inoculated with TuMV-GFP, a recombinant TuMV tagged by green fluorescence protein (GFP), the *bZIP60* S greatly accumulated at 2.5 and 5.5 days post-infection (dpi), compared with the controls (Fig 1A). The absence of the 23-bp intron in the amplified products was confirmed by colony diagnostic testing and DNA sequencing (S1B and S1C Fig). Quantitative analyses demonstrated that the level of *bZIP60* S in TuMV-infected plants was significantly higher than that in controls at both time points (Fig 1B). Although *bZIP60* U was also significantly increased at 2.5 dpi in response to TuMV challenge, it returned to the level not significantly different from that in the buffer or agrobacterium-inoculated controls at 5.5 dpi (Fig 1A and 1B). To investigate if the IRE1-*bZIP60* pathway is also activated in systemically infected leave, the *bZIP60* S was monitored following an approach recently developed by Moreno *et al*. [30] (Fig 1C). Result demonstrated that a unique cDNA fragment corresponding to the spliced form of *bZIP60* mRNA was clearly evident in the newly emerging leaves of *Arabidopsis* seedlings inoculated with TuMV, but barely detectable in the corresponding leaves of control plants rubbed without or with buffer (Fig 1C and 1D). Taken together, these data indicated that TuMV infection induces *bZIP60* mRNA splicing in both locally and systemically infected levels.

Since the splicing of *bZIP60* mRNA is initiated by TuMV infection, we explored its downstream signaling by determining the expression of ER stress marker genes such as *BiP*, *CRT*, and *PDI*, which have been shown as the targets of bZIP60 [39]. In local inoculation leaves, the expression of *BiP3*, *BiP1/2* and *PDI* was remarkably increased at 3, 6 and 9 dpi in response to TuMV infection, compared to the controls (S2B and S2C, S2E Fig). The expression of the ER marker gene *CRT* was also increased at 3 dpi under TuMV attack, even though it was barely detectable during the late phases of infection (S2E Fig). Overall, the expression of these UPR marker genes was also up-regulated at 6 and 9 dpi in systemically infected leaves (S2D and S2F Fig). These results suggested that the bZIP60 UPR signaling pathway is indeed activated in both local and systemic leaves in response to TuMV infection.

### The Viral Membrane Protein 6K2 Is an Inducer of *bZIP60* Splicing

**Fig 2:**
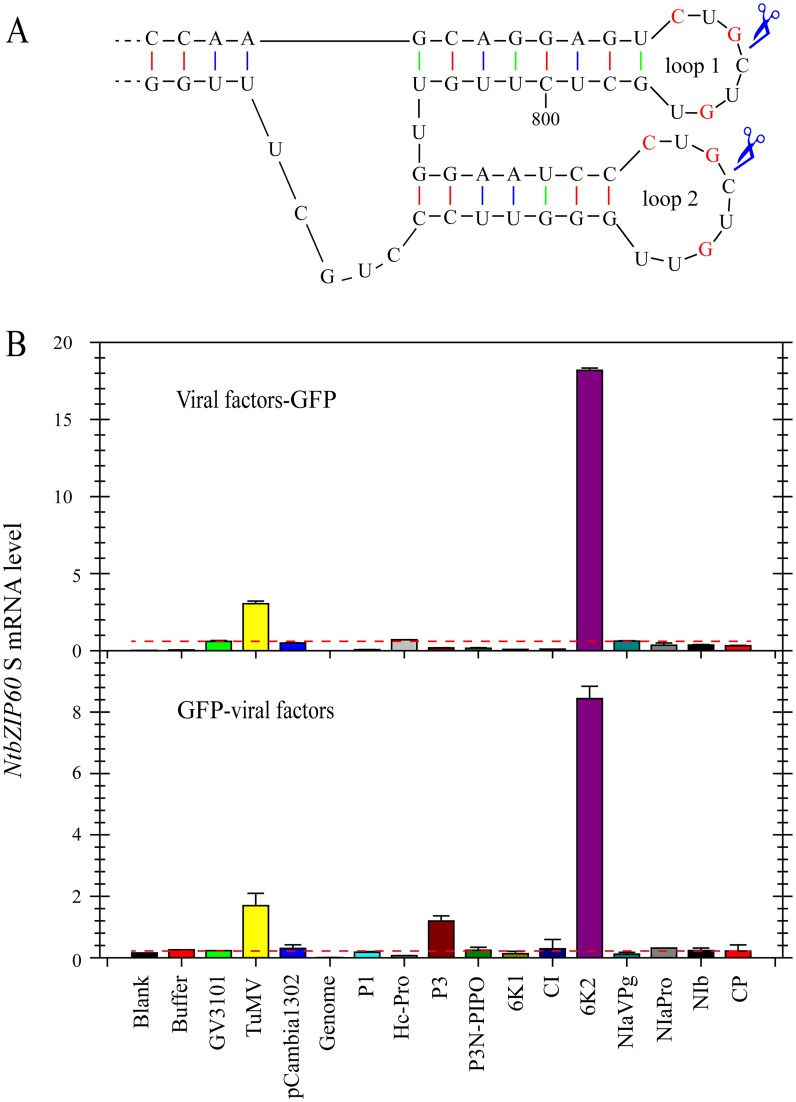
TuMV 6K2 is an inducer of *bZIP60* splicing. **(A)** Twin hairpin loop structure in *NtbZIP60* mRNA, which is the magnification of red boxed area in S4 Fig. Each of the two loops contains three conserved bases (red). Scissors indicate predicted cleavage sites. **(B)** qRT-PCR analysis of *NtbZIP60* S level in *N*. *benthamiana*. 11 TuMV factors fused with a C-terminal (top) or N-terminal (bottom) GFP were transiently expressed alone via agroinflitration. At 2.5 dpi, the transient expression of each construct was verified under confocal by observing GFP (see S3 Fig), and RNA was extracted from the agroinflitrated leaves. The RNAs from un-infiltrated leaves (Blank) and the leaves infiltrated with buffer, GV3101, GV3101 containing TuMV infectious clone and pCabmibia1302 were used as controls. Genomic DNA was also analyzed as a control by qRT-PCR. *18S RNA* was used as an internal control. Note that the lines drawn according to the value of *NtbZIP60* S caused by GV3101 were used to highlight that only TuMV 6K2 in both types of constructs could strongly induce *NtbZIP60* splicing. The Data represent means with SD of three biological replicates.

To determine which TuMV protein(s) is responsible for inducing the splicing of *bZIP60*, transient expression assays were performed in *Nicotiana benthamiana* (*N*. *benthamiana*). Like other potyviruses, TuMV encodes a total of 11 mature proteins, i.e., P1, HcPro, P3, P3N-PIPO, 6K1, CI, 6K2, NIaVPg, NIaPro, NIb and CP [40]. Plant expression vectors encoding each of the 11 viral proteins fused with GFP (viral factor-GFP fusion) or their reciprocal form (GFP-viral factor fusion) were created and transiently expressed in *N*. *benthamiana* via agroinfiltration (S3 Fig). Here, the sequence of *NtbZIP60* was used for the splicing assay in *N*. *benthamiana* [39]. *NtbZIP60* is homologous to *Arabidopsis bZIP60*. *NtbZIP60* mRNA was predicted to fold a conserved twin hairpin loop based on the RNA structure prediction program M-Fold [41] and the sequence in the double hairpin loop region is nearly identical in at least 20 *bZIP60* homologues in plants (Figs 2A, S4 and S5), suggesting that *NtbZIP60* is a potential target of unconventional splicing in *N*. *benthamiana*. Using a primer set specific for spliced *NtbZIP60* (*NtbZIP60* S), quantitative RT-PCR (qRT-PCR) revealed that TuMV infection triggered *NtbZIP60* splicing in *N*. *benthamiana* as expected (Fig 2B, S1 Table). Of the 11 viral factors, the viral membrane protein 6K2 (either in the form of 6K2-GFP or GFP-6K2) strongly induced the accumulation of *NtbZIP60* S (Fig 2B). These data suggested that the viral membrane protein 6K2 is an inducer of *bZIP60* splicing.

### Viral Accumulation Is Reduced in *bzip60-2* Mutant but Not in *bzip60-1* Mutant

**Fig 3:**
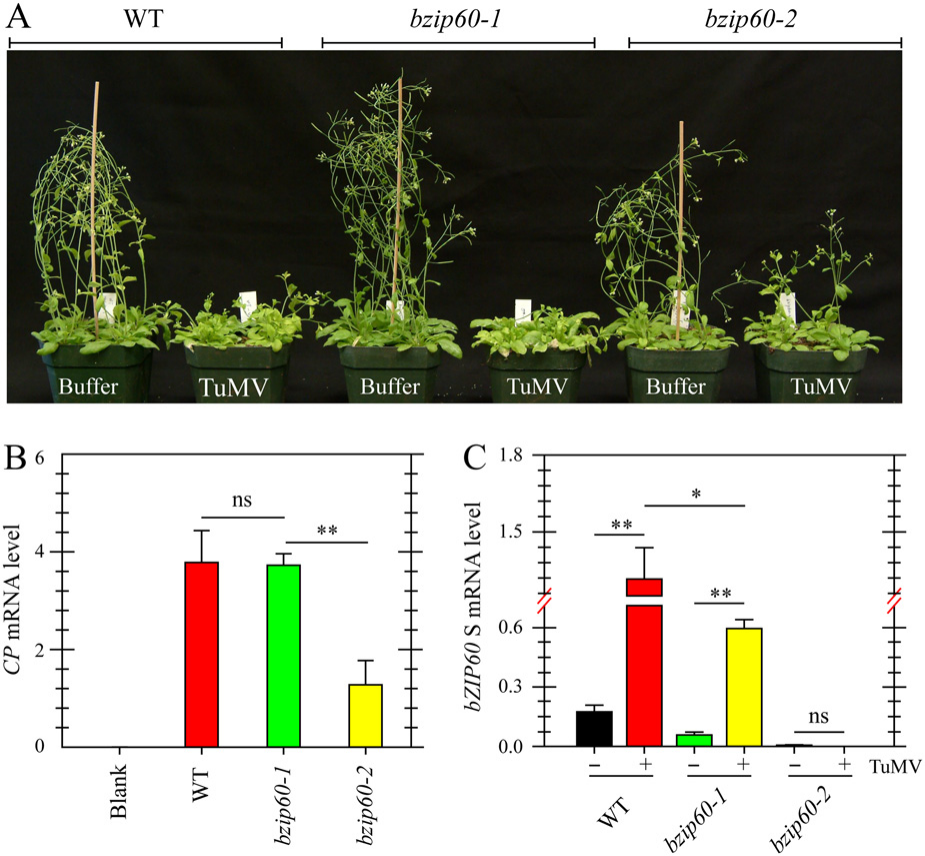
Viral pathogenesis is alleviated in the *bzip60-2* mutant. **(A)** Phenotypes of the wild type, *bzip60-1* and *bzip60-2* mutants at 18 dpi after inoculation with buffer or TuMV. Note that the *bzip60-2* mutant, but not the *bzip60-1* mutant, alleviated TuMV symptoms, compared to the wild type. **(B)** and **(C)** qRT-PCR analysis of TuMV *CP* **(B)** and *bZIP60* S **(C)** in the wild type, *bzip60-1* and *bzip60-2* mutants. At 18 dpi after inoculation with buffer or TuMV, RNA was extracted from the systemically infected leaves, and qRT-PCR was carried out. For detecting *CP* level, the RNAs were collected from the wild type, *bzip60-1* and *bzip60-2* mutants under buffer treatment as a blank control. *Actin II* was used as an internal control. Data represent means with SD of three biological replicates. * *P*<0.05, ** *P*<0.01, unpaired two-tailed Student’s test. ns, non-significant.

Since TuMV and TuMV 6K2 induced the splicing of *bZIP60* mRNA in plants, the role of *bZIP60* S in viral pathogenesis was examined. Here, two independent T-DNA insertion mutants in *bZIP60* (*bzip60-1*, SALK_050203; *bzip60-2*, SAIL_283_B03) were used [15,42]. Seedlings of the wild type *Arabidopsis* and the *bzip60-1* mutant inoculated with TuMV developed typical TuMV symptoms, including mosaic, leaf yellowing and stunted growth, compared to the mock-inoculated control (Fig 3A). However, the viral symptoms in the *bzip60-2* mutant were much milder than the wild type and the *bzip60-1* mutant, although the stature of TuMV-infected *bzip60-2* mutant was smaller than that of mock-inoculated plants (Fig 3A). Quantitative analyses indicated that TuMV accumulated to similar levels in the wild type and *bzip60-1* plants (Fig 3B), but in the *bzip60-2* mutant, the level of the virus was much lower than that in the wild type and *bzip60-1* mutant (Fig 3B, *P*<0.01). These data clearly indicated that viral pathogenesis is alleviated in the *bzip60-2* mutant. Intriguingly, in comparison with that in mock-inoculated plants, *bZIP60* S transcripts were significantly accumulated in the systemically infected leaves of both wild type and *bzip60-1* plants inoculated with TuMV (Fig 3C, *P*<0.01), and the *bZIP60* S level in the wild type was significantly higher than that in the *bzip60-1* mutant (Fig 3C, *P*<0.05). In contrast, no *bZIP60* S was detectable in the *bzip60-2* mutant either under or without TuMV attack (Fig 3C). Taken together, these data suggested an association of TuMV accumulation and viral pathogenesis with the *bZIP60* S.

### *bzip60-1* and *bzip60-2* Are Non-RNA Null Mutants and Transcribe Incomplete bZIP60 ORF

**Fig 4:**
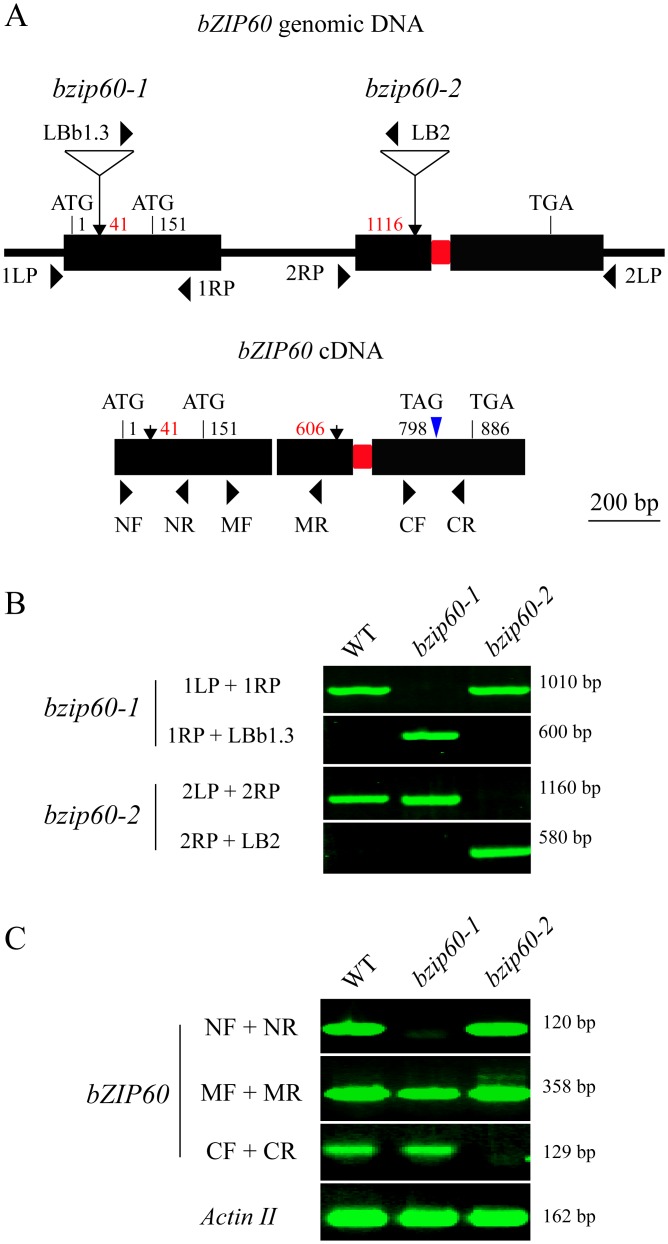
Molecular characterization of the *bzip60* mutants. (**A**) Schematic representation of *bZIP60* genomic DNA (top) and cDNA (bottom) structure with intron and exons. The positions of the T-DNA insertion in the two *bzip60* mutants are indicated by arrows in genomic DNA, and corresponding positions in the cDNA structure are also shown. The start and stop codons of two in-frame ORFs are labeled (ATG1 for *bZIP60* and ATG151 for *bZIP60ΔN*). Note that a 23-bp sequence within the second exon to be spliced through unconventional splicing is represented by a red rectangle. The new termination codon (TAG798) due to a frame-shift caused by splicing is indicated by a blue triangle. **(B)** and **(C)** PCR with genomic DNA **(B)** and cDNA **(C)** from the wild type and the *bzip60* mutants to test the homozygosis and the presence of non-full length *bZIP60* mRNA fragments in the mutant lines. PCR product sizes are indicated at right. The primer locations are shown in **(A)**, and primer sequences are given in S1 Table. In **(C)**, *Actin II* served as a control.

The finding that the *bzip60-1* and *bzip60-2* mutants develop contrasting viral symptoms promoted us to re-examine the molecular characterization of the two mutants. By sequencing the T-DNA flanking regions, we mapped the T-DNA insertion at position 41 and 1116 nt downstream from the translation initiation codon (ATG1) of *bZIP60* genomic DNA in *bzip60-1* and *bzip60-2* (Figs 4A and S6A and S6B), respectively, indicating that the genomic DNA structure in the two mutants was disrupted. Genomic PCR analyses indicated that both *bzip60* mutants are homozygous mutant lines (Fig 4A and 4B). RT-PCR with three specific pairs of primers (NF + NR, MF + MR, and CF + CR) was carried out to further determine whether the *bzip60-1* and *bzip60-2* mutants represent RNA-null mutants (Fig 4A, S1 Table). Although no *bZIP60* amplicon was detectable in the *bzip60-1* mutant using the primers flanking the insertion site (NF + NR), *bZIP60* transcripts were present in the *bzip60-1* mutant using the downstream primer sets (MF + MR and CF + CR) (Fig 4A and 4C). In contrast, *bZIP60* amplicon could not be detected in the *bzip60-2* mutant by the downstream primer set (CF + CR), while other two amplicons could be seen using the upstream primer sets (NF + NR and MF + MR) (Fig 4A and 4C). These results suggested that neither *bzip60-1* nor *bzip60-2* is an RNA-null mutant, although both of them indeed do not express full-length *bZIP60* transcripts.

Analyses of the *bZIP60* cDNA sequence revealed that there is a potential in-frame start codon at position 151 relative to the start codon of the previously identified *bZIP60* ORF (Figs 4A, S6A and S7A). Thus, the *bzip60-1* mutant might produce a shorter version of *bZIP60* ORF (*bZIP60ΔN*). To prove this assumption, 5′ rapid amplification of cDNA ends (5′ RACE) was used to determine the 5′ end of *bZIP60* mRNA in this mutant. DNA sequencing results indicated that all selected cDNAs from the *bzip60-1* mutant after different treatments contain the intact ORF from the alternative start codon, although the 5′ end of *bZIP60ΔN* varied in length (S7A Fig). In addition, analyses of the *bzip60-2* mutant revealed that T-DNA insertion in the *bZIP60* genome disrupts the 3′ end of *bZIP60* mRNA, which causes the premature termination of *bZIP60* translation at nt 52 of the T-DNA sequence (S7B Fig, Stop ΔC). Taken together, these findings showed that the *bzip60-2* mutant transcribes incomplete *bZIP60* mRNA (*bZIP60ΔC*) coding for a truncated bZIP60 without the C-terminus, whereas the *bzip60-1* mutant produces *bZIP60ΔN* transcripts encoding a shorter version of bZIP60 lacking the N-terminal 50 amino acids (aa) (S8A Fig).

### *bzip60-1* Is a Knockdown Mutant and *bzip60-2* a *bZIP60* Splicing Knockout Mutant

To characterize the two types of shorter *bZIP60* mRNAs of *bzip60-1* and *bzip60-2* mutants, we further analyzed *bZIP60* mRNA expression and splicing in the wild type and mutants. Since the IRE1-mediated unconventional splicing could be detected in *Arabidopsis* flowers under unstressed conditions [35], we isolated RNAs from opened and unopened flowers, according to a previous report [43]. The expression levels of *bZIP60* in the wild type and *bZIP60ΔN* in the *bzip60-1* mutant as well as the *bZIP60ΔC* in the *bzip60-2* mutant were quantitatively determined using a primer set sitting between MF and MR (Fig 4A, S1 Table). It was found that, in both types of flowers, the *bZIP60* level in the wild type was significantly higher than the *bZIP60ΔN* level in the *bzip60*-1 mutant (S9A Fig, *P*<0.01). The *bZIP60ΔC* transcript in the *bzip60-2* mutant accumulated to a level similar to *bZIP60* mRNA in the wild type (S9A Fig). Therefore, T-DNA insertion did not affect *bZIP60ΔC* expression in the *bzip60-2* mutant, but greatly inhibited the *bZIP60ΔN* expression in the *bzip60-1* mutant. It should be pointed out that *bZIP60* mRNA in the wild type, *bZIP60ΔN* mRNA in the *bzip60-1* mutant, and *bZIP60ΔC* mRNA in the *bzip60-2* mutant were all up-regulated in opened flowers in comparison with unopened flowers (S9A Fig).

Analysis of *bZIP60* mRNA splicing revealed that *bZIP60* S was not detected in both types of flowers in the *bzip60-2* mutant (S9B Fig). As for the *bzip60-1* mutant, the *bZIP60ΔN* S level of the unopened flowers was significantly lower than the *bZIP60* S level in the wild type (S9B Fig, *P*<0.001). However, in the wild type and *bzip60-1* plants, the level of *bZIP60* S (*bZIP60ΔN* S for *bzip60-1*) was significantly higher in unopened flowers than in opened ones (S9B Fig, *P*<0.001 in wild type and *P*<0.01 in *bizp60-1*), suggesting that the splicing of *bZIP60* mRNA might be part of the scheduled organelle development programs.

Previous publications have shown that treatment of *Arabidopsis* with ER stress agents, such as DTT and Tm, induces *bZIP60* mRNA splicing [15,22,30]. We thus also analyzed *bZIP60* mRNA splicing in the *bzip60-1* and the *bzip60-2* seedlings treated with DTT or Tm. After treatment with ER stress agents DTT or Tm, the *bzip60-1* seedlings rather than the *bzip60-2* seedlings produced the detectable spliced form of *bZIP60* mRNA, although the level of *bZIP60ΔN* S in the *bzi60-1* mutant was lower than that of *bZIP60* S in the wild type (S9C Fig). Clearly, *bZIP60* splicing is eliminated in the *bzip60-2* mutant but only reduced in the *bzip60-1* mutant.

It has been reported that a pair of kissing hairpin loops with three conserved bases in each loop is the recognition sites of IRE1 (S5 and S10A Figs) [15]. Since the spliced form of *bZIP60* could be detected in the *bzip60-1* mutant, it is possible that *bZIP60ΔN* mRNA could still be able to fold into twin kissing loops. To test this idea, we predicted the secondary structure of *bZIP60* mRNA using M-Fold [41]. Among 29 predicted different free energy forms of RNA structures, 89.6% of *bZIP60ΔN* contains the conserved double loops, similar to the full-length *bZIP60* mRNA (S10B Fig). Interestingly, the predicted conserved loops are also present in one of *bZIP60ΔN* mRNA species resulting from 5’ RACE which even contains a short batch of nt from the T-DNA (S10B Fig). These data supported the assumption that the truncated *bZIP60* mRNA in the *bzip60-1* mutant is spliced in the same manner as *bZIP60* mRNA in wild type. Taken together these data suggested that the *bzip60-1* mutant is a *bZIP60* expression knockdown mutant whose *bZIP60ΔN* transcripts can be spliced and the *bzip60-2* mutant is a *bZIP60* splicing knockout mutant.

### bZIP60ΔN S Is Targeted to the Nucleus as bZIP60 S

**Fig 5:**
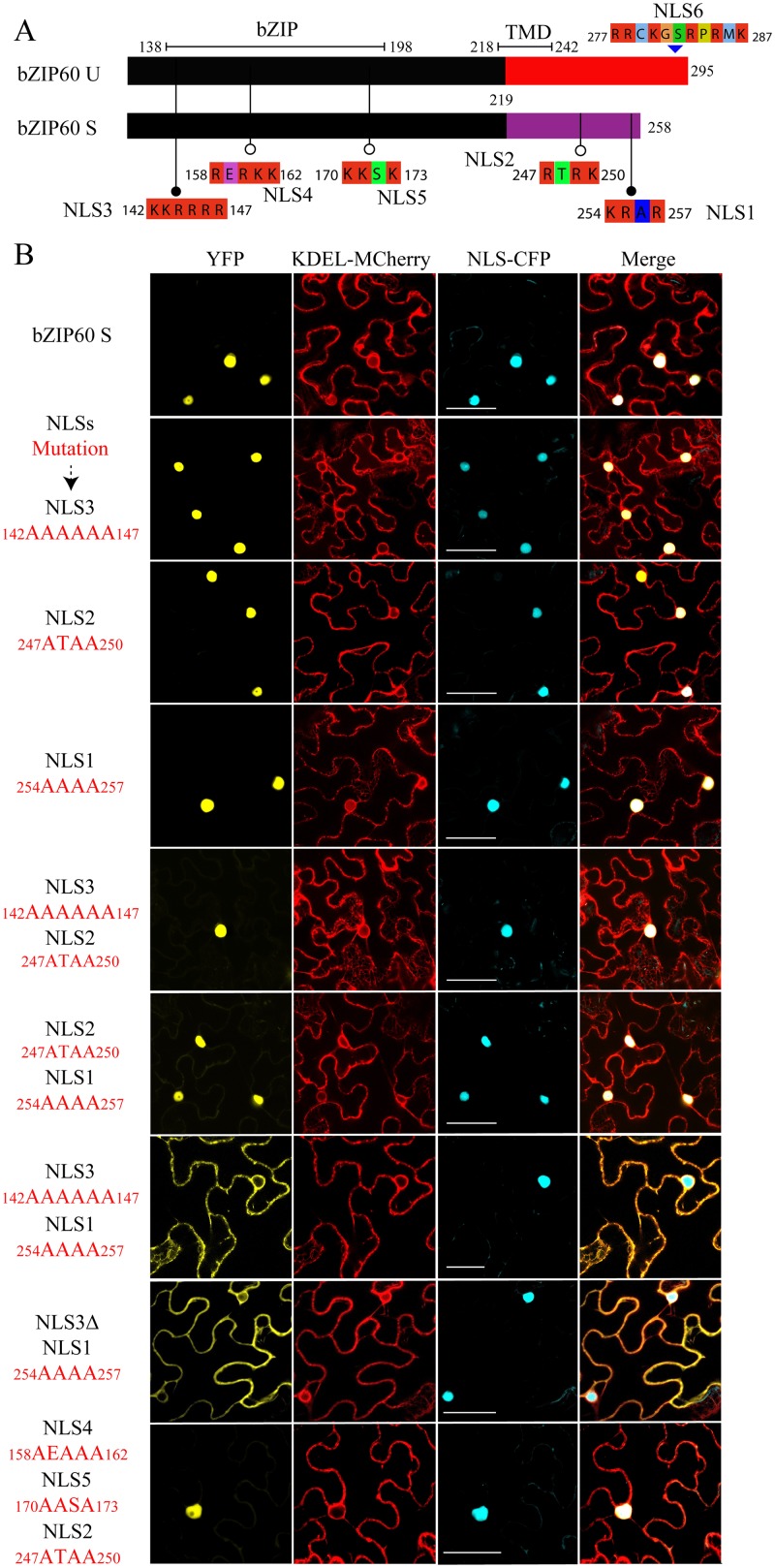
Identification of NLSs of bZIP60 S. **(A)** The predicted NLS motifs distributed in bZIP60 U and bZIP60 S (see S8 Fig). Note that only bZIP60 U contains the TMD, and the NLSs located at the C-terminus of bZIP60 S have been mentioned previously [33] and thus named as NLS1 and NLS2. **(B)** Sub-cellular localization of YFP-bZIP60 S following mutation or deletion (for NLS3) of the predicted NLS(s) of bZIP60 S. KDEL-MCherry and NLS-CFP were used as the ER marker and the nucleus reporter, respectively. Experiments were repeated three times with similar results Bars = 25 μm.

In a recent study, Deng *et al*. have shown that as an active transcriptional factor, bZIP60 S, rather than bZIP60 U, is located in the nucleus [15]. Since bZIP60ΔN S in the *bzip60-1* mutant supports virus infection like full-length bZIP60 S (Fig 3A and 3B), it should at least have capability to enter the nucleus. To prove this assumption, we used NucPred program to position the nucleus localization signal (NLS) in bZIP60 [44]. It was found that both bZIP60 S and bZIP60ΔN S contain five NLS consensus motifs (K/RR/KxR/K) (Figs 5A and S8A) [45]. Among the five predicted NLS motifs, NLS2 and NLS1 are produced due to the splicing-mediated frame-shift, and other three NLSs (NLS3, NLS4 and NLS5) are also present in bZIP60 U and bZIP60ΔN U (Figs 5A and S8A). Further analyses showed that NLS1, NLS3 and NLS4 are highly conserved in at least 20 plant bZIP60 homologues (S11 Fig). Although the unspliced forms of bZIP60 and bZIP60ΔN also contain another conserved NLS motif (NLS6) downstream of the TMD motif, it is absent in the spliced forms of bZIP60 and bZIP60ΔN and thus was not included in this study (Figs 5A, S8A and S11). Mutation analyses showed that none of mutated NLS3, NLS2 or NLS1 alone prevented bZIP60 S from entry into the nucleus (Fig 5B). Also, introduction of a double mutation into NLS3 and NLS2 or NLS2 and NLS1 of the bZIP60 S did not affect its nucleus-targeting (Fig 5B). However, either a double mutation of NLS3 and NLS1 or the deletion of NLS3 plus mutation of NLS1 of the bZIP60 S compromised its nucleus-targeting, leading to co-localizing with an ER labeling marker (Fig 5B). Moreover, a triple mutation of NLS4, NLS5 and NLS2 did not arrest the localization of bZIP60 S into the nucleus (Fig 5B). These data indicated that either NLS3 or NLS1 can direct the targeting of bZIP60 S protein into the nucleus, and that none of NLS4, NLS5 or NLS2 is a functional nucleus-targeting signal motif. We therefore concluded that bZIP60 S and bZIP60ΔN S both have functional NLSs (Figs 5A and S8A).

### Homodimerization of bZIP60 S and bZIP60ΔN S

**Fig 6:**
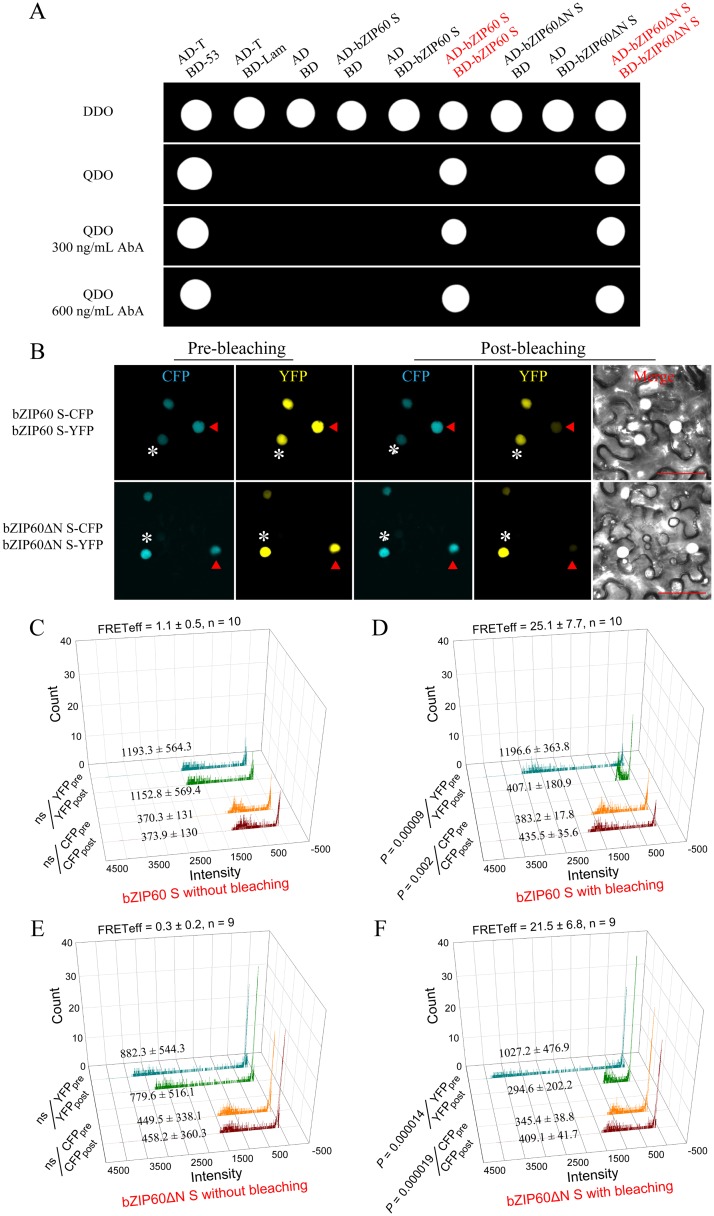
Homodimerization of bZIP60 S and bZIP60ΔN S. **(A)** Self-interactions of bZIP60 S and bZIP60ΔN S in the Y2H assay. Homodimerziation was examined by yeast growth on a QDO medium in the presence of AbA. Note that both bZIP60 S and bZIP60ΔN S showed a strong homo-interaction, indicated by yeast growth even under higher concentration AbA (also see S12A Fig). Data are representative of three repeats. **(B)** Localization and self-interactions of bZIP60 S and bZIP60ΔN S *in vivo* by photo-bleaching assay. Images were captured from CFP and YFP channels before and after YFP-fusion proteins photo-bleaching using a 514 nm beam at 100% output power. Note that the donor fluorescence was increased following photo-bleaching the acceptor (red triangles), whereas it kept constant without the acceptor photo-bleaching (white asterisks). Bars = 50 μm. **(C)**, **(D)**, **(E)** and **(F)** Emission of donor and acceptor images without (**C** and **E**) or with (**D** and **F**) photo-bleaching FRET. The average fluorescence intensity per photon of pre- and post-bleaching images from donor and acceptor was presented, and the FRET efficiency was calculated based on at least 9 independent photo-bleaching results.

It is well known that bZIP proteins form homodimers and/or heterodimers to regulate gene transcription [23,46]. Although bZIP60 without C-terminus (aa 218–258) can heterodimerize with bZIP28 [23], it remains unconfirmed whether bZIP60 S homodimerizes. In addition, given that bZIP60ΔN S contains functional NLSs, and the *bzip60-1* mutant develops typical viral symptoms like the wild type (Figs 3A, 3B, 5 and S8), the bZIP60ΔN S was surmised to behave the same as the full-length bZIP60 S. To verify this assumption, we first examined if bZIP60 S or bZIP60ΔN S self-interacts using the gold yeast two-hybrid (Y2H) system. In yeast, bZIP60 S and bZIP60ΔN S were shown to have no auto-activation and toxicity effects (Fig 6A). When bZIP60 S and bZIP60ΔN S served as both bait and prey, bZIP60 S and bZIP60ΔN S both indeed showed a strong tendency to homodimerize even under high-stringent selection conditions (QDO medium plus a high concentration of AbA) (Figs 6A and S12A).

Next, we employed fluorescence resonance energy transfer (FRET) to further determine the self-interactions of bZIP60 S or bZIP60ΔN S in living cells. As expected, bZIP60 S and bZIP60ΔN S both were located in the nucleus, co-localized with an NLS-tagged reporter (S12B Fig). However, no FRET signal was evident in cells co-transformed with bZIP60 S-CFP and NLS-YFP or the reciprocal combination (S12B Fig). The same results were also obtained from co-transformation with bZIP60ΔN S-CFP and NLS-YFP or the reciprocal combination (S12B Fig). Nevertheless, a strong FRET emission was observed in cells co-expressing bZIP60 S-CFP and bZIP60 S-YFP proteins or co-expressing bZIP60ΔN S-CFP and bZIP60ΔN S-YFP proteins (S12B Fig). The results were further corroborated by the FRET acceptor photo-bleaching assay. After photo-destruction of bZIP60 S-YFP energy acceptor, a significant increase in the fluorescent intensity of bZIP60 S-CFP was observed, indicating energy transfer between bZIP60 S-CFP and bZIP60 S-YFP (Fig 6B and 6D, red triangles, *P* = 0.002). Similarly, the energy transfer between bZIP60ΔN S-CFP and bZIP60ΔN S-YFP was also detectable with a FRET efficiency similar to that found in the combination of bZIP60 S-CFP and bZIP60 S-YFP (Fig 6B, 6D and 6F, red triangles). In control cells without photo-destruction of bZIP60 S-YFP and bZIP60ΔN S-YFP, no significant change in fluorescence intensity of bZIP60 S-CFP and bZIP60ΔN S-CFP could be observed (Fig 6B, 6C and 6E, white asterisks). These results indicated that bZIP60 S and bZIP60ΔN S both homodimerize.

### Either bZIP60 S or bZIP60ΔN S Can Rescue the Virus Suppression Phenotype

**Fig 7:**
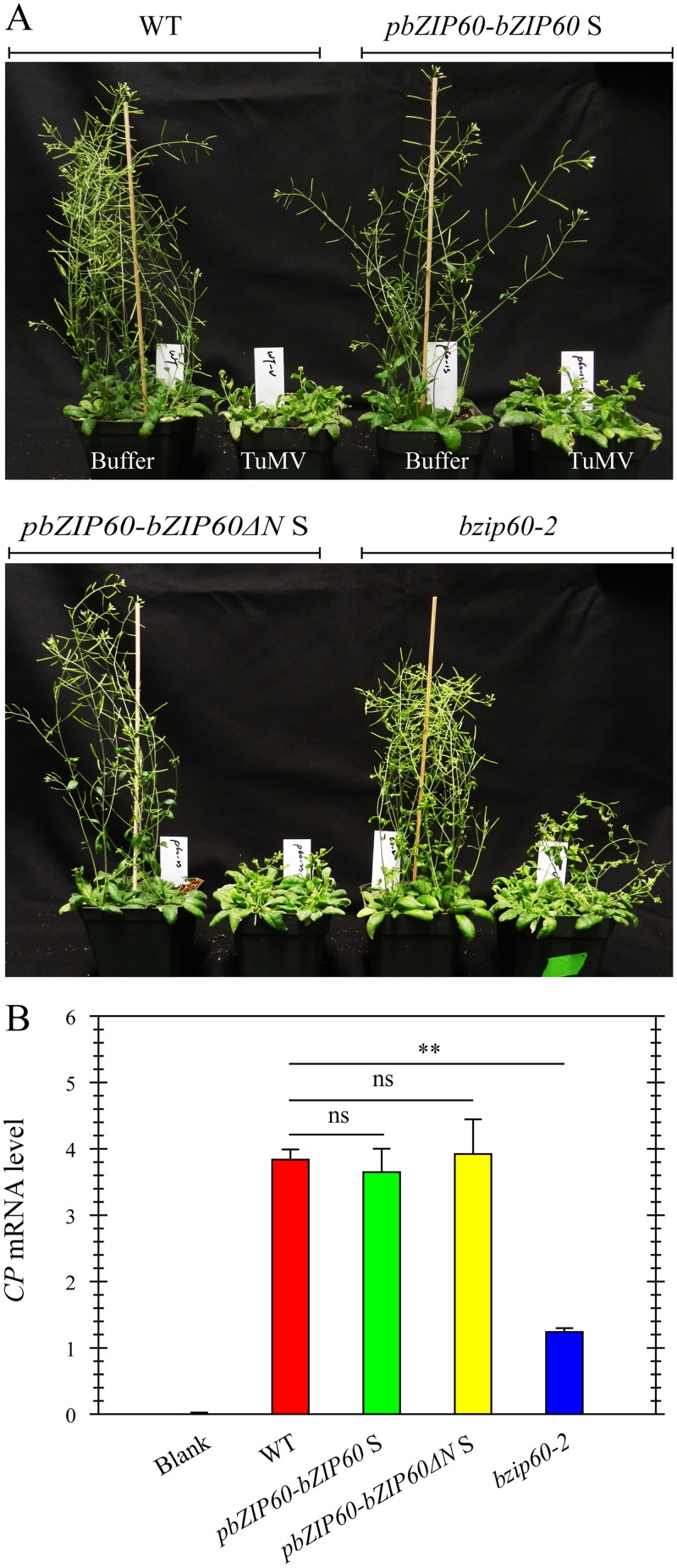
Either bZIP60 S or bZIP60ΔN S can rescue the virus suppression phenotype of the *bzip60-2* mutant. **(A)** Phenotypes of the wild-type, the transgenic lines of *pbZIP60-bZIP60* S and *pbZIP60-bZIP60ΔN* S and the *bzip60-2* mutant at 18 dpi after inoculation with buffer or TuMV. Note that both transformants developed typical TuMV symptoms, compared to the wild type and *bzip60-2* mutant. **(B)** qRT-PCR analysis of TuMV *CP* mRNA levels. At 18 dpi after inoculation with buffer or TuMV, RNA was extracted from systemically infected leaves, and qRT-PCR was carried out. *Actin II* was used as an internal control. Data represent means with SD of three biological replicates. ** *P*<0.05, unpaired two-tailed Student’s test. ns, non-significant.

To prove that the mild viral symptoms phenotype of the *bzip60-2* mutant is caused by the loss-of-function of bZIP60 S, and bZIP60ΔN S fulfills the same function as bZIP60 S in TuMV infection, we complemented the *bzip60-2* mutant using bZIP60 S and bZIP60ΔN S under the control of the native promoter (*pbZIP60*-bZIP60 S and *pbZIP60-*bZIP60ΔN S). We found that, in response to TuMV, the transgenic lines with *bZIP60ΔN* S or *bZIP60* S developed the same typical viral symptoms as the wild type (Fig 7A). Quantitative analyses of virus accumulation revealed that only the *bzip60-2* mutant produced much fewer viruses than the wild type (Fig 7B, *P*<0.01), and no difference in virus accumulation could be detected among the wild type and the transgenic lines carrying either *bZIP60ΔN* S or *bZIP60* S (Fig 7B). These data unambiguously proved that either bZIP60ΔN S or bZIP60 S rescues the virus suppression phenotype of the *bzip60-2* mutant. We therefore concluded that the biotic stress-resistance phenotype of the *bzip60-2* mutant arise from the loss-of-function mutation in bZIP60 S, and bZIP60ΔN S has the same function as bZIP60 S in TuMV infection in plants.

### Loss-of-Function of IRE1A and IRE1B Inhibits Viral Infection

**Fig 8:**
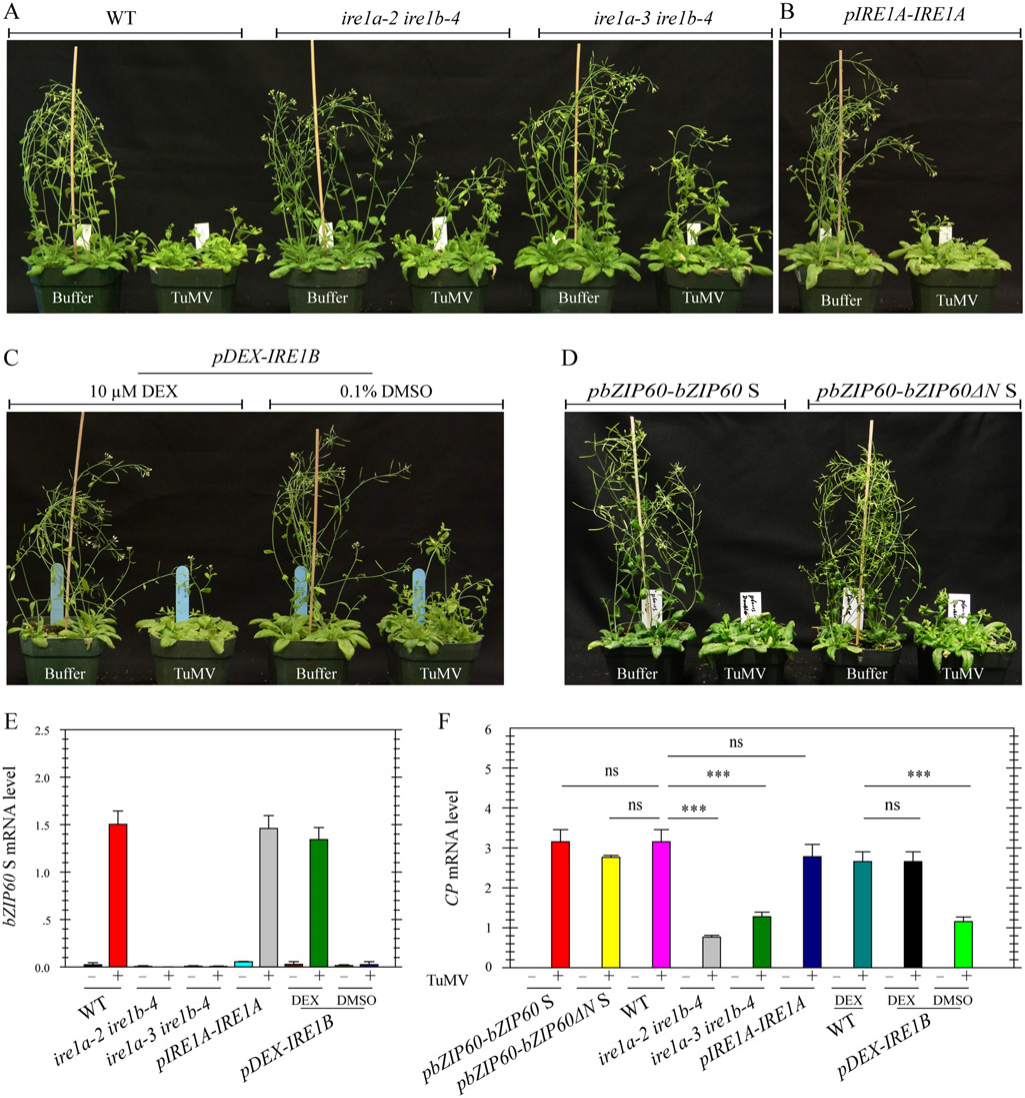
Loss-of-function of *IRE1A* and *IRE1B* inhibits viral infection. **(A)** Phenotypes of the wild type and the two different *ire1a ire1b* double mutants (*ire1a-2 ire1b-4* and *ire1a-3 ire1b-4*) at 18 dpi after inoculation with buffer or TuMV. Note that both double mutants developed slight TuMV symptoms, compared to the wild type and the single mutants (see S13 Fig). **(B)**, **(C)** and **(D)** Complementation of *ire1a-3 ire1b-4* by *IRE1A* under the control of its native promoter **(B)**, *IRE1B* under the control of a DEX-inducible promoter **(C**), or bZIP60 S and bZIP60ΔN S under the control of *bZIP60* promoter **(D)** rescued the virus suppression phenotype of *ire1a-3 ire1b-4*. For dexamethasone (DEX) treatment, 0.1% DMSO (the vehicle of DEX) and DEX (10 μM) were spared onto seedlings 3 d prior to TuMV infection and every 3 d during the whole infection period. **(E)** and **(F)** qRT-PCR analyses of *bZIP60* S **(E)** and TuMV *CP* **(F)** in the wild type, two double mutants and transgenic lines. At 18 dpi after inoculation with buffer or TuMV, RNA was extracted from systemically infected leaves, and qRT-PCR was carried out. *Actin II* was used as an internal control. Data represent means with SD of three biological replicates. *** *P*<0.01, unpaired two-tailed Student’s test. ns, non-significant.

To definitively establish the role of IRE1-*bZIP60* S signaling pathway in the development of viral symptoms, two *ire1a ire1b* double mutants (*ire1a-2 ire1b-4* and *ire1a-3 ire1b-4*) were inoculated with TuMV since both IRE1A and IRE1B have been suggested to process bZIP60 mRNA [15,28,30]. Under TuMV attack, virus-induced symptom development in these double mutants was markedly delayed and viral accumulation was also significantly inhibited, compared with the wild type (Fig 8A and 8F, *P*<0.001), whereas the three single mutants displayed typical viral symptoms like the wild type (S13 Fig). In contrast to an increased level of *bZIP60* S mRNA in TuMV-infected wild type plants, no *bZIP60* S mRNA was detectable in the two *ire1a ire1b* double mutants (Fig 8E). The vanished *bZIP60* S mRNA in the *ire1a-3 ire1b-4* double mutant was largely restored following transformation with *IRE1A* under the control of its native promoter or with *IRE1B* under the control of a *DEX*-inducible promoter in the presence of DEX (Fig 8E). In parallel, TuMV infection was also rescued by complementation of the *ire1a-3 ire1b-4* double mutant with IRE1A or IRE1B alone (Figs 8B, 6C and 8F). Importantly, the virus suppression phenotype of the *ire1a-3 ire1b-4* double mutant could also be rescued by introducing *bZIP60* S or *bZIP60ΔN* S into the double mutant (Fig 8D and 8F). Therefore, it was concluded that the IRE1A or IRE1B mediated *bZIP60* splicing is essential for TuMV infection.

### bZIP60 S and bZIP60ΔN S Are Functionally Complementary with HAC1p S in Yeast

**Fig 9:**
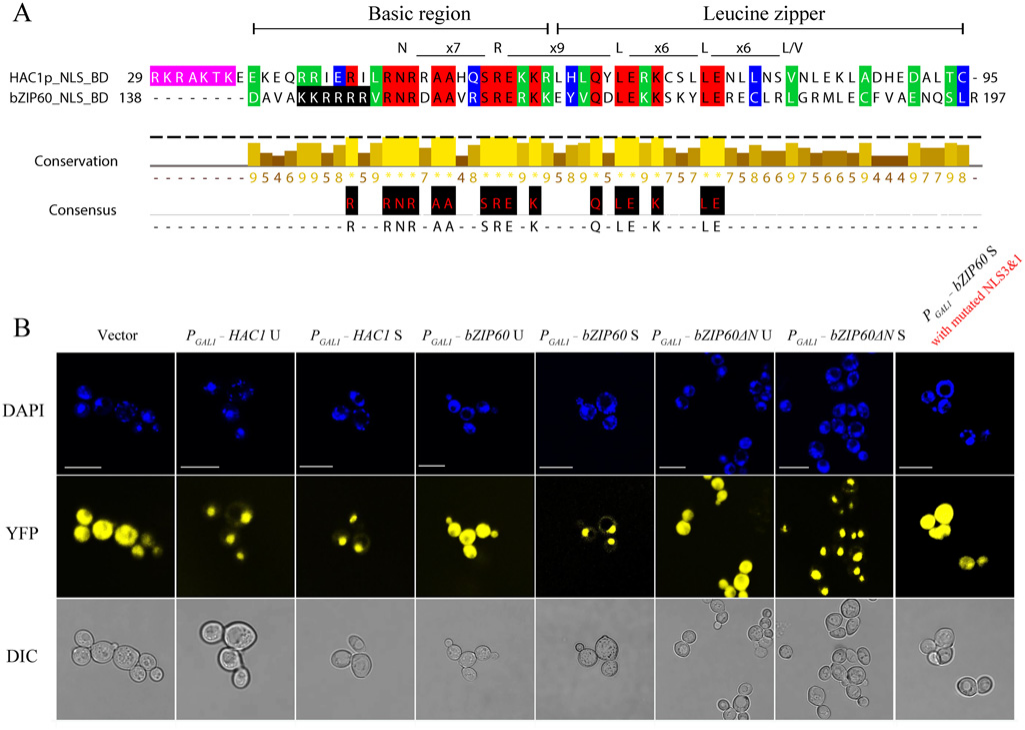
Both bZIP60 S and bZIP60ΔN S are localized to the nucleus in yeast. **(A)** The amino acid sequence of the bZIP domain is highly conserved between HAC1p and bZIP60. The identical residues are highlighted with red, and conserved residues with a conservation threshold of 9 and 8 are shaded with green and blue, respectively. A consensus sequence is given below with a histogram. A schematic of the bZIP domain consensus is shown above by extremely conserved residues and distance [54]. The typical bZIP domain contains basic region and leucine zipper. Note that the NLS shaded in pink in HAC1p, whereas the NLS shaded in black in bZIP60. **(B)** Detection of subcellular localization of HAC1p and bZIP60 by confocal imaging of CRY1 *Δhac1*::TRP strains. The transformed yeast cells with 2-micron plasmids were induced by galactose in the presence of 2 mM DTT. At 8 h post-induction, the yeast cells were stained with DAPI for 1 h and processed for confocal observation. Note that HAC1p U, HAC1p S, bZIP60 S and bZIP60ΔN S localized to the nucleus indicated by DAPI staining, whereas bZIP60 U, bZIP60ΔN U as well as bZIP60 S with mutated NLS3 and NLS1 displayed YFP signal throughout the cytoplasm, like the empty vector control. It is worth mentioning that DAPI also stains the mitochondria. Bars = 10 μm.

Since bZIP60ΔN S and bZIP60 S have equivalent functions in TuMV infection, a biotic stress (Figs 3, 7 and 8), it is very tempting to assume that they have similar function in abiotic stress. In order to prove this, we used a yeast complementation system developed based on *HAC1*-deficient yeast strains (CRY1 *Δhac1p*::TRP) [10,47]. Both HAC1p and bZIP60 contain a conserved DNA binding domain (BD or bZIP domain) (Figs 9A and S8). Moreover, like HAC1p, bZIP60 S and bZIP60ΔN S have a functional NLS [45] that is also located immediate upstream of the BD domain (Figs 5 and S8, NLS3), i.e., RKRAKTK in HAC1p (Fig 9A, shaded in pink) [48] and KKRRRR in bZIP60 (Fig 9A, shaded in black). cDNAs encoding HAC1p U, HAC1p S, bZIP60 U, bZIP60 S, bZIP60ΔN U and bZIP60ΔN S were cloned into a yeast expression vector in frame fused to the C-terminus of YFP under the control of a *GAL1* inducible promoter. In yeast cells, in the presence of 2% galactose and 2 mM DTT, the cells expressing YFP-HAC1 U and YFP-HAC1 S displayed nuclear fluorescence, indicated by 4’, 6-Diamidino-2-phenylindole (DAPI), whereas YFP alone from the empty vector distributed throughout the cytoplasm (Fig 9B). Both bZIP60 S and bZIP60ΔN S tagged with YFP at their N-termini were efficiently targeted to the nucleus (Fig 9B), whereas the fusion proteins YFP-bZIP60 U and YFP-bZIP60ΔN U were found in the cytoplasm (Fig 9B). This was likely due to that the unspliced forms of bZIP60 contain a TMD that is absent in the spliced forms of bZIP60 (S8 and S11 Figs). In addition, consistent with the results obtained in *N*. *benthamiana*, the bZIP60 S protein with mutated NLS3 and NLS1 was found in the cytoplasm in yeast (Figs 5B and 9B).

**Fig 10:**
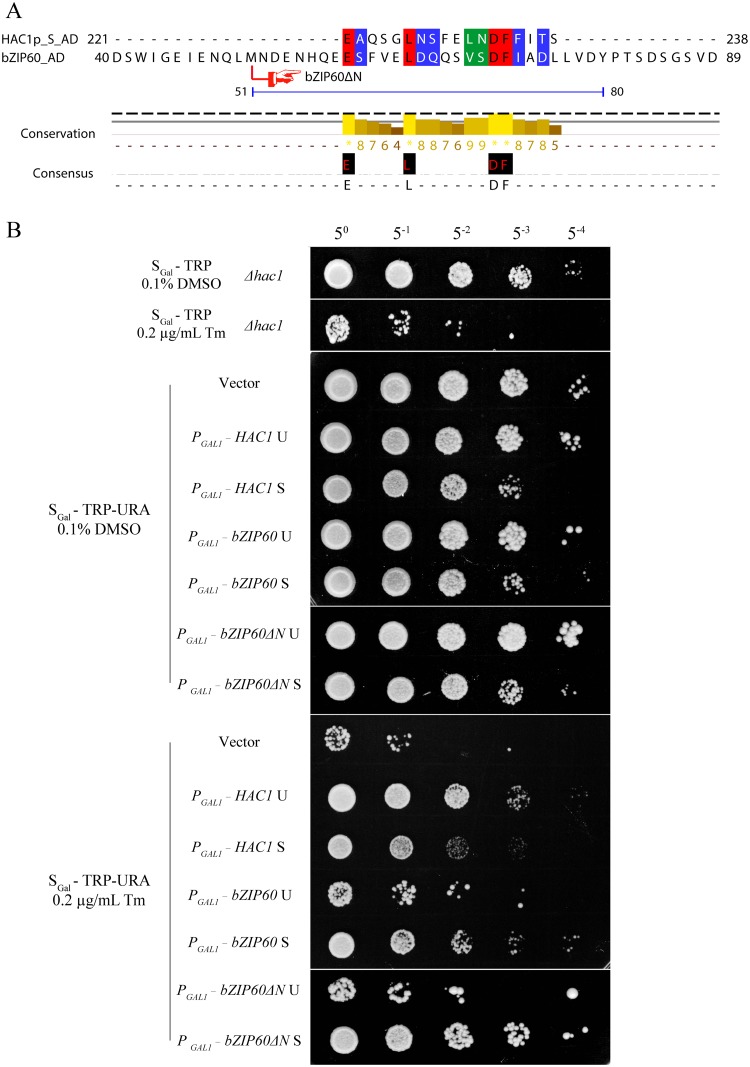
Either bZIP60 S or bZIP60ΔN S can rescue *HAC1*-deficient yeast under ER stress. **(A)** Alignment of the amino acid sequence of the ADs between HAC1p S and bZIP60. A region corresponding to amino acids 59–76 in the AD of bZIP60 was found to be highly identical to the AD of HAC1p S. The identical residues are highlighted with red, and conserved residues with a conservation threshold of 9 and 8 are shaded with green and blue, respectively. A consensus sequence is given below with a histogram. Note that both bZIP60 S and bZIP60ΔN S contain the conserved AD region (see S8 Fig). **(B)** Functional complementation in CRY1 *Δhac1*::TRP strains with HAC1p and bZIP60. The untransformed or transformed yeast cells with CEN-ARS plasmids, which were grown in the raffinose-containing medium for 8 h, were switched to galactose-containing medium for induction from OD_600_ = 0.3. At 10 h post-induction, the cells were normalized to an OD_600_ = 1.0 and 5-fold serial dilutions were spotted on galactose-containing plates in the presence of 0.1% DMSO (control) or 0.2 μg/mL Tm. The plates were kept at 30°C for 48 h. Note that the expression of *bZIP60* S and *bZIP60ΔN* S, not *bZIP60* U or *bZIP60ΔN* U, inhibits yeast growth under normal condition but increases ER stress tolerance, and the induction of both *HAC1* U and *HAC1* S leads to retarded growth and enhanced ER tolerance (see Fig 11C).

**Fig 11:**
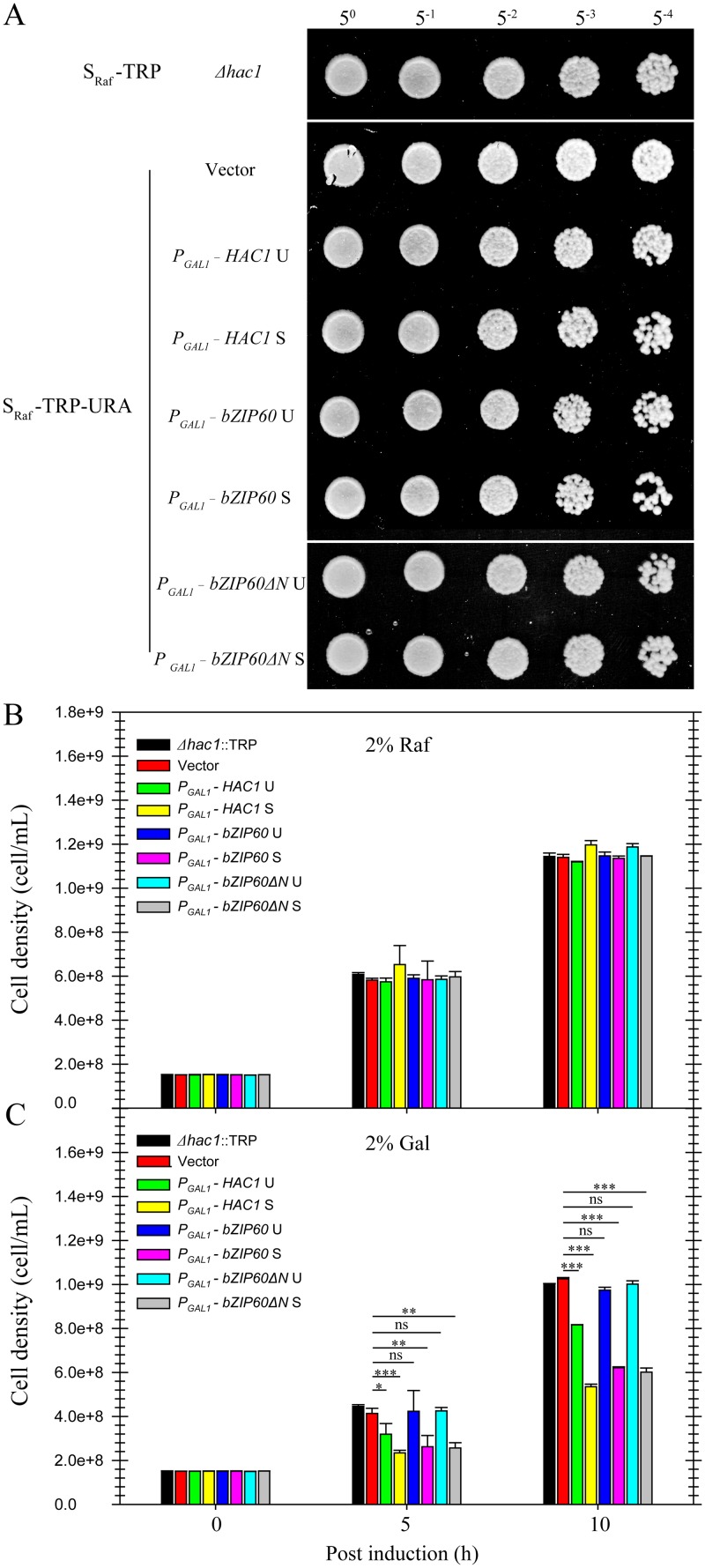
Effects of constitutive expression of *HAC1* and *bZIP60* on yeast growth. **(A)** to **(C)** The untransformed or transformed CRY1 *Δhac1*::TRP cells, which were grown in the raffinose-containing medium for 8 h, were switched to galactose- or raffinose-containing medium for culture from OD_600_ = 0.3. **(A)** After 10 h culture in the presence of raffinose, the cells were normalized to an OD_600_ = 1.0 and 5-fold serial dilutions were spotted on raffinose-containing plates. The plates were kept at 30°C for 48 h. **(B)** and **(C)** Quantitative measurement of the effects of constitutive expression of HAC1p and bZIP60 on yeast growth in the presence of raffinose **(B)** and galactose **(C)**. After 5 and 10 h culture, the yeast cell density was determined by measuring the OD_600_ (1 OD_600_ = 5e+8). Note that the expression of *bZIP60* S and *bZIP60ΔN* S, not *bZIP60* U or *bZIP60ΔN* U, inhibits yeast growth, whereas the induction of both *HAC1* U and *HAC1* S leads to retarded growth (see Fig 10B). Data represent means with SD of three experiments. * *P*<0.05, ** *P*<0.01, *** *P*<0.001, unpaired two-tailed Student’s test. ns, non-significant.

It has been reported that the last 18 aa (residues 221–238) in HAC1p S is a domain for transcription activation (AD), which results from the unconventional splicing to remove 252 nt (S8 Fig) [18]. In *Arabidopsis*, the transcriptional activation activity of bZIP60 is located to aa 41–80 (S8 Fig) [22,49]. Comparison of the two ADs revealed a highly conserved motif corresponding to aa 59–76 of bZIP60 S, which is also present in bZIP60ΔN S (Figs 10A and S8). Since both bZIP60 S and bZIP60ΔN S contain NLS, AD and BD domains as HAC1p, functional complementation was performed to test whether bZIP60 S and bZIP60ΔN S could execute the functions of HAC1p in yeast. Considering that bZIP60ΔC from the *bzip60-2* mutant also contains AD and BD domains as well as a functional NLS (S8 Fig, NLS3), it was therefore included in the functional complementation assay. Here, we designed two types of bZIP60ΔC, i.e., bZIP60ΔC1 and bZIP60ΔC2 (S8A and S14 Figs). cDNAs of HAC1p and bZIP60 were expressed in the *Δhac1*::TRP cells using a CEN-ARS plasmid containing a GAL1 inducible promoter. Compared to the control yeast grown in S_Gal_-TRP with 0.1% DMSO, the *Δhac1p*::TRP cells displayed an obvious growth defect under 0.2 μg/mL Tm treatment (Fig 10B). Intriguingly, bZIP60 S and bZIP60ΔN S, rather than bZIP60 U, bZIP60ΔN U and bZIP60ΔCs, successfully rescued the ER stress phenotype like HAC1p U and HAC1p S (Figs 10B and S14B). As expected, the bZIP60 S protein with mutated NLS3 and NLS1 failed to rescue the Tm-sensitive phenotype (S14B Fig). Previous studies have shown that constitutive expression of HAC1p S has an adverse effect on yeast growth under normal conditions [12,50]. Consistent with these results, the yeast cells expressing bZIP60 S or bZIP60ΔN S, but not bZIP60 U, bZIP60ΔN U nor bZIP60ΔCs, also exhibited limited growth in the presence of 2% galactose (Figs 11C and S14A). As expected, the yeast cells displayed normal growth in non-inducible medium (Figs 11A, 11B and S14B). Together, the results suggested that bZIP60 S or bZIP60ΔN S functions like HAC1p to rescue the ER-stress sensitive phenotype in yeast.

## Discussion

### bZIP60 S of *Arabidopsis* Is a Functional Homolog of Yeast HAC1p

In this work, we found that the *bzip60-2* mutant displayed an anti-biotic stress phenotype, i.e., inhibition of viral infection, whereas the other independent mutant *bzip60-1* exhibited the same susceptible phenotype as wild type plants (Fig 3). Our experimental evidence revealed that both *bzip60-1* and *bzip60-2* mutants are non-RNA null mutants and produce an N-terminal truncated mRNA (*bZIP60ΔN*) and a C-terminal truncated mRNA (*bZIP60ΔC*), respectively (Figs 4, S6, S7 and S8A). Our data also showed that the *bzip60-2* mutant is a splicing knockout mutant, whereas the *bzip60-1* mutant is an expression knockdown mutant and *bZIP60ΔN* mRNA can undergo splicing (Figs 3, S7, S8A, S9 and S10). As *bZIP60ΔN* mRNAs contain an in-frame start codon 150 nt downstream of AUG of wild type *bZIP60* ORF (Figs 4A and S7), the bZIP60ΔN S protein in the *bzip60-1* mutant, like bZIP60 S, bears functional AD and BD domains as well as the intact C-terminus (Figs 9, 10 and S8A) [22,49]. Moreover, bZIP60 S and bZIP60ΔN S both contain two functional NLSs (Figs 5, 6B, S8A, S11 and S12B), i.e., NLS1 at their C-terminal regions, which is generated from the splicing-mediated frame-shift, and NLS3 located upstream of their BD domains (S8A Fig). Like bZIP60 S, bZIP60ΔN S self-interacts to homodimerize (Figs 6 and S12). Taken together, we concluded that bZIP60ΔN S executes the equivalent function as the wild type bZIP60 S. This conclusion is strengthened by two further lines of evidence obtained in complementation assays in plants under biotic stress and in yeast under abiotic stress (Figs 7, 10 and 11; see discussion below).

The bZIP60ΔN S-producing *bzip60-1* mutant has long been considered as an RNA-null mutant to ascertain the behavior of the UPR, but many confusing findings have been generated. First, it has been reported that a low level of truncated *bZIP60* mRNA is present in the *bzip60-1* mutant [42,51] and the expression of several UPR marker genes is not significantly affected in this mutant [42]. Second, in response to ER stress, the expression of the UPR marker genes, including *BiP1*, *BiP2*, *and BiP3*, is up-regulated in the *bzip60-1* mutant, like the wild type [15,52], and the *bzip60-1* mutant can activate the ER stress response [53]. Third, under ER stress, the *bzip60-1* mutant develops only a modest ER stress phenotype similar to the wild type controls, whereas the double mutant of *IRE1A* and *IRE1B* displays a marked stress injury [23,28]. Finally, the gametes bearing the triple mutation of *IRE1A*, *IRE1B* and *bZIP28* are lethal, but the double *bzip60-1 bzip28-2* mutant not [35]. Therefore, the finding of bZIP60ΔN S as a functional derivative of the full-length bZIP60 S presented in this work unambiguously helps to clarify these long-standing confusions.

As *HAC1* is the splicing target of IRE1 in yeast, the identification of *bZIP60* mRNA as the substrate of IRE1 in *Arabidopsis* raises the question if bZIP60 S and bZIP60ΔN S are the functional homolog of yeast HAC1p [15]. To answer this question, we first determined if bZIP60 protein derivatives localize to the nucleus of yeast. We found that, like yeast HAC1p U and HAC1p S, bZIP60 S and bZIP60ΔN S were predominantly concentrated in the nucleus in yeast under DTT treatment (Fig 9B). However, the bZIP60 S with mutated NLS3 and NLS1 accumulated in the cytoplasm and failed to enter the nucleus in plant cells and in yeast (Figs 5 and 9B), suggesting that the mechanisms of nuclear import used for bZIP proteins may be highly conserved across kingdoms. This notion is also in agreement with the finding that both bZIP60 and HAC1p carry a functional NLS (NLS3 for bZIP60) at the similar N-terminal region of their BD domains, and this NLS is highly conserved among bZIP60 homologs from at least 20 plant species (Figs 5, 9 and S11). It should be pointed out that bZIP60 U and bZIP60ΔN U were located in the cytoplasm in yeast, even though they contain a functional NLS (NLS3) (Figs 9B and S8). The exclusion of bZIP60 U and bZIP60ΔN U from the nucleus may be attributed to the TMD that anchors the bZIP60 U and bZIP60ΔN U proteins to the ER membrane in yeast (Figs 9B and S8), consistent with the findings obtained in *Arabidopsis* and tobacco suspension cells [15,52]. The removal of the small 23-bp intron of bZIP60 U and bZIP60ΔN U leads to a frame-shift that eliminates the TMD and acquires another functional NLS (NLS1), enabling bZIP60 S and bZIP60ΔN S to be targeted to the nucleus (S5 and S11 Figs) [15]. Therefore, we concluded that yeast IRE1 cannot splice *bZIP60* U into *bZIP60* S which is translated into the nucleus-targeting bZIP60 S. Given that the plant IRE1 could not splice the yeast *HAC1* mRNA either in *Arabidopsis* protoplasts [26] or in yeast itself (S15 Fig), we speculate that it is the secondary structure difference between *bZIP60* and *HAC1* mRNAs that does not allow for splicing in the heterologous systems.

The complementation assay conducted in a *Δhac1* yeast strain demonstrated that, like HAC1p, both bZIP60 S and bZIP60ΔN S, but not bZIP60 U nor bZIP60ΔN U, rescued the ER stress phenotype of *HAC1*-deficient strain (Fig 10B), indicating the two spliced forms are a functional homolog of yeast HACp in abiotic stress tolerance. As bZIP signature-bearing proteins, HAC1p acquires the C-terminal 18-aa AD after IRE1-mediated splicing [18], whereas bZIP60 has the N-terminal, splicing-independent AD (S8 Fig) [49]. In spite of this positional difference, the two transcriptional activation domains share high sequence similarity (Fig 10A). The identification of bZIP60 S and bZIP60ΔN S as a homolog of HAC1p was further corroborated by the yeast growth assay showing that, under normal condition, constitutive expression of either bZIP60 S or bZIP60ΔN S rather than their unspliced forms slowed down yeast growth (Fig 11), a phenotype resulting from the constitutive expression of HAC1p [12,50]. Based on these findings, we propose that although the sequences of the transcription factors, i.e., bZIP60 and HAC1p, and the IRE1-mediated splicing mechanisms have diverged from the last eukaryotic ancestor, the downstream ER stress signaling mechanisms have evolved to functionally converge at least in yeast and plants.

It should be mentioned that although bZIP60ΔC in the *bzip60-2* mutant has all functional NLSs as well as AD and BD domains, it failed to rescue the Tm-sensitive phenotype of *HAC1*-deficient yeast and did not inhibit yeast growth under normal conditions (S8 and S14 Figs). This is most likely due to the fact that it lacks the intact C-terminus (aa 202–258) of the wild type bZIP60 S (S8 Fig). According to a previous report, a truncated bZIP60 without the large C-terminal region (aa 218–258) is not able to homodimerize (S8 Fig), although it can still heterodiemrize with a same truncated bZIP28 [23]. Therefore, the IRE1-mediated *bZIP60* splicing not only leads to the elimination of the TMD and acquisition of a functionally redundant NLS, but also produces a new C-terminal sequence that is essential for bZIP60 S homodimerization to carry out transcriptional regulation. Alternatively, the C-terminal sequence, which is immediately proximal to the BD domain, might be important for the proper formation of the superimposing coiled-coil structure that binds targeted DNAs [54]. Based on these results and analyses, it is reasonable to conclude that bZIP60ΔC in the *bzip60-2* mutant is non-functional.

### IRE1-dependent *bZIP60* Splicing Plays an Important Role in Viral Pathogenesis

As the primary UPR arm, the role of IRE1 and its mRNA substrate in viral infection has been intensively explored in mammalian cells [55,56]. In human hepatoma cells expressing hepatitis C virus (HCV) sub-genomic replicons, IRE1 is activated as indicated by the enhanced *XBP1* S mRNA level, but the transcriptional regulation activity of XBP1 S is inhibited [56,57]. Similar to the case of HCV, infection with murine coronavirus mouse hepatitis virus also causes a progressive increase in *XBP1* S mRNA with very little XBP1 S protein [58]. Therefore, the activation of IRE1 in parallel with the concomitant inhibition of XBP1 S (in either mRNA or protein level or both) has been viewed as an effective strategy utilized by mammalian viruses to cope with the IRE1-*XBP1* branch-mediated antiviral responses [58,59].

In this work, we found that, in response to TuMV infection, the IRE1-*bZIP60* arm of the UPR was activated in both locally and systemically infected leaves, indicated by the accumulation of *bZIP60* S (Figs 1, S1 and S2). We also found that upon TuMV infection, the *bzip60-1* mutant with up-regulated *bZIP60ΔN* S, albeit at a lower level, developed typical viral symptoms and allowed viruses to accumulate at the same level as the wild type (Fig 3). In contrast, the other independent mutant *bzip60-2* without detectable *bZIP60* S significantly inhibited the viral accumulation and remarkably suppressed the development of disease symptoms (Fig 3). We further provided genetic evidence that either *bZIP60* S or *bZIP60ΔN* S could rescue the virus suppression phenotype of the *bzip60-2* mutant (Fig 7). These data clearly indicated that the virus suppression phenotype in the *bzip60-2* mutant arise from the loss-of-function of *bZIP60* S. Consistently, two different double mutants of *IRE1A* and *IRE1B*, in which *bZIP60* U splicing was blocked, displayed reduced levels of viral RNA accumulation and suppressed viral symptom development like the *bzip60-2* mutant (Figs 3 and 8), suggesting that *bZIP60* S, not *bZIP60* U, plays a crucial function in favoring virus infection. The absence of *bZIP60* S and suppression of virus infection in the double mutants of *IRE1A* and *IRE1B* were rescued by complementation with either *IRE1A* or *IRE1B* alone (Fig 8). Moreover, the virus suppression phenotype in the *ire1a-3 ire1b-4* double mutant could also be recovered by the introduction of *bZIP60* S or *bZIP60ΔN* (Fig 8). These results directly demonstrated that IRE1 and its processed *bZIP60* S function as a linear pair in promoting virus infection (Figs 3, 7 and 8). To our knowledge, this is the first report showing that that the projected cognate system of IRE1 and *bZIP60* behaves like a host factor in viral infection in plants. It should be mentioned that although IRE1 and *bZIP60* were both involved as a matched system in viral pathogenesis (Figs 3, 7 and 8), neither the double mutant of *IRE1A* and *IRE1B* nor the *bzip60-2* mutant could completely prevent viral infection (Figs 3 and 8). This result is in accordance with the established conception of the UPR functioning as a buffer or a homeostat to cope with diverse ER stresses [47,60,61].

Nevertheless, the role of IRE1 and *bZIP60* S in virus infection in plants apparently contradicts with the function of their counterparts as an resistance mechanism to some viruses in mammalian cells and to a bacterial pathogen in plants [30,58,59]. This is probably due to the complexity of UPR signaling pathways and speciality of virus-host interactions. Indeed, several recent studies have shown that the UPR could be hijacked by virus to favor viral infection. In lung epithelial cell, influenza A virus activates the IRE1 pathway, with little or no concomitant activation of PERK and ATF6 pathways, and inhibition of IRE1 activity leads to reduced viral replication [62]. In *N*. *benthamiana*, silencing *NtbZIP60* suppresses the expression of the UPR marker genes and reduces *Potato virus X* (PVX) accumulation [38,39]. It is possible that during viral infection, the IRE1-*bZIP60* pathway-mediated UPR may aim to alleviate cytotoxicity by up-regulating ER molecular chaperons since membrane-associated virus replication or accumulation of large amounts of viral proteins can break the homeostatic cellular environment. This reasoning is supported by our observation that TuMV infection induced the accumulation of ER chaperones including BiP3, BiP1/2, CRT and PDI (S2 Fig) as well as by several earlier reports that virus infection up-regulated the expression of *bZIP60* and ER marker genes in plants [63–65].

The increased expression of ER-resident chaperones may further facilitate virus infection through direct involvement in virus infection process [66]. In the single-celled yeast, the host Ssa1/2p molecular chaperone (yeast homologue of HSP70) is required for the assembly of the tombusvirus replicase to enhance viral RNA replication [67,68]. In plants, *HSP70* induced by potyvirus infection is also regulated by the cytoplasmic UPR pathway [69,70]. As a component of a membrane-associated viral ribonucleoprotein complex, HSP70 has a role, together with its co-chaperone CPIP, in preventing the potyviral coat protein from interfering with viral gene expression [66], and the *Hsp70*-*15*-deficient mutant is more tolerant to virus infection [71]. In agreement with these findings, we also found that activation of the UPR in *N*. *benthamiana* through treatment with pharmacological small molecular chaperones did promote TuMV infection (S16 Fig). It is worth pointing out that although *BiP2* was induced by TuMV infection, the *bip2-2* mutant developed normal disease symptoms (S2 and S13 Figs). This is likely due to that molecular chaperones could complement each other or that *bip2-2* might not be a clean knockout mutant [72].

In this work, we found that among 11 viral factors of TuMV, 6K2 significantly induced the splicing of *NtbZIP60* mRNA in *N*. *benthamiana* (Fig 2B). The potyviral 6K2 protein is an integral membrane protein and elicits the formation of ER-derived virus replication factories at ER exit sites [40]. The finding presented here is consistent with previous reports that virus-encoded ER targeting proteins induce the UPR. For instance, among seven proteins encoded by simian virus 5, only the HN glycoprotein that is inserted into the ER is capable of stimulating UPR response [73]. This also holds true for the ER-resident proteins encoded by flaviviruses or retroviruses such as human immunodeficiency virus [57,74]. In *Arabidopsis* and *N*. *benthamiana*, the PVX viral movement protein TGBp3 that also resides in the ER is sufficient to elicit the UPR [39,75]. Therefore, the potyviral 6K2 protein is a new member in the group of virus-encoded ER-targeting and UPR-inducing proteins. However, the mechanism of 6K2 triggering the UPR is yet to be determined. In an attempt to test if 6K2 interacts with IRE1 to activate the UPR, we found no interactions between 6K2 and IRE1 (S17 Fig). It is possible that 6K2 induces the UPR through its physical interaction with the ER or subsequent ER remodelling. Elucidation of the molecular mechanisms by which the viral protein(s) triggers the UPR will certainly advance our understanding of the UPR itself as well as virus-host interactions in general.

### IRE1 and Its Splicing Substrate As a Linear Pair Is Evolutionarily Conserved in Higher Eukaryotes

In yeast, *Δhac1* and *Δire1* mutants exhibit indistinguishable growth phenotypes and share highly correlated gene-expression profiles [7]. Search for additional mRNA substrates of IRE1p using three independent genome-scale methods did not identify any other mRNA except *HAC1* mRNA [76]. Therefore, it is suggested that IRE1p and *HAC1* mRNA have evolved as a matched enzyme-substrate pair to carry out the signal transduction between the ER and nucleus of the UPR [76]. In this report, we showed that IRE1 and *bZIP60* mRNA as a cognate system to determine the viral pathogenesis in *Arabidopsis* (Figs 3, 7 and 8), unambiguously demonstrating that the ancient pair also plays an important role in biotic stress in plants.

Given that the IRE1p and *HAC1* mRNA pathway functions in the simple one-celled eukaryotic organism of yeast as a linear pair during development or stress responses and the IRE1 and *bZIP60* mRNA duet manipulates the virus-host interactions in plants, it is reasonable to propose that the coupling of IRE1 and its splicing substrate is evolutionarily conserved in higher eukaryotes. In *Caenorhabditis elegans*, deletion of either *IRE1* or its splicing target *XBP1* is synthetically lethal with deletion of either ATF-6 or PEK-1, due to a developmental arrest at larval stage 2 [77]. In mammalians, *IRE1* ^-/-^ or *XBP1* ^-/-^ mouse embryo perishes at a similar early stage of gestation (between days 9.5 and 11.5 of gestation), indicating the linear pair of IRE1 and *XBP1* is essential for individual development [13,78]. The conserved property of the linear enzyme-substrate pair may be also reflected by the specificity and uniqueness of IRE1 splicing mRNA substrate. To date, *bZIP60* mRNA is the only known substrate of IRE1A/IRE1B in *Arabidopsis* [22,30,35].

In view of the fundamental roles played by the IRE1p and *HAC1* mRNA pair in yeast, we suggest that the corresponding counterpart pairs in other higher eukaryotes may function in diverse biological processes. However, the existence of functionally redundant genes and overlapping pathways hampers the identification and further elucidation of functions mediated by the IRE1-mRNA substrate pathway. In *Arabidopsis*, the pollen viability of the single UPR pathway mutants is similar to that of wild type plants, whereas the triple mutant *ire1a-2 ire1b-4 bzip28*-2 is lethal, caused by a severe defect in male gametophyte [35]. These observations suggest that the UPR pathways could functionally complement each other for plant development. Similar results have been observed in *C*. *elegans*, in which ATF-6 acts synergistically with PEK-1 to complement the developmental requirement for IRE1-*XBP1* [77]. In this study, we proved that the virus suppression phenotype resulting from dysfunction of the IRE1-*bZIP60* pathway is independent of the S1P/S2P-bZIP17/bZIP28 arm (S13 Fig), directly showing a single branch of the UPR determines a biological process. To our knowledge, this is the first evidence that the single UPR arm functions alone in plants.

It is worth stressing that IRE1-mediated UPR response in higher multicellular organisms is apparently more complex and diverse than that in yeast. In *Arabidopsis*, a double mutant of *IRE1A* and *IRE1B* showed a short-root phenotype [28], but both the *bZIP60* slicing knockout mutant (*bzip60-2*) (S18 Fig) and the *bZIP60* expression knockdown mutant (*bzip60-1*) [35] displayed normal root growth, suggesting that IRE1 may activate other cellular component(s) to execute the regulatory function of IRE1 in root growth. In mammalian cells, IRE1 not only splices *XBP1* mRNA but also activates Jun N-terminal kinase, a serine-directed protein kinase, in response to ER stress in embryonic fibroblasts, and directly interacts with pro-apoptotic factors, such as BAX and BAK, to contribute to apoptosis in ER-stressed cells [78,79]. Most recently, IRE1 has been shown to selectively degrade microRNAs (miRs -17, -34a, -96, and -125b) [80]. These findings suggest that IRE1 may regulate the UPR signaling through protein–protein interaction, mRNA splicing, microRNA degradation and other unknown mechanisms in a multicellular context, even in the single-celled yeast [34,81].

## Materials and Methods

### Plant Materials

*Arabidopsis thaliana* used in this study is in Columbia-0 (Col-0) background, with the exception of *ire1a-3* in Col background as well as of *bzip60-2* and *ire1b-4* in Col-3 *qrt* background. The mutants *bzip60-1* (SALK_050203), *bzip60-2* (SAIL_283_B03), *bzip17* (SALK_104326), *bzip28-2* (SALK_132285), *bip2-2* (SALK_047956), *ire1a-2* (SALK_018112) and *ire1b-4* (SAIL_238_F07) were obtained from the Arabidopsis Biological Resource Center. The mutant *ire1a-*3 (WiscDsLox420D09) and two different double mutants (*ire1a-3 ire1b-4* and *ire1a-2 ireb-4*) were described previously [28,30].

Unless stated otherwise, all *Arabidopsis* plants were grown in a growth chamber with a 14 h photoperiod (100 μmol photons m^-2^ s^-1^) and a relative humidity of 75% at 23/21°C (light/dark). The *N*. *benthamiana* plants were grown in a growth room with a 16 h photoperiod (80–100 μmol photons m^-2^ s^-1^) and a relative humidity of 75% at 22°C.

### Identification of Homozygous Mutants

The homozygous line containing the T-DNA insertion in the gene of interest was screened essentially as described previously [82]. The genotyping primers were listed in S1 Table.

### Virus Infection Assay

TuMV was introduced into plants via either agro-infiltration or mechanical inoculation according to a previous report [82]. For mechanical inoculation, TuMV-infected *N*. *benthamiana* leaves were used as an inoculum.

### RNA Extraction, PCR, RT-PCR and Real-Time RT-PCR

RNA extraction, PCR, RT-PCR and real-time RT-PCR analyses were performed essentially as described previously [28,82]. The primer sets used in this study were listed in S1 Table. The RNAs from yeast CRY1 treated with or without 2 mM DTT were exacted using NucleoSpin RNA II (Clontech) to obtain cDNAs of *IRE1*, *HAC1* U and *HAC1* S.

### Entry Vector Construction

Unless stated otherwise, Phusion High-Fidelity DNA Polymerase (NEB, USA) was used to amplify all DNA sequences using the primer sets listed in S1 Table, and Gateway technology (Invitrogen, USA) was employed to generate plasmids. Coding sequences of HAC1p U, HAC1p S and IRE1p were amplified using yeast cDNA as described above. Coding sequences of bZIP60 U, bZIP60 S, bZIP60ΔN U, bZIP60ΔN S, bZIP60ΔC1, bZIP60ΔC2, IRE1A and IRE1B were amplified using *Arabidopsis* cDNA (cDNA from DTT-treated seedlings was used for amplification of *bZIP60* S and *bZIP60ΔN* S, whereas cDNA from *bzip60-2* seedlings for amplification of *bZIP60ΔC2*). Coding regions of P1, HC-Pro, P3, 6K1, CI, 6K2, NIaVPg, NIaPro, NIb and CP of TuMV were amplified from the TuMV infectious clone [40]. With the exception of pENTR^TM^ 1A Dual Selection vector (A10462, Invitrogen) used for IRE1B, all amplified coding sequences were recombined into pDONR221 via the BP reaction (Invitrogen, USA). The entry vector containing P3N-PIPO was described in our previous work [40]. To highlight the nucleus and to produce donor- and acceptor-only samples (used in FRET assays), constructs bearing *35S*::*NLS-CFP* and *35S*::*NLS-YFP* were created following the BP and LR reactions using the primers listed in S1 Table.

### Transient Expression of Viral Factors in *N*. *benthamiana*

The linearized products of the entry vectors containing virus single factor sequences were recombined into the binary destination vector pEarleygate103 for expression of fusion proteins containing viral factors-GFP or into the vector pMDC43 for expression of GFP-viral factor fusions. The resulting binary destination vectors were electroporated into *Agrobacterium tumefaciens* (GV3101). The GV3101 cells harbouring relevant expression constructs were re-suspended with the infiltration buffer to OD_600_ = 0.3, and then infiltrated into the leaves of 3-week-old *N*. *benthamiana* seedling. At 2.5 dpi, the transient expression of each construct was verified under an inverted confocal microscope (TCS SP2, Leica, Germany) by observing GFP at an excitation wavelength of 488 nm and an emission 510–550 nm, and the agroinfiltrated leaves were harvested for RNA extraction. The experiments were repeated three times, and each treatment contains at least three independent plants.

### Mutation of NLSs in bZIP60 S and Sub-cellular Localization

The mutation of the putative NLSs of bZIP60 S was conducted on the entry vector bearing the cDNA of *bZIP60* S using the primers listed in S1 Table, based on the QuikChange Lightning Site-Directed Mutagenesis Kit (210519, Agilent). The mutated vectors were recombined into the pEarlyGate104 vector via the LR reaction (Invitrogen, USA), to generate constructs with YFP fused to the N-terminus of bZIP60 S under the control of the CaMV 35S promoter (35S::YFP-bZIP60 S with mutated NLSs). 3-week-old *N*. *benthamiana* leaves were co-transformed via agroinfiltration to express these YFP-fusion proteins as well the nucleus indicator (35S::NLS-CFP) and a ER marker KDEL-MCherry [40]. Two days after transformation, their subcellular localizations were observed under the confocal microscope using a sequential scanning model. Three band-pass (BP) filters (BP 465–520 nm, BP 565–585 nm, and BP 590–630 nm) were used for CFP, YFP and MCherry signal collection, which were excited at 458 nm, 514 nm, and 543 nm respectively.

### Y2H Assay

The entry vectors with cDNAs of *bZIP60* S and *bZIP60ΔN* S were recombined into pGBKT7-GW (bait) and pGADT7-GW (prey) vectors using the LR reaction (Invitrogen, USA). Sets of constructs were co-transformed into Y2H Gold yeast strain (Clontech). The AD-T and BD-53 combination was utilized as positive control, whereas the AD-T and BD-Lam set as well as the empty pGBKT7 (BD) and pGADT7 (AD) were used as negative controls. Yeast transformants were selected on synthetic minimal double dropout medium deficient in TRP and LEU (DDO). Protein interactions were assessed on quadruple dropout medium deficient in HIS, TRP, LEU and adenine (QDO) in the presence of different concentrations of aureobasidin A (AbA).

### FRET Assay

To test homodimerization of bZIP60 S or bZIP60ΔN S in living cells, the cDNAs of *bZIP60* S and *bZIP60ΔN* S were cloned into pEarlyGate101 and pEarlyGate102 to generate YFP- and CFP-fusion proteins, respectively (Invitrogen, USA). 3-week-old *N*. *benthamiana* leaves were co-transformed with the indicated sets of constructs. Two days after transformation, sensitized emission FRET was determined under an inverted confocal microscope (TCS SP2, Leica, Germany). Images in donor (excitation 458 nm; emission 465 to 505 nm), acceptor (excitation 514 nm; emission 525 to 600 nm), and FRET (emission 525 to 600 nm) channels were captured. For acceptor photo-bleaching FRET, the fluorescence of the CFP and YFP channels was scanned as for sensitized emission FRET before and after photo-bleaching. Bleaching of the acceptor fluorescence signal was performed using a 514-nm beam at maximum intensity for 10 frames. The energy transfer efficiency between the paired proteins was quantified according to the change in fluorescence intensity of the acceptor and the donor before and after photo-bleaching.

### Complementation Test in *Arabidopsis*

To create complementation constructs, we cloned the *bZIP60* promoter containing a 3356 bp region immediately upstream of the ATG into the pMDC43 Gateway vector-substituting 2 × 35 S promoter to generate pMDC43-*pbZIP60* destination vector using bZIP60HindIII-F and bZIP60KpnI-R primers (S1 Table). The entry vectors bearing the cDNAs of *bZIP60* S and *bZIP60ΔN* S were recombined into the pMDC43-*pbZIP60* destination vector via the LR reaction, to generate the vectors *pbZIP60*-*bZIP60* S and *pbZIP60*-*bZIP60ΔN* S. The resulting constructs were introduced into GV3101 by electroporation. The *bzip60-2* mutant and *ire1a-3 ire1b-4* double mutant plants were transformed by the floral-dip method [83], and transformants were selected on solid half-strength MS medium supplemented with hygromycin (20 μg/mL) and confirmed by RT-PCR. The resulting homozygous transgenic lines (T2 generation) were used for phenotypic analyses.

For complementation assays by *IRE1A* or *IRE1B*, transgenic lines were made previously [28].

### Complementation Test in Yeast

To observe the subcellular localization of HAC1p and bZIP60 and to test their functional complementation, the relevant entry vectors were recombined via the LR reaction with a Gateway destination vector pAG423GAL-EYFP-ccdB (Plasmid 14341, Addgene) or a CEN-ARS Gateway destination vector pAG416GAL-ccdB-HA (Plasmid 14243, Addgene). The resulting destination vectors and the empty vectors were transferred into the CRY1 *Δhac1*::TRP strains with the Quick & Easy Yeast Transformation Mix (631851, Clontech). The CRY1 *Δhac1*::TRP yeast cells transformed with appropriate vectors were grown at 28°C in synthetic media lacking TRP and HIS and containing 2% raffinose (2 x S_Raf_-TRP-HIS). At exponential growth, the yeast cultures were spun down, washed and re-suspended to an OD_600_ = 0.3 with synthetic media lacking TRP and HIS and containing 2% galactose (2 x S_Gal_-TRP-HIS) to induce the expression of the fusion constructs at 28°C. After 8 h induction, the yeast cells were then incubated with 2 μg/mL DAPI for 1 h and processed for microscopy to visualize nuclei. DAPI signal were visualized with excitation at 405 nm and emission at 450–500 nm, and YFP signal was captured in another detection channel using a 514 nm excitation light and a 525–550 nm band-pass filter.

For functional complementation assays, the CRY1 *Δhac1*::TRP cells transformed with appropriate vectors were grown overnight to mid-log phase at 28°C in synthetic media lacking TRP and URA containing 2% glucose (2 x S_Glu_-TRP-URA). The yeast cells were then spun down, washed, and cultured in 2 x S_Raf_-TRP-URA media for 8 h to relief the glucose repression of Gal1 promoter. The cells were pelleted, washed and re-suspended to an OD_600_ = 0.3 in 2 x S_Gal_-TRP-URA media or in 2 x S_Raf_-TRP-URA media (as controls) to induce the expression of HAC1p and bZIP60. At 5 h and 10 h post-induction, the cell density was measured by a spectrophotometer (SmartSpec^Tm^ plus, Bio-Rad) to determine yeast growth. At 10 h post-induction, the induced and non-induced yeast cells were pelleted, washed and diluted to an OD_600_ = 1.0 with sterile water. 5-fold serial dilutions of the cells were spotted on the 2 x S_Gal_-TRP-URA plates in the presence of 0.1% DMSO or 0.2 μg/mL Tm and incubated for 48 h at 30°C. The non-induced cells were also spotted on 2 x S_Raf_-TRP-URA plates in the presence of 0.1% DMSO.

### Sequence Analyses

Multiple sequence alignment was generated by ClustalW [84]. Domain and NLS motif were predicted using SMART (http://smart.embl-heidelberg.de/) and NucPred (http://www.sbc.su.se/~maccallr/nucpred/), respectively. Figures were created by SigmaPlot 12.5.

### Accession Numbers

Gene sequences used in this study can be retrieved under the following accession numbers: AT1G42990 (*bZIP60*), AT2G40950 (*bZIP17*), AT3G10800 (*bZIP28*), AT2G17520 (*IRE1A*), AT5G24360 (*IRE1B*), AT5G42020 (*BiP2*), AT1G09080 (*BiP3*), AT1G21750 (*PDI*), AT1G56340 (*CRT*), AT3G18780 (*Actin II*), AB281271 (*NtbZIP60*), AJ236016 (*Nt18s RNA*), D26506 (*HAC1*), NM_001179209 (*IRE1*) and EF028235 (TuMV).

## Supporting Information

S1 Fig Confirmation of TuMV-induced spliced form of *bZIP60* by colony diagnostics and sequencing. **(A)** Specific primers overlap the exon/23-bp intron boundary to specifically detect *bZIP60* U (top) or the exon/exon boundary to specifically detect *bZIP60* S (bottom). Other annotations could be found in the legend of Fig 4A. **(B)** The extracts from the two bands showed in Fig 1A were cloned into T Easy Vector. A total of 10 colonies selected at random were tested by diagnostic PCR. The primer sets specific for *bZIP60* S could amplify products with right size in all colonies, whereas the primer sets specific for *bZIP60* U not, indicating that the selected colonies do not contain the 23-bp sequence. T Easy Vector, cDNA and genomic DNA were also PCR analyzed as controls. **(C)** Other three colonies at each time point were selected for forward (top) and reverse (bottom) sequencing. Note that the 23-bp intron marked with a box is absent in all selected six colonies (just sequences from two colonies shown here).
(TIF)

S2 Fig Induction of the UPR by TuMV infection. **(A)** The visible symptoms of the wild type at 12 d after infiltrated without (Blank) or with buffer, GV3101, or GV3101 containing TuMV infectious plasmids at OD_600_ = 0.2. **(B)** ER stress marker gene *BiP3* is up-regulated in response to TuMV challenge. RNA extracted at three time points from the local leaves after the indicated treatment was used for semi-quantitative RT-PCR. *CP* and *Actin II* were also analyzed to see the virus accumulation and to sever as a loading control, respectively. The size of PCR products were indicated at right. **(C)** and **(D)** The mRNA level of TuMV *CP* was determined in local **(C)** and systemically **(D)** infected leaves at the indicated time points by qRT-PCR. RNA from local leaves used for qRT-PCR is described in **(A)**. Only systemically infected leaves under GV3101 or TuMV challenge were used to extract RNA for qRT-PCR at two time points. *Actin II* was used as an internal control for qRT-PCR. Data represent means with SD of three biological replicates. **(E)** and **(F)** ER stress marker genes are specifically up-regulated in response to TuMV attack in local **(E)** and systemically **(F)** infected leaves. RNA from local and systemically infected leaves used for qRT-PCR analysis is described in **(A)** and **(D)**, respectively. *Actin II* was used as an internal control for qRT-PCR. Data represent means with SD of three biological replicates.
(TIF)

S3 Fig Transient expression of each TuMV proteins in *N*. *benthamiana*. **(A)** A schematic represent of Gateway constructs used for creating fusion proteins GFP-viral factors (top) and viral factors-GFP (bottom). **(B)** At 2.5 dpi, the leaves with the indicated agroinfiltration were subjected to confocal to visualize the transient expression. Only images showing the expression of GFP-viral factors were presented. Bars = 20 μm.
(TIF)

S4 Fig Predicted structure of *NtbZIP60* mRNA. Lowest free energy form (*ΔG* = -349.52 [initially -78.60]) of *NtbZIP60* mRNA folded by M-Fold. Open red boxed area is magnified in detail in Fig 2A. The inserted panel showed that among 19 forms of *NtbZIP60* with different free energy, 17 forms (89.5%) could fold into twin hairpin loop.
(TIF)

S5 Fig Multiple mRNA sequence alignment of *bZIP60* homologues in plants. Two conserved regions of these mRNA were found, which correspond to the NLS/ZIP and TMD domains in protein level (see S11 Fig). The identical nucleotides are highlighted with red. The predicted intron to be removed and splicing sites were indicated by a green box and scissors, respectively. The predicted intron is involved in encoding TMD in all selected plants. Note that the sequence for forming twin kissing loop and the nucleotides (indicated by asterisks) important for splicing are extremely conserved in all selected plants. The number after mRNA names represents the mRNA length, and omitted nucleotides for these homologues showed no similarity. Other information of these sequences is presented in detail in S2 Table.
(TIF)

S6 Fig T-DNA insertion disrupts the genomic DNA structure in the *bzip60-1* and *bzip60-2* mutants. The sequence of the genomic DNA amplification products from *bzip60-1* (**A**) and *bzip60-2* (**B**) mutants using the primers indicated by arrows was aligned with the wild type *bZIP60* DNA. Note that the gray-shaded regions represent the consistent sequences, without showing the indicated nt. The sequences marked in red represent the part of T-DNA sequences inserted into *bZIP60* genome in the two mutants. The 23 nt shaded in yellow represent the target of unconventional splicing. A red triangle indicates that the positions of T-DNA insertion in the *bZIP60* genomic DNA are at 41 and 1116 in the *bzip60-1* and *bzip60-2* mutants relative to the first ATG, respectively. **(A)** The red and blue arrows indicate the start codons of two in-frame ORFs (bZIP60 and bZIP60ΔN), respectively. The nucleotides in an open box represent the changeable site used for T-DNA insertion. Colonies—(8/8) and Colonies_ (3/3) indicated that the sequencing carried out on 8 **(A)** and 3 **(B)** selected colonies generated the same result.
(TIF)

S7 Fig The effects of T-DNA insertion on *bZIP60* mRNA in the *bzip60-1* and *bzip60-2* mutants. The sequence of the cDNA amplification products from the *bzip60-1* mutant **(A)** treated by DMSO, Tm, and infected with TuMV as well as from the *bzip60-2* mutant **(B)** was aligned with the wild type *bZIP60* cDNA. The gray-shaded regions represent the sequences that are consistent with wild type bZIP60 cDNA. The red triangles indicated the corresponding position of T-DNA insertion in cDNAs. **(A)** The two start codons are shaded in green. bZIP60-1218 R is a bZIP60 specific primer, and AAP is a universal primer provided by 5′ RACE kit (see S1 Text). The nt shaded in red are the part of T-DNA shown in S6 Fig. The 23 nt shaded in yellow are removed in TuMV-infected plants. Note that T-DNA insertion disrupts the *bZIP60*, not the *bZIP60ΔN*, and that the *bZIP60ΔN* possess different 5’ ends. n, the number of selected colonies for sequencing. **(B)** bZIP60 S7 and LB2 were used for amplifying 3′ end of bZIP60 in the *bzip60-2* mutant. An in-frame stop codon (Stop ΔC) was introduced due to the T-DNA insertion, generating *bZIP60ΔC2*, indicated by a blue hand-arrow. Note that *bZIP60ΔC1* without T-DNA tail was also analyzed in S14 Fig. Stop S and Stop U represent the stop codon for bZIP60 S and bZIP60 U, respectively. Colonies_ (3/3) indicated that the sequencing carried out on three selected colonies generated the same result.
(TIF)

S8 Fig Mapping of the domains of yeast HAC1p and *Arabidopsis* bZIP60. **(A)** Amino acid sequence alignment of bZIP60, *bZIP60ΔN* and *bZIP60ΔC* was shown. Identical sequences are shaded in gray. Due to the frame-shift mediated by the removal of 23 nt from *bZIP60* U mRNA, the C-terminus of bZIP60 S is different from that of bZIP60 U, and thus lose the TMD (in purple) of bZIP60 U. The amino acids 138–197 indicated by a line above sequence are defined as bZIP domain according to the prediction by SMART and the previous report [15,22]. The amino acids in red containing a NLS consensus motif (K/RR/KxR/K) [45] are therefore predicted as NSLs of bZIP60 or its derivatives. The sequence (aa 41–81 in blue) was previously described as an AD [49]. Note that a truncated bZIP60 without the C-terminus (aa 218–258), indicated by a hand-arrow, has not been found to homodimerize [23]. The NLS/BD and the AD between HAC1p and bZIP60 are compared in detail in Figs 9A and 10A, respectively. **(B)** Amino acid sequence alignment of HAC1p U and HAC1p S was shown. Identical sequences are shaded in gray. Due to the removal of 252 nt from *HAC1* mRNA via unconventional splicing, HAC1p S gains an AD (aa 221–238, in blue) [18]. The amino acids 37–95 in both HAC1p U and HAC1p S are defined as bZIP (BD) according to the prediction by SMART and the previous description [48]. The sequence shaded with red is the NLS of HAC1p U and HAC1p S [48].
(TIF)

S9 Fig Detecting *bZIP60* S and *bZIP60* U with or without stress. **(A)** and **(B)** qRT-PCR analysis of total *bZIP60* transcripts **(A)** and *bZIP60* S **(B)** level in unopened and opened flowers from the wild type and the two *bzip60* mutants. The abundance of *bZIP60* and *bZIP60* S was normalized to that of *Actin II* transcripts. Data represent means with SD of three biological replicates. ** *P*<0.01, *** *P*<0.001, unpaired two-tailed Student’s test. ns, non-significant. **(C)** Detection of *bZIP60* U and *bZIP60* S in 3-week-old seedlings from the wild type, *bzip60-1* and *bzip60-2* mutants. Seedlings were treated with 2 mM DTT or 5 μg/mL Tm for 2 h in liquid MS medium (see S1 Text). 0.1% DMSO was used as a vehicle control. *Actin II* served as a loading control. The number represents the biological replicates. Note that both unspliced and spliced *bZIP60* (*bZIP60ΔC*) were not detectable in *the bzip60-2* mutant. *bZIP60* U (*bZIP60ΔN* U for *bzip60-1* mutant) could be detected in the wild type and *bzip60-1* mutant regardless of stress treatment or not. Lower level of *bZIP60* S (*bZIP60ΔN* S) was found in the *bzip60-1* mutant under DTT or Tm treatment, compared to the wild type.
(TIF)

S10 Fig *bZIP60* and *bZIP60ΔN* fold into twin hairpin loops. **(A)** Both *bZIP60* and *bZIP60ΔN* folds into kissing hairpin loop containing two splicing sites indicated by scissors. Each loop contains three conserved amino acids (red). **(B)** The percentage of predicted different free energy forms of *bZIP60* mRNAs with the twin kissing hairpin is compared between full length *bZIP60*, *bZIP60ΔN* without the first 44 nt and with a T-DNA sequence (TGTTATT) (-44 nt), and *bZIP60ΔN* without the first 150 nt (-150 nt). n indicates the total number of predicted *bZIP60* mRNAs structure.
(TIF)

S11 Fig Multiple protein sequence alignment of bZIP60 homolouges in plants. Two conserved regions of these proteins ware found on unspliced proteins **(A)**, one is NLS/ZIP and the other TMD that is absent in spliced proteins **(B)** (see S5 Fig). A schematic of the bZIP consensus is shown above by highlighting the extremely conserved residues [54]. The NSL consensus motifs are indicated by down arrows. Note that the slicing produces two NLS motifs in the new sequences of spliced proteins (NLS1 and NLS2). Other information of these proteins is presented in detail in S2 Table.
(TIF)

S12 Fig Homo-interaction of bZIP60 S and bZIP60ΔN S. **(A)** The strength of homo-interactions of bZIP60 S and bZIP60ΔN S was tested by a liquid selective QDO medium containing AbA. The transformed cells were cultured in DDO medium overnight, pelleted, washed and diluted to an OD_600_ = 0.5 by a selective QDO medium with different concentrations of AbA. Data represent means with SD of three biological replicates. Note that the homo-interaction of bZIP60 S and bZIP60ΔN S could no longer support yeast growth in the presence of 1000 ng/mL AbA, compared to controls (also see Fig 6A). **(B)** Homo-interactions of bZIP60 S and bZIP60ΔN S in living cells by sensitized emission FRET assay. Note that only cells co-expressing bZIP60 S-CFP and bZIP60 S-YFP or bZIP60ΔN S-CFP and bZIP60ΔN S-YFP exhibited FRET signal. Experiments were repeated three times with similar results. Bars = 50 μm.
(TIF)

S13 Fig Viral accumulation and symptoms in the single-gene mutants of UPR pathways. **(A)** Phenotypes of the wild type and *ire1b-4* mutant at 12 dpi after inoculation with buffer or TuMV. Note that although *ire1b-4* developed slower TuMV symptoms during a little early stage of virus infection (12 dpi), it eventually produced same viral symptoms at 21 dpi, observed in white light and ultraviolet (UV) lamp **(B)**, compared to the wild type. **(D)**, **(E)** and **(F)** Phenotypes of the wild type, two IRE1A mutants (*ire1a-3 and ire1a-2*), *bzip17*, *bzip28* and *bip2-2* at 18 dpi after inoculation with buffer or TuMV. Note that the mutant mutants all developed typical TuMV symptoms, compared to the wild type. **(C)** and **(G)** qRT-PCR analysis of TuMV *CP* in the wild type and the single mutants. At 12 dpi (for *ire1b-4*) (**C**) or 18 dpi (**G**) after inoculation with buffer or TuMV, RNA was extracted from the systemic leaves, and qRT-PCR was carried out. *Actin II* was used as an internal control for quantitative RT-PCR. Data represent means with SD of three biological replicates. * *P*<0.05, unpaired two-tailed Student’s test. ns, non-significant.
(TIF)

S14 Fig bZIP60 S with mutated NLSs or bZIP60ΔCs fail to complement HAC1p in yeast. *bZIP60* S with mutated NLS3 and NLS1 (see Figs 5 and 9), *bZIP60ΔC1* and *bZIP60ΔC2* (see S8 Fig) failed to inhibit yeast growth **(A)** and to rescue the ER-stress sensitive phenotype of CRY1 *Δhac1*::TRP strains **(B)**, compared to the untransformed cells and the transformed cells with *HAC1p* S. The assays were conducted according to the procedures presented in detail in Figs 10 and 11. **(A)** Data represent means with SD of three experiments. *** *P*<0.001, unpaired two-tailed Student’s test. ns, non-significant. **(B)** Experiments were repeated three times with similar results.
(TIF)

S15 Fig *Arabidopsis* IRE1A or IRE1B fails to complement IRE1p in yeast. Functional complementation was tested by confocal imaging of CRY1 *Δire1*::KanMX6 strains with an integrated pRS304 4 x UPRE-GFP reporter. The cells were transformed with CEN-ARS plasmids expressing yeast *IRE1*, *Arabidopsis IRE1A* or *IRE1B* under the control of a GPD promoter. The transformed yeasts were selected and grown in the 2 x SD medium deficient in TRP and URA with 250 μg/mL G418 (10131–035, Invitrogen) and 2% raffinose, using monosodium glutamate (G1626, Sigma) as nitrogen source. ER stress was induced by 2 mM DTT in the presence of 2% glucose. Experiments were repeated three times with similar results. Bars = 20 μm.
(TIF)

S16 Fig Pharmacological small molecular chaperones 4-PBA and TUDCA promote TuMV infection in *N*. *benthamiana*. *N*. *benthamiana* seedlings were pre-treated with 0.1% DMSO, 4-PBA (1 mM), TUDCA (1 mM) or 4-PBA (1 mM) plus TUDCA (1 mM) for 6 h (see S1 Text). The pre-treated leaves were then selected to be inoculated with TuMV. After 7 and 10 dpi, pictures of *N*. *benthamiana* seedlings were taken under UV light **(A)**, and RNA was extracted from the indicated leaves. **(B)** TuMV accumulation indicated by *CP* mRNA level was analyzed by qRT-PCR. Data represent means with SD of three experiments. *P<0.05, **P<0.01, ***P<0.001, unpaired two-tailed Student’s test, ns, non-significant.
(TIF)

S17 Fig TuMV 6K2 does not interact with IRE1A or IRE1B. The relevant entry vectors were cloned into the Gateway version of bimolecular fluorescence complementation (BiFC) vectors to fuse the split YN and YC at the C-termini of 6K2, IRE1A and IRE1B. The sets of constructs were subjected to the transient expression system, and YFP signal was captured under the confocal at excitation 514 nm and emission 525–600 nm. BiFC assay demonstrated the homo-interactions of TuMV 6K2, IRE1A and IRE1B, but no interaction of TuMV 6K2 with IRE1A or IRE1B. Experiments were repeated three times with similar results. Bars = 50 μm.
(TIF)

S18 Fig Root phenotype of the wild type, *bzip60-1* and *bzip60-2* mutants. **(A)** The wild type, *bzip60-1* and *bzip60-2* mutants were grown on half-strength MS medium for 9 d after germination. **(B)** Root lengths were measured in 9-d-old seedlings. Box plots represent the value range and the variability of root lengths. The boundaries of each box represent the lower 25^th^ and upper 75^th^ percentiles, and the horizontal line within the box represents the median value. The spacing within the box indicates the degree of dispersal in the data. The lines at the top and bottom of the box (whiskers) represent the minimum and maximum. Outliers are indicated by solid circles. Statistical analysis was conducted and showed no difference in root length among wild type and mutants.
(TIF)

S1 Table Primers Used in this Study. (DOC)

S2 Table *Arabidopsis bZIP60* Homologues Analyzed in this Study. (DOC)

S1 Text Stress Treatment by Tm and DTT, 5′ Rapid Amplification of cDNA Ends (5′ RACE), and Pharmacological Molecular Chaperones Treatment. (DOC)
