# Supplementary figures and images for: The UPR Branch IRE1-bZIP60 in Plants Plays an Essential Role in Viral Infection and Is Complementary to the Only UPR Pathway in Yeast
Source: PLoS Genet. 2015 Apr 15;11(4):e1005164. doi: 10.1371/journal.pgen.1005164 (PMC4398384; doi:10.1371/journal.pgen.1005164)

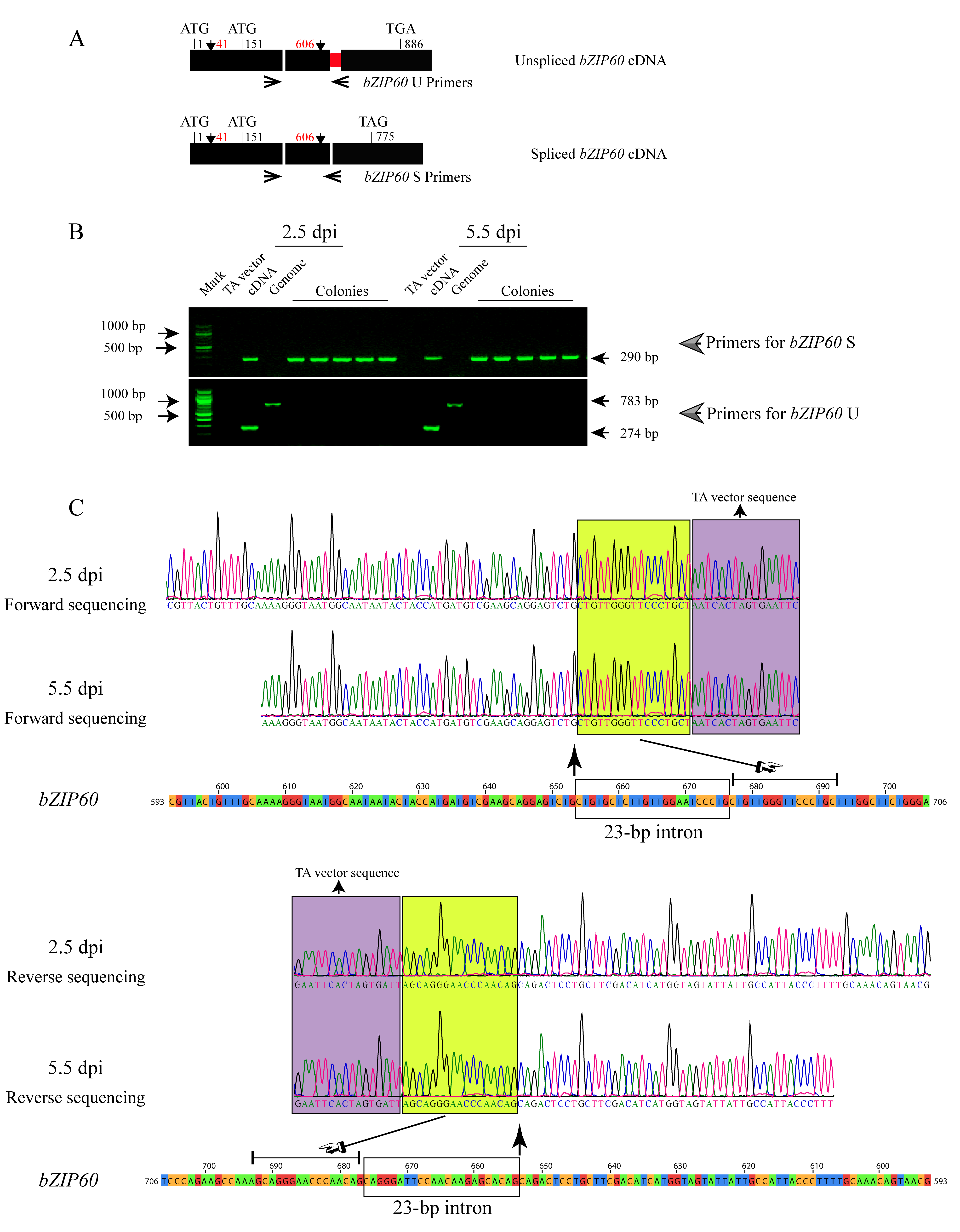

Supplement: S1 Fig — (A) Specific primers overlap the exon/23-bp intron boundary to specifically detect bZIP60 U (top) or the exon/exon boundary to specifically detect bZIP60 S (bottom). Other annotations could be found in the legend of Fig 4A. (B) The extracts from the two bands showed in Fig 1A were cloned into T Easy Vector. A total of 10 colonies selected at random were tested by diagnostic PCR. The primer sets specific for bZIP60 S could amplify products with right size in all colonies, whereas the primer sets specific for bZIP60 U not, indicating that the selected colonies do not contain the 23-bp sequence. T Easy Vector, cDNA and genomic DNA were also PCR analyzed as controls. (C) Other three colonies at each time point were selected for forward (top) and reverse (bottom) sequencing. Note that the 23-bp intron marked with a box is absent in all selected six colonies (just sequences from two colonies shown here). (TIF) [file pgen.1005164.s001.tif]

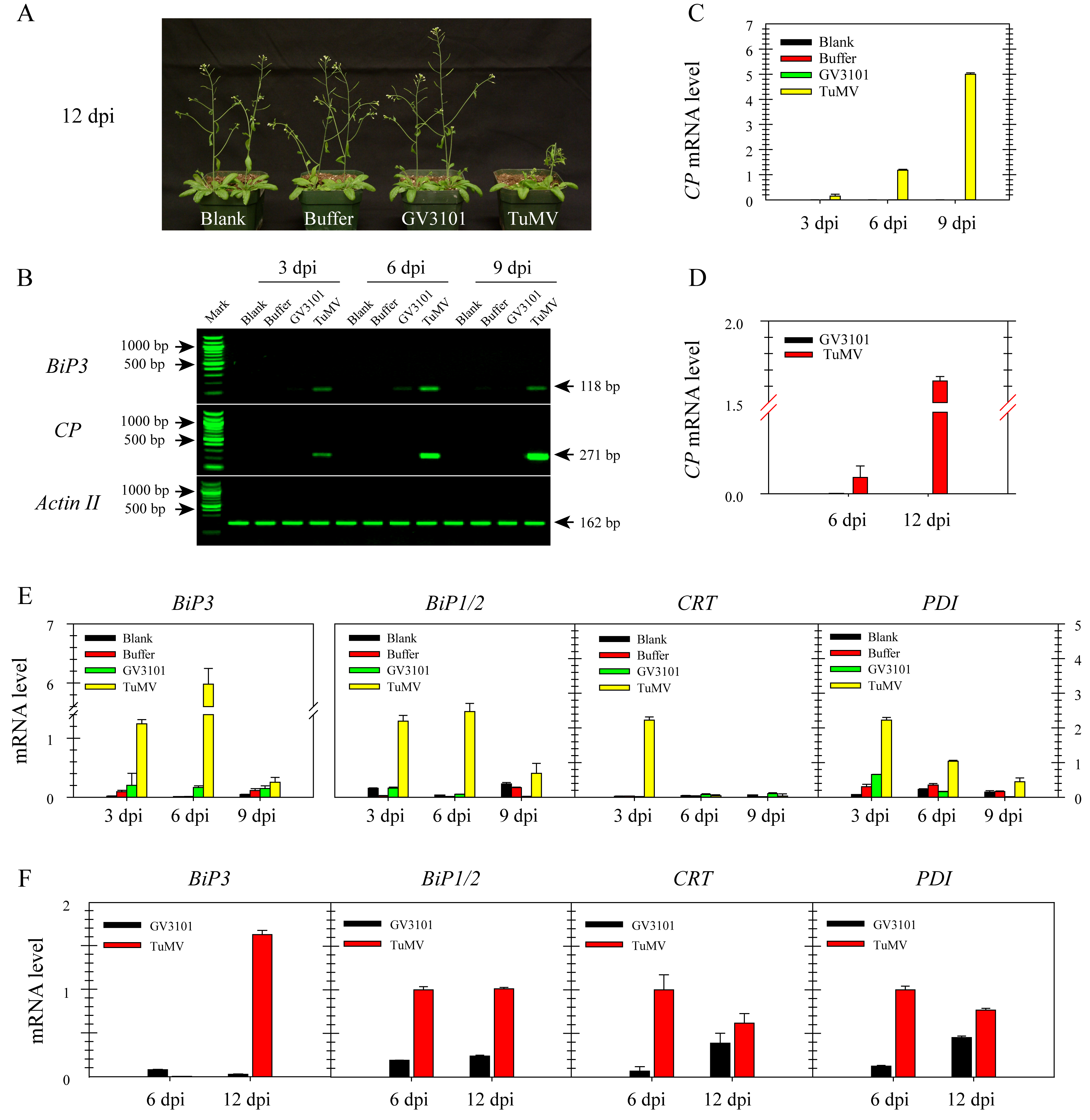

Supplement: S2 Fig — (A) The visible symptoms of the wild type at 12 d after infiltrated without (Blank) or with buffer, GV3101, or GV3101 containing TuMV infectious plasmids at OD600 = 0.2. (B) ER stress marker gene BiP3 is up-regulated in response to TuMV challenge. RNA extracted at three time points from the local leaves after the indicated treatment was used for semi-quantitative RT-PCR. CP and Actin II were also analyzed to see the virus accumulation and to sever as a loading control, respectively. The size of PCR products were indicated at right. (C) and (D) The mRNA level of TuMV CP was determined in local (C) and systemically (D) infected leaves at the indicated time points by qRT-PCR. RNA from local leaves used for qRT-PCR is described in (A). Only systemically infected leaves under GV3101 or TuMV challenge were used to extract RNA for qRT-PCR at two time points. Actin II was used as an internal control for qRT-PCR. Data represent means with SD of three biological replicates. (E) and (F) ER stress marker genes are specifically up-regulated in response to TuMV attack in local (E) and systemically (F) infected leaves. RNA from local and systemically infected leaves used for qRT-PCR analysis is described in (A) and (D), respectively. Actin II was used as an internal control for qRT-PCR. Data represent means with SD of three biological replicates. (TIF) [file pgen.1005164.s002.tif]

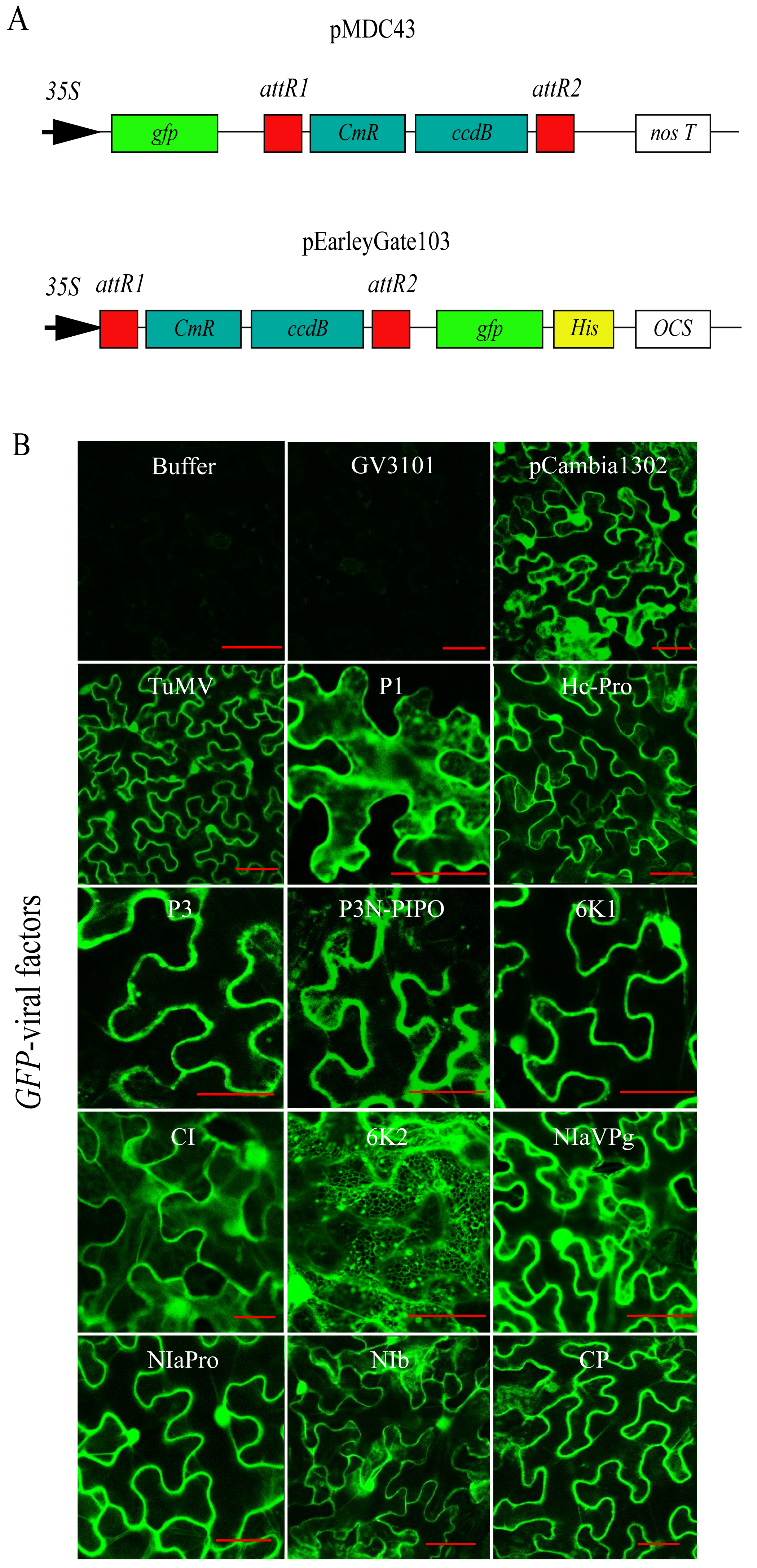

Supplement: S3 Fig — (A) A schematic represent of Gateway constructs used for creating fusion proteins GFP-viral factors (top) and viral factors-GFP (bottom). (B) At 2.5 dpi, the leaves with the indicated agroinfiltration were subjected to confocal to visualize the transient expression. Only images showing the expression of GFP-viral factors were presented. Bars = 20 μm. (TIF) [file pgen.1005164.s003.tif]

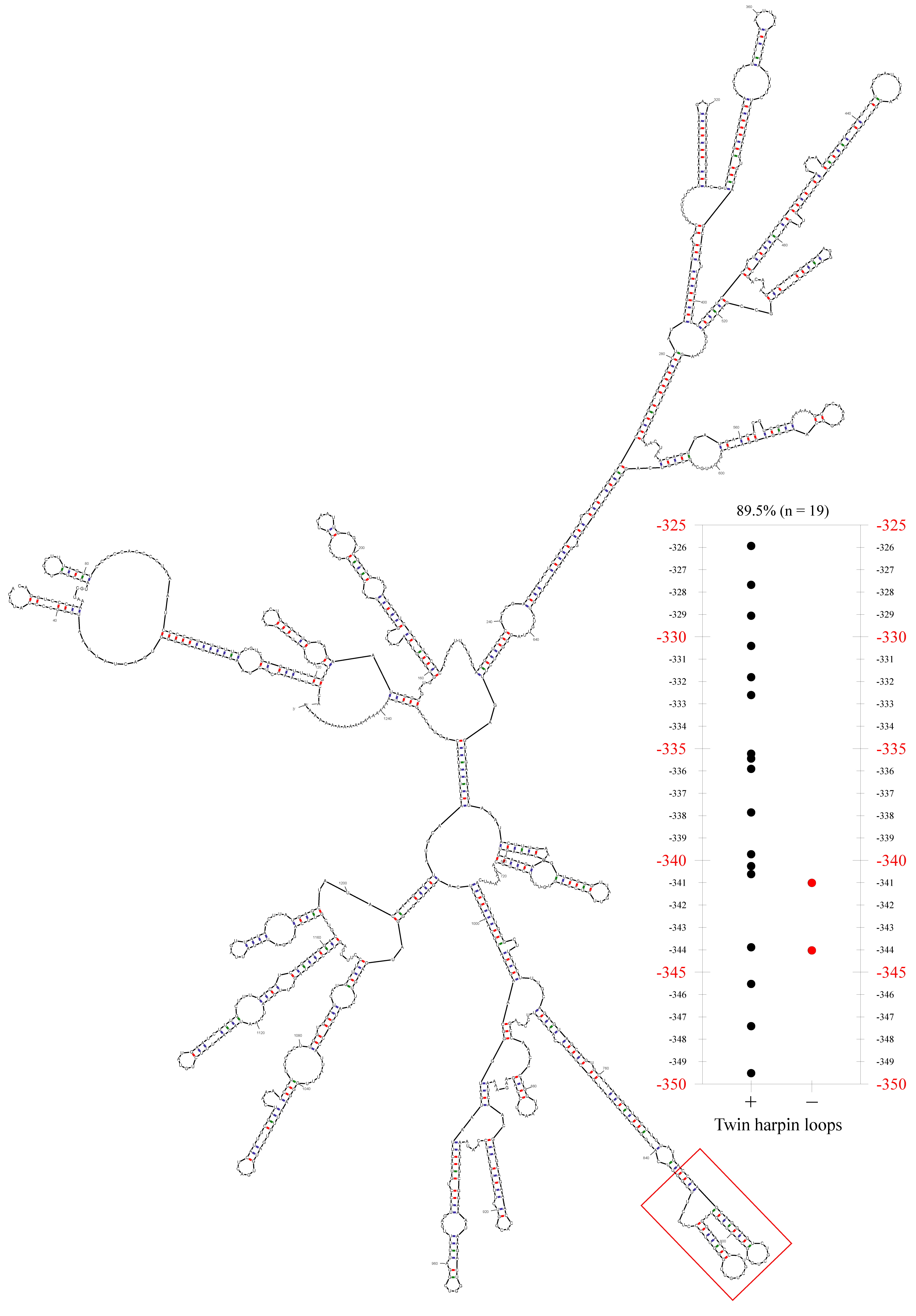

Supplement: S4 Fig — Lowest free energy form (ΔG = -349.52 [initially -78.60]) of NtbZIP60 mRNA folded by M-Fold. Open red boxed area is magnified in detail in Fig 2A. The inserted panel showed that among 19 forms of NtbZIP60 with different free energy, 17 forms (89.5%) could fold into twin hairpin loop. (TIF) [file pgen.1005164.s004.tif]

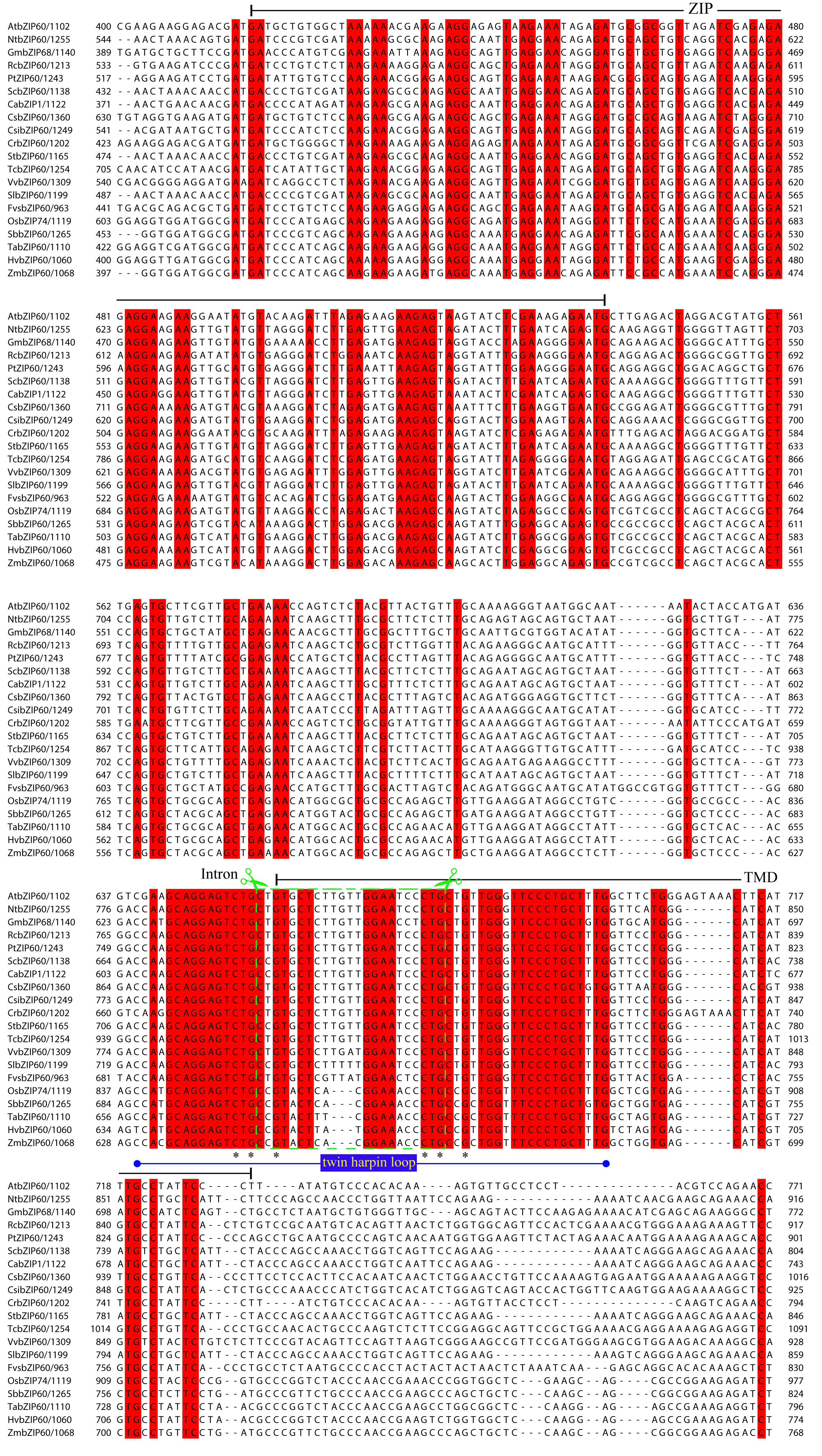

Supplement: S5 Fig — Two conserved regions of these mRNA were found, which correspond to the NLS/ZIP and TMD domains in protein level (see S11 Fig). The identical nucleotides are highlighted with red. The predicted intron to be removed and splicing sites were indicated by a green box and scissors, respectively. The predicted intron is involved in encoding TMD in all selected plants. Note that the sequence for forming twin kissing loop and the nucleotides (indicated by asterisks) important for splicing are extremely conserved in all selected plants. The number after mRNA names represents the mRNA length, and omitted nucleotides for these homologues showed no similarity. Other information of these sequences is presented in detail in S2 Table. (TIF) [file pgen.1005164.s005.tif]

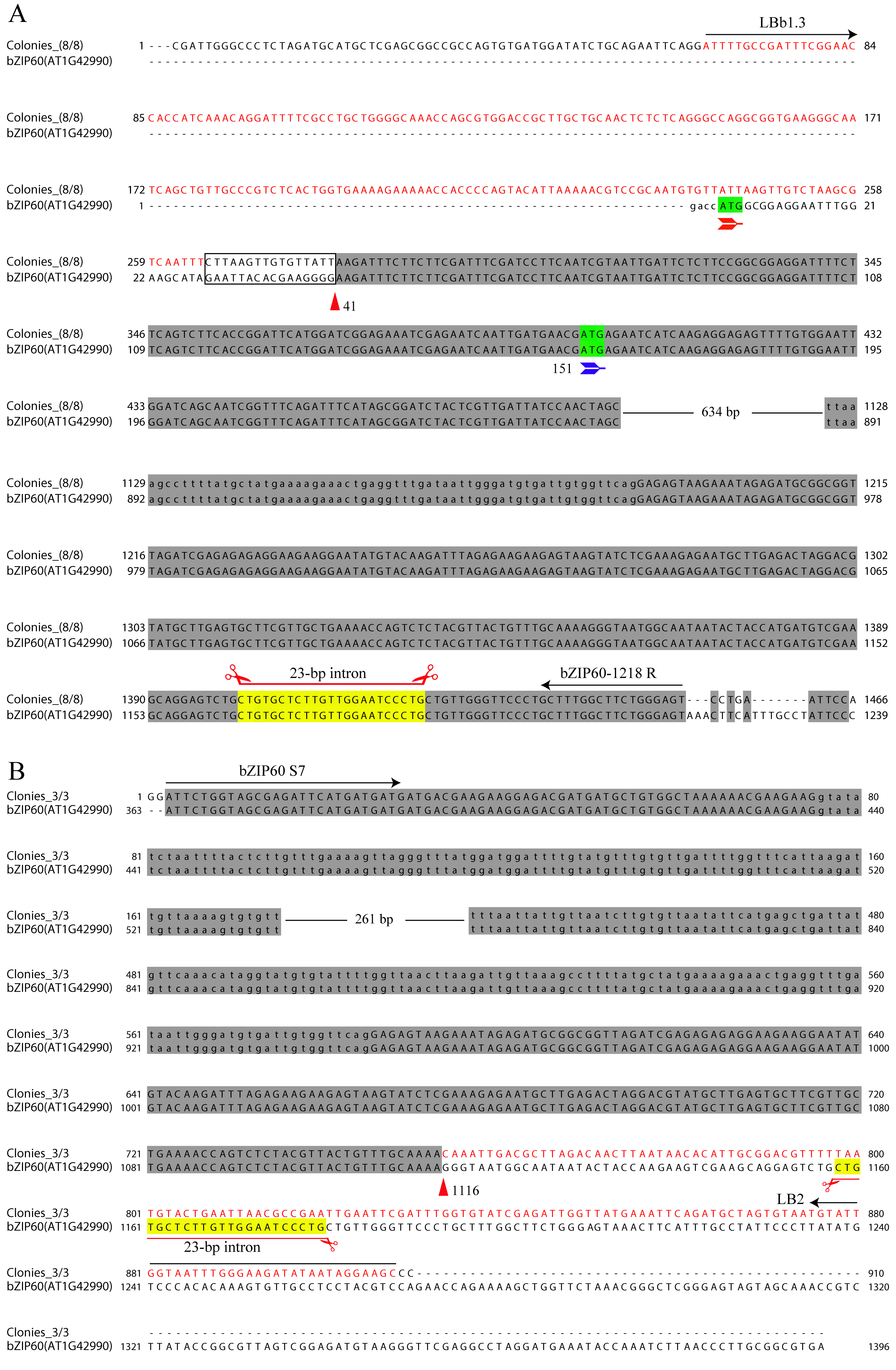

Supplement: S6 Fig — The sequence of the genomic DNA amplification products from bzip60-1 (A) and bzip60-2 (B) mutants using the primers indicated by arrows was aligned with the wild type bZIP60 DNA. Note that the gray-shaded regions represent the consistent sequences, without showing the indicated nt. The sequences marked in red represent the part of T-DNA sequences inserted into bZIP60 genome in the two mutants. The 23 nt shaded in yellow represent the target of unconventional splicing. A red triangle indicates that the positions of T-DNA insertion in the bZIP60 genomic DNA are at 41 and 1116 in the bzip60-1 and bzip60-2 mutants relative to the first ATG, respectively. (A) The red and blue arrows indicate the start codons of two in-frame ORFs (bZIP60 and bZIP60ΔN), respectively. The nucleotides in an open box represent the changeable site used for T-DNA insertion. Colonies—(8/8) and Colonies_ (3/3) indicated that the sequencing carried out on 8 (A) and 3 (B) selected colonies generated the same result. (TIF) [file pgen.1005164.s006.tif]

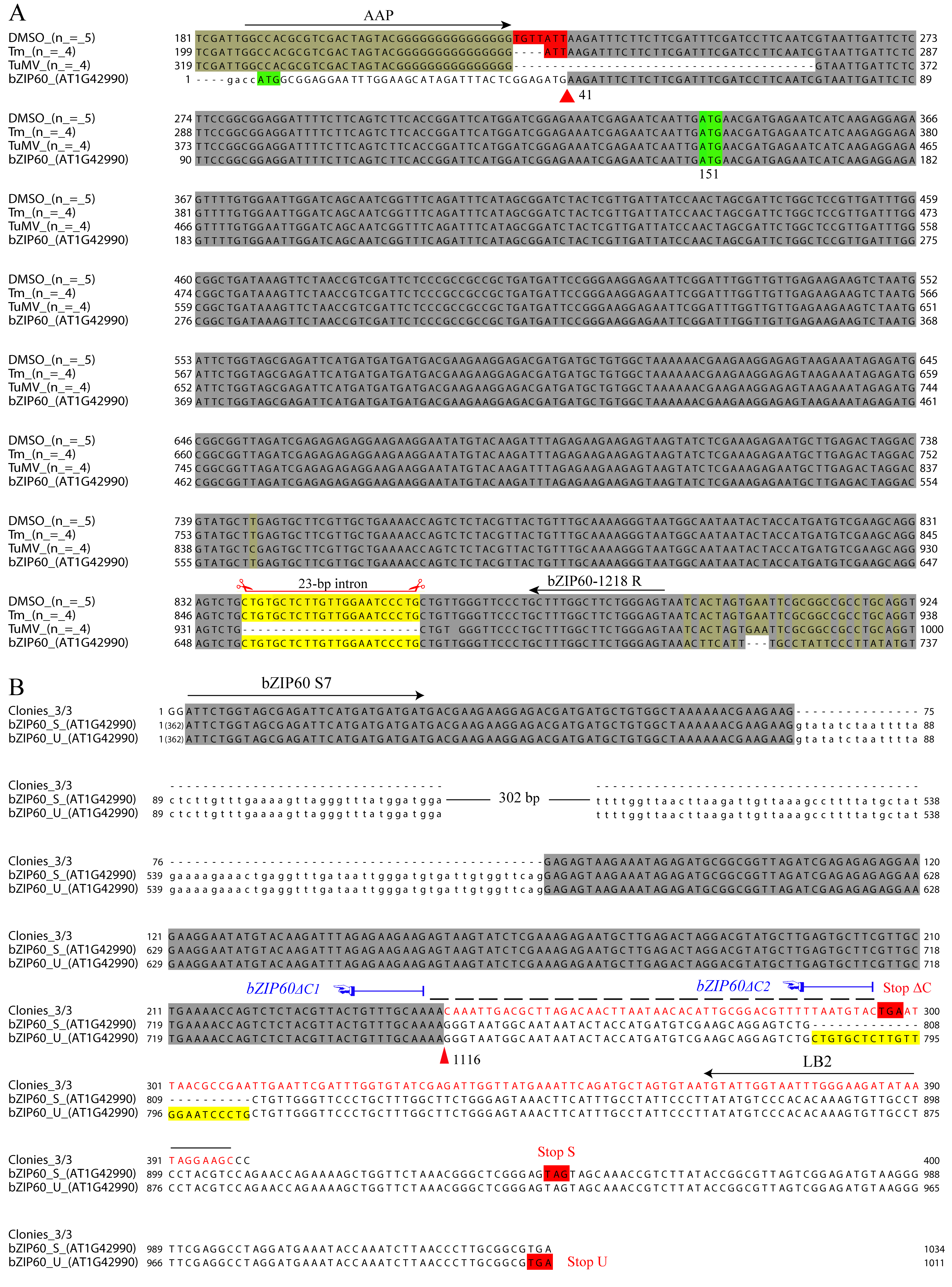

Supplement: S7 Fig — The sequence of the cDNA amplification products from the bzip60-1 mutant (A) treated by DMSO, Tm, and infected with TuMV as well as from the bzip60-2 mutant (B) was aligned with the wild type bZIP60 cDNA. The gray-shaded regions represent the sequences that are consistent with wild type bZIP60 cDNA. The red triangles indicated the corresponding position of T-DNA insertion in cDNAs. (A) The two start codons are shaded in green. bZIP60-1218 R is a bZIP60 specific primer, and AAP is a universal primer provided by 5′ RACE kit (see S1 Text). The nt shaded in red are the part of T-DNA shown in S6 Fig. The 23 nt shaded in yellow are removed in TuMV-infected plants. Note that T-DNA insertion disrupts the bZIP60, not the bZIP60ΔN, and that the bZIP60ΔN possess different 5’ ends. n, the number of selected colonies for sequencing. (B) bZIP60 S7 and LB2 were used for amplifying 3′ end of bZIP60 in the bzip60-2 mutant. An in-frame stop codon (Stop ΔC) was introduced due to the T-DNA insertion, generating bZIP60ΔC2, indicated by a blue hand-arrow. Note that bZIP60ΔC1 without T-DNA tail was also analyzed in S14 Fig. Stop S and Stop U represent the stop codon for bZIP60 S and bZIP60 U, respectively. Colonies_ (3/3) indicated that the sequencing carried out on three selected colonies generated the same result. (TIF) [file pgen.1005164.s007.tif]

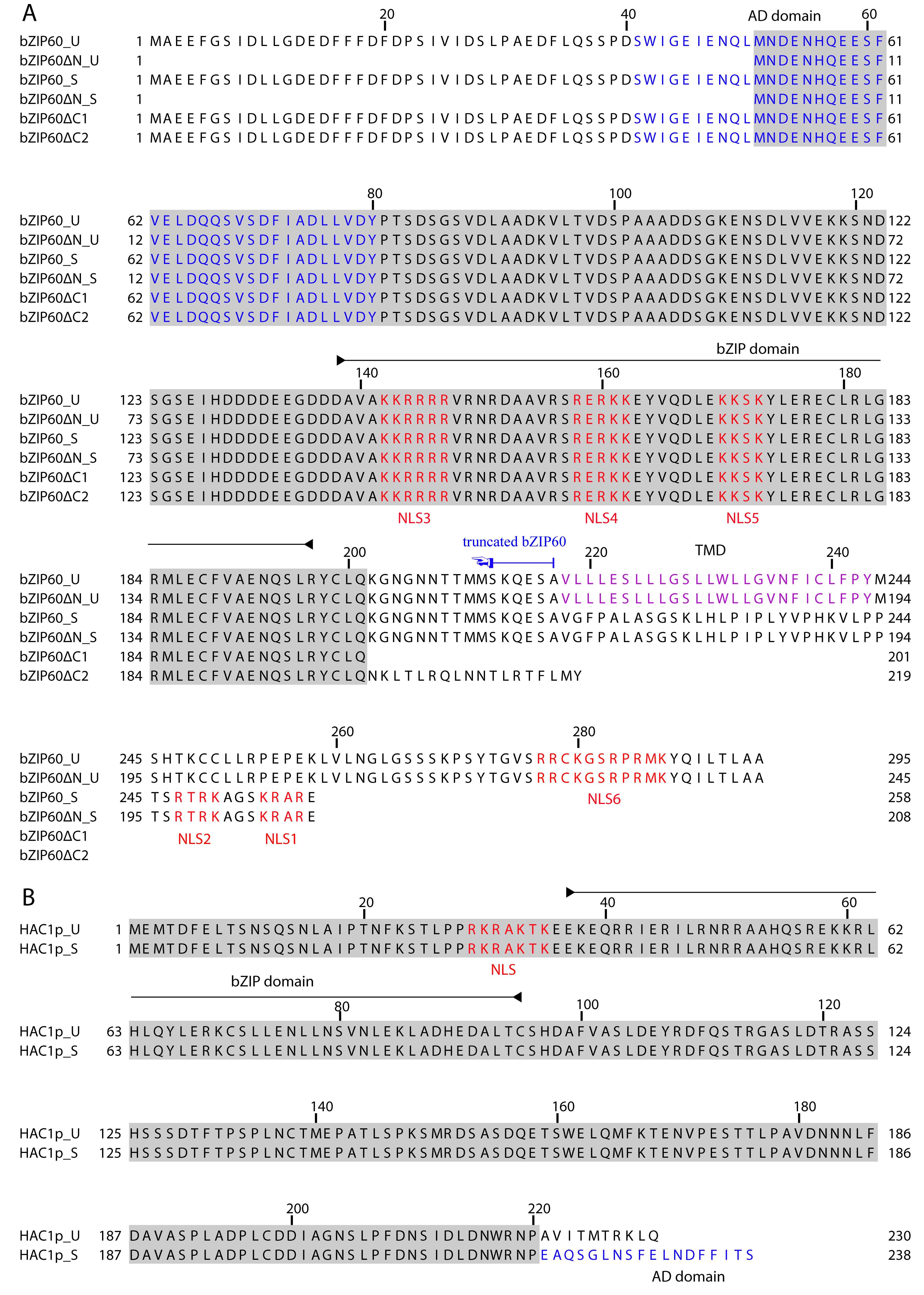

Supplement: S8 Fig — (A) Amino acid sequence alignment of bZIP60, bZIP60ΔN and bZIP60ΔC was shown. Identical sequences are shaded in gray. Due to the frame-shift mediated by the removal of 23 nt from bZIP60 U mRNA, the C-terminus of bZIP60 S is different from that of bZIP60 U, and thus lose the TMD (in purple) of bZIP60 U. The amino acids 138–197 indicated by a line above sequence are defined as bZIP domain according to the prediction by SMART and the previous report [15,22]. The amino acids in red containing a NLS consensus motif (K/RR/KxR/K) [45] are therefore predicted as NSLs of bZIP60 or its derivatives. The sequence (aa 41–81 in blue) was previously described as an AD [49]. Note that a truncated bZIP60 without the C-terminus (aa 218–258), indicated by a hand-arrow, has not been found to homodimerize [23]. The NLS/BD and the AD between HAC1p and bZIP60 are compared in detail in Figs 9A and 10A, respectively. (B) Amino acid sequence alignment of HAC1p U and HAC1p S was shown. Identical sequences are shaded in gray. Due to the removal of 252 nt from HAC1 mRNA via unconventional splicing, HAC1p S gains an AD (aa 221–238, in blue) [18]. The amino acids 37–95 in both HAC1p U and HAC1p S are defined as bZIP (BD) according to the prediction by SMART and the previous description [48]. The sequence shaded with red is the NLS of HAC1p U and HAC1p S [48]. (TIF) [file pgen.1005164.s008.tif]

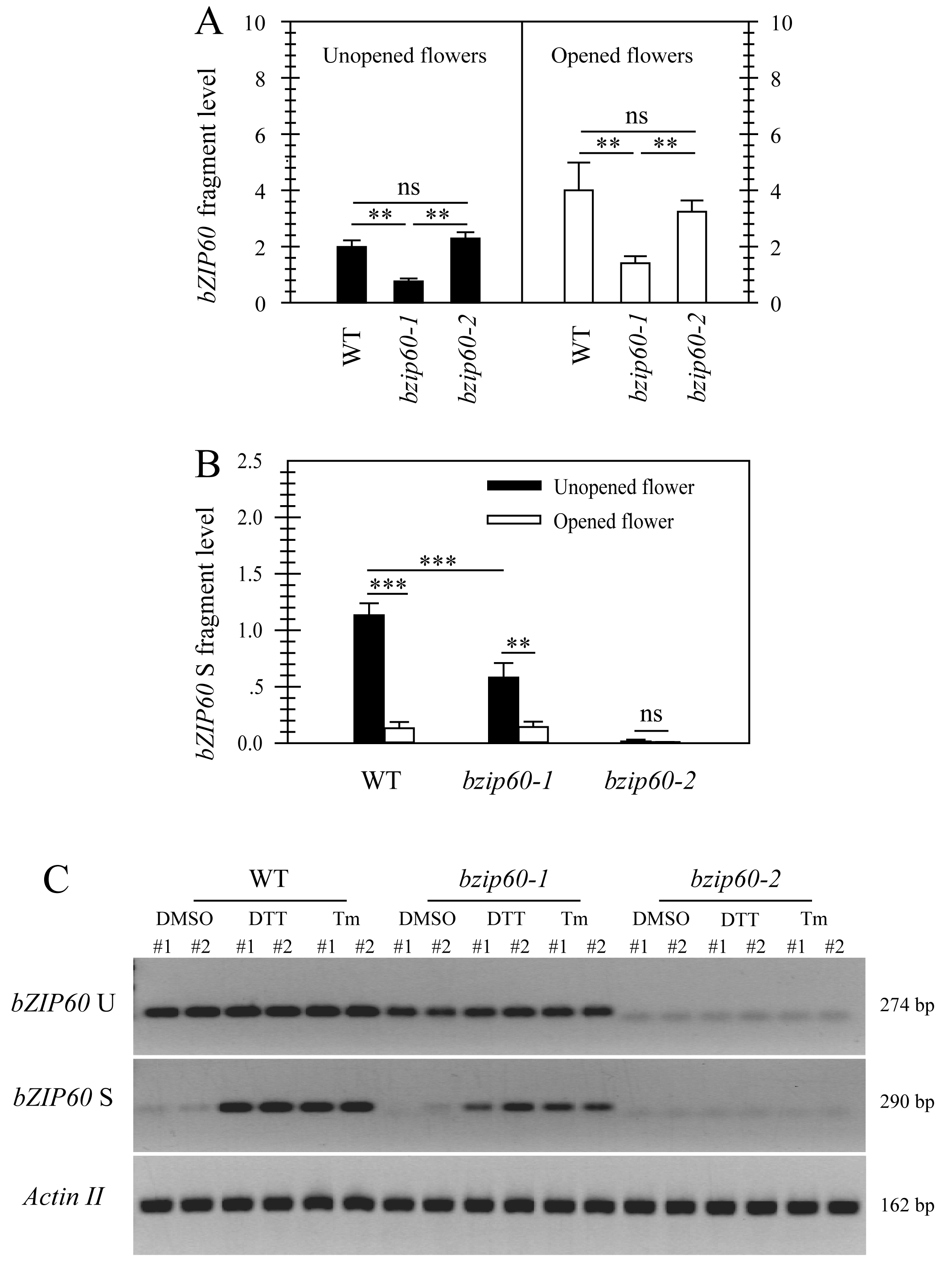

Supplement: S9 Fig — (A) and (B) qRT-PCR analysis of total bZIP60 transcripts (A) and bZIP60 S (B) level in unopened and opened flowers from the wild type and the two bzip60 mutants. The abundance of bZIP60 and bZIP60 S was normalized to that of Actin II transcripts. Data represent means with SD of three biological replicates. ** P<0.01, *** P<0.001, unpaired two-tailed Student’s test. ns, non-significant. (C) Detection of bZIP60 U and bZIP60 S in 3-week-old seedlings from the wild type, bzip60-1 and bzip60-2 mutants. Seedlings were treated with 2 mM DTT or 5 μg/mL Tm for 2 h in liquid MS medium (see S1 Text). 0.1% DMSO was used as a vehicle control. Actin II served as a loading control. The number represents the biological replicates. Note that both unspliced and spliced bZIP60 (bZIP60ΔC) were not detectable in the bzip60-2 mutant. bZIP60 U (bZIP60ΔN U for bzip60-1 mutant) could be detected in the wild type and bzip60-1 mutant regardless of stress treatment or not. Lower level of bZIP60 S (bZIP60ΔN S) was found in the bzip60-1 mutant under DTT or Tm treatment, compared to the wild type. (TIF) [file pgen.1005164.s009.tif]

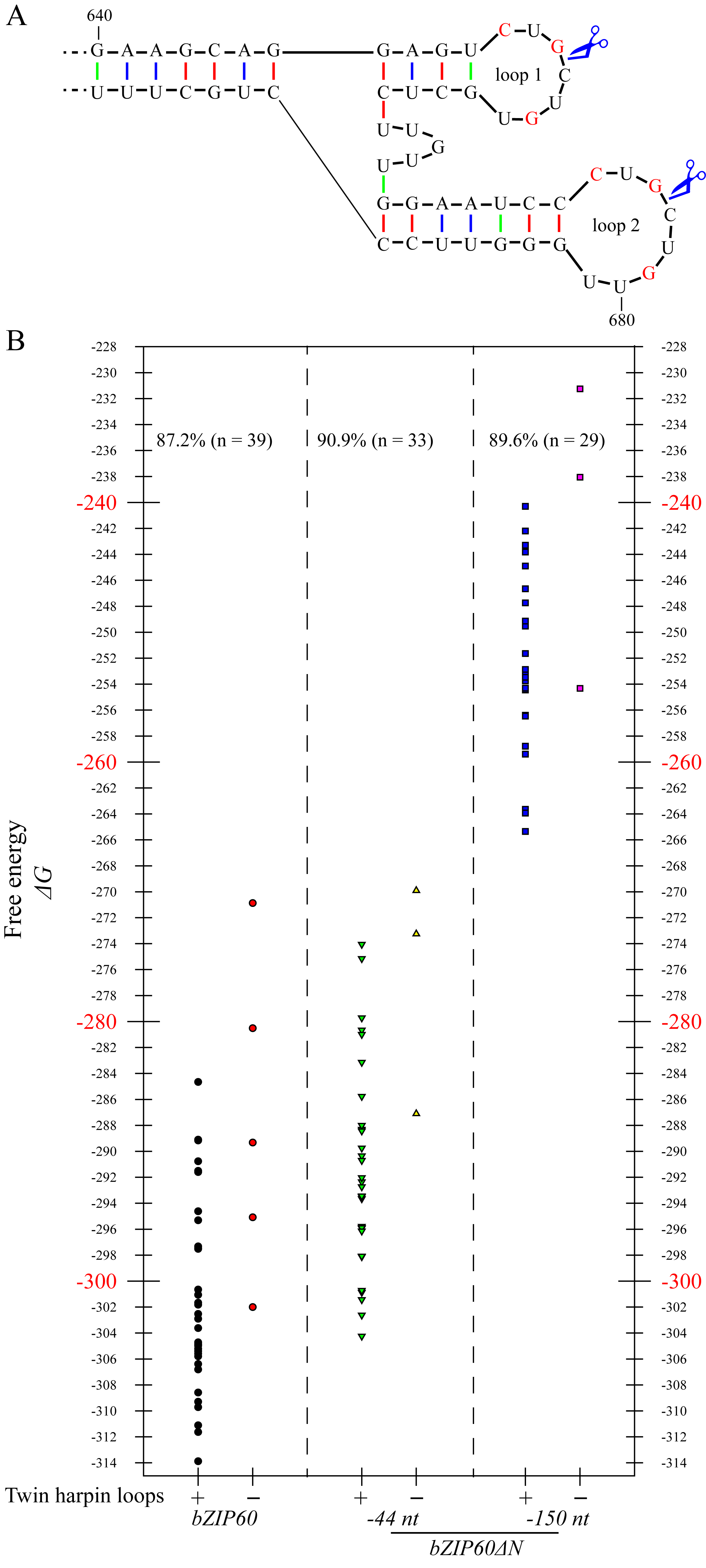

Supplement: S10 Fig — (A) Both bZIP60 and bZIP60ΔN folds into kissing hairpin loop containing two splicing sites indicated by scissors. Each loop contains three conserved amino acids (red). (B) The percentage of predicted different free energy forms of bZIP60 mRNAs with the twin kissing hairpin is compared between full length bZIP60, bZIP60ΔN without the first 44 nt and with a T-DNA sequence (TGTTATT) (-44 nt), and bZIP60ΔN without the first 150 nt (-150 nt). n indicates the total number of predicted bZIP60 mRNAs structure. (TIF) [file pgen.1005164.s010.tif]

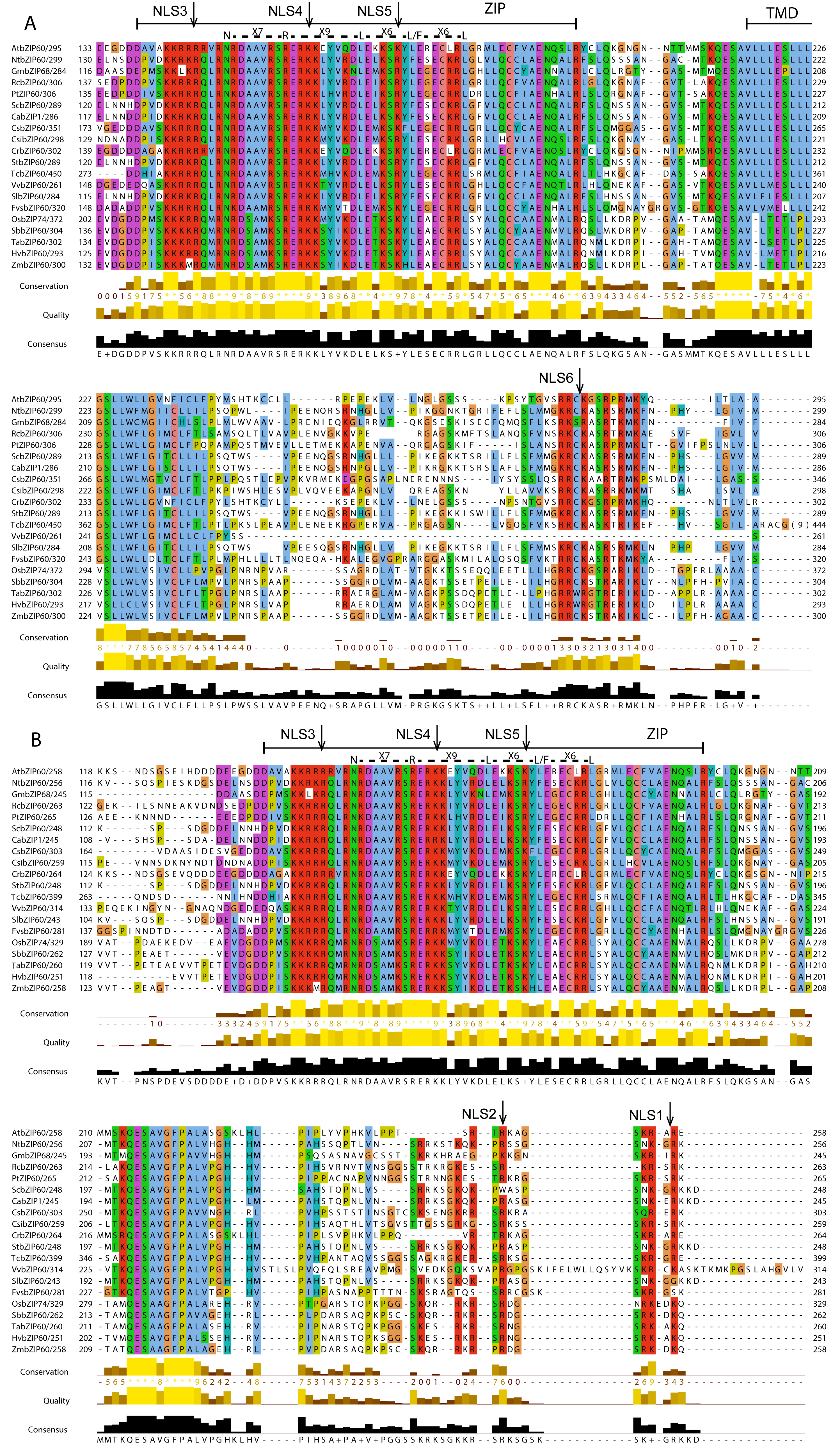

Supplement: S11 Fig — Two conserved regions of these proteins ware found on unspliced proteins (A), one is NLS/ZIP and the other TMD that is absent in spliced proteins (B) (see S5 Fig). A schematic of the bZIP consensus is shown above by highlighting the extremely conserved residues [54]. The NSL consensus motifs are indicated by down arrows. Note that the slicing produces two NLS motifs in the new sequences of spliced proteins (NLS1 and NLS2). Other information of these proteins is presented in detail in S2 Table. (TIF) [file pgen.1005164.s011.tif]

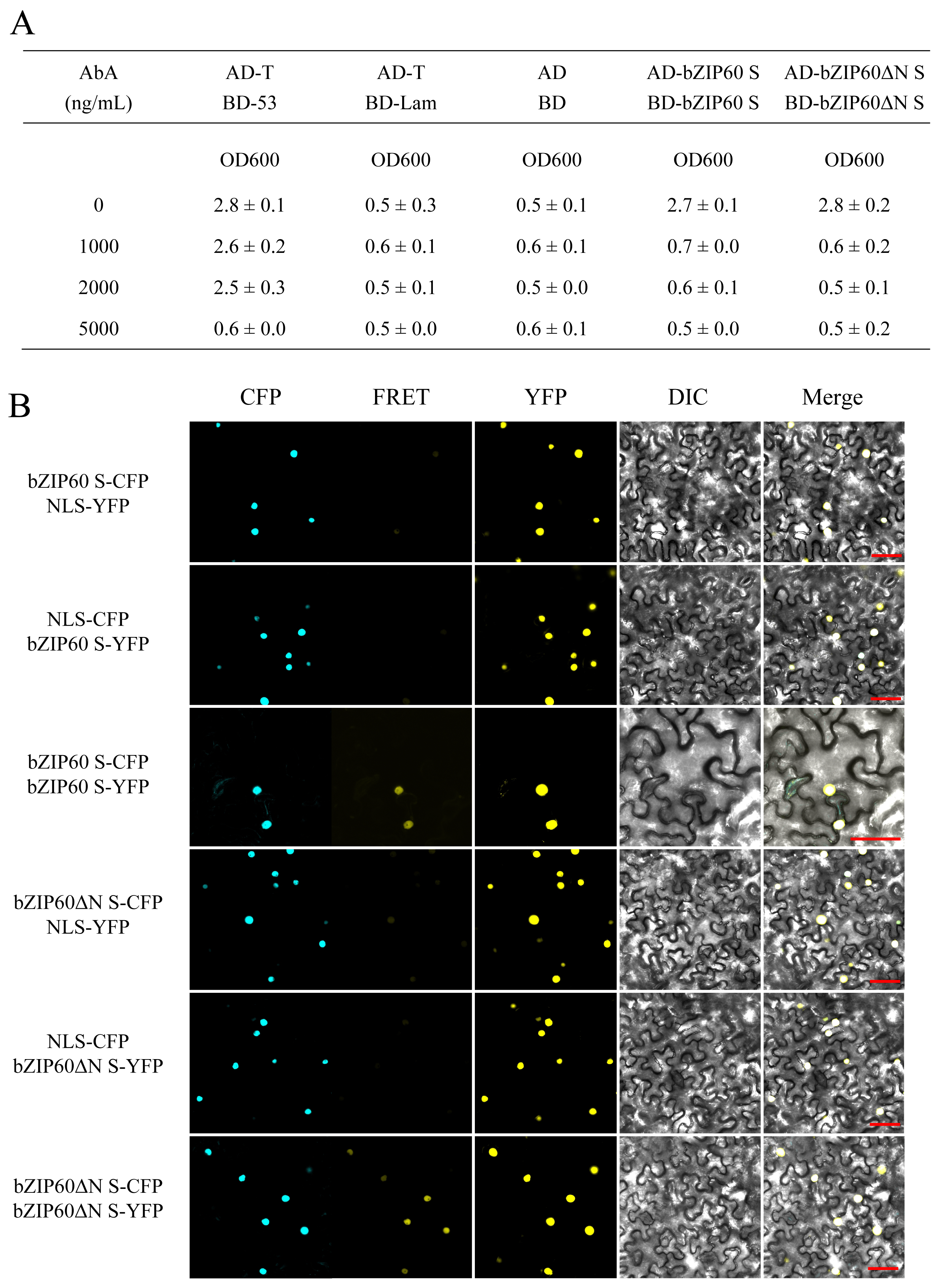

Supplement: S12 Fig — (A) The strength of homo-interactions of bZIP60 S and bZIP60ΔN S was tested by a liquid selective QDO medium containing AbA. The transformed cells were cultured in DDO medium overnight, pelleted, washed and diluted to an OD600 = 0.5 by a selective QDO medium with different concentrations of AbA. Data represent means with SD of three biological replicates. Note that the homo-interaction of bZIP60 S and bZIP60ΔN S could no longer support yeast growth in the presence of 1000 ng/mL AbA, compared to controls (also see Fig 6A). (B) Homo-interactions of bZIP60 S and bZIP60ΔN S in living cells by sensitized emission FRET assay. Note that only cells co-expressing bZIP60 S-CFP and bZIP60 S-YFP or bZIP60ΔN S-CFP and bZIP60ΔN S-YFP exhibited FRET signal. Experiments were repeated three times with similar results. Bars = 50 μm. (TIF) [file pgen.1005164.s012.tif]

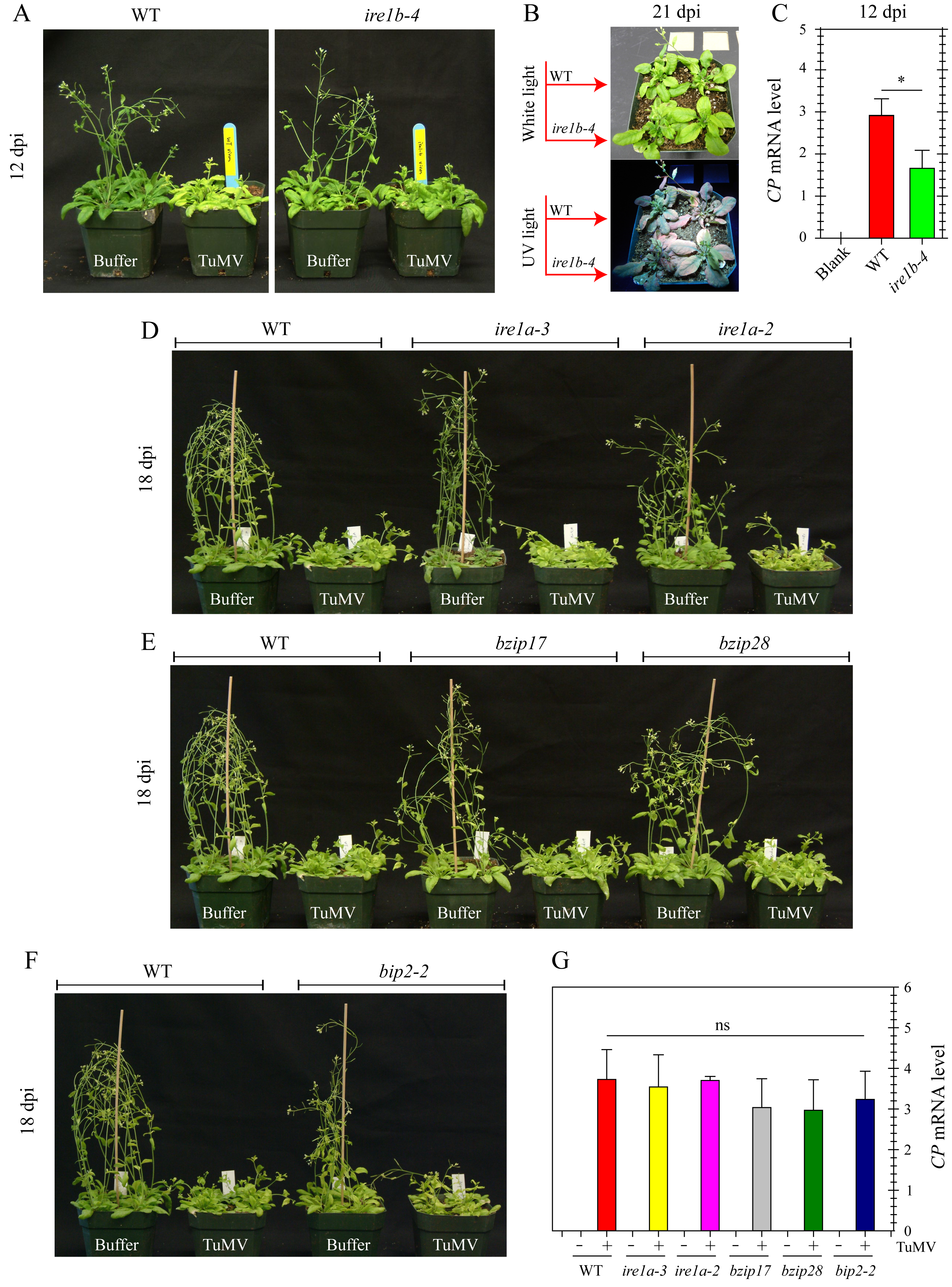

Supplement: S13 Fig — (A) Phenotypes of the wild type and ire1b-4 mutant at 12 dpi after inoculation with buffer or TuMV. Note that although ire1b-4 developed slower TuMV symptoms during a little early stage of virus infection (12 dpi), it eventually produced same viral symptoms at 21 dpi, observed in white light and ultraviolet (UV) lamp (B), compared to the wild type. (D), (E) and (F) Phenotypes of the wild type, two IRE1A mutants (ire1a-3 and ire1a-2), bzip17, bzip28 and bip2-2 at 18 dpi after inoculation with buffer or TuMV. Note that the mutant mutants all developed typical TuMV symptoms, compared to the wild type. (C) and (G) qRT-PCR analysis of TuMV CP in the wild type and the single mutants. At 12 dpi (for ire1b-4) (C) or 18 dpi (G) after inoculation with buffer or TuMV, RNA was extracted from the systemic leaves, and qRT-PCR was carried out. Actin II was used as an internal control for quantitative RT-PCR. Data represent means with SD of three biological replicates. * P<0.05, unpaired two-tailed Student’s test. ns, non-significant. (TIF) [file pgen.1005164.s013.tif]

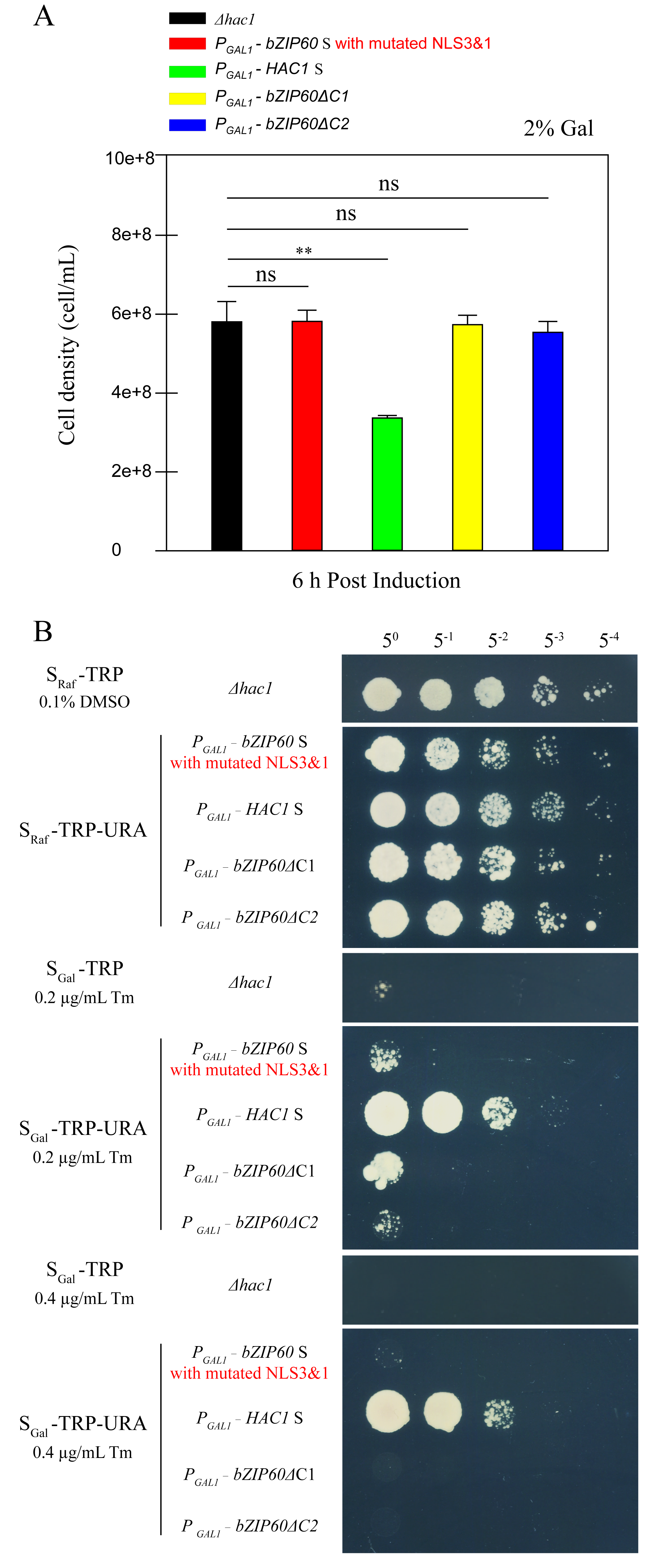

Supplement: S14 Fig — bZIP60 S with mutated NLS3 and NLS1 (see Figs 5 and 9), bZIP60ΔC1 and bZIP60ΔC2 (see S8 Fig) failed to inhibit yeast growth (A) and to rescue the ER-stress sensitive phenotype of CRY1 Δhac1::TRP strains (B), compared to the untransformed cells and the transformed cells with HAC1p S. The assays were conducted according to the procedures presented in detail in Figs 10 and 11. (A) Data represent means with SD of three experiments. *** P<0.001, unpaired two-tailed Student’s test. ns, non-significant. (B) Experiments were repeated three times with similar results. (TIF) [file pgen.1005164.s014.tif]

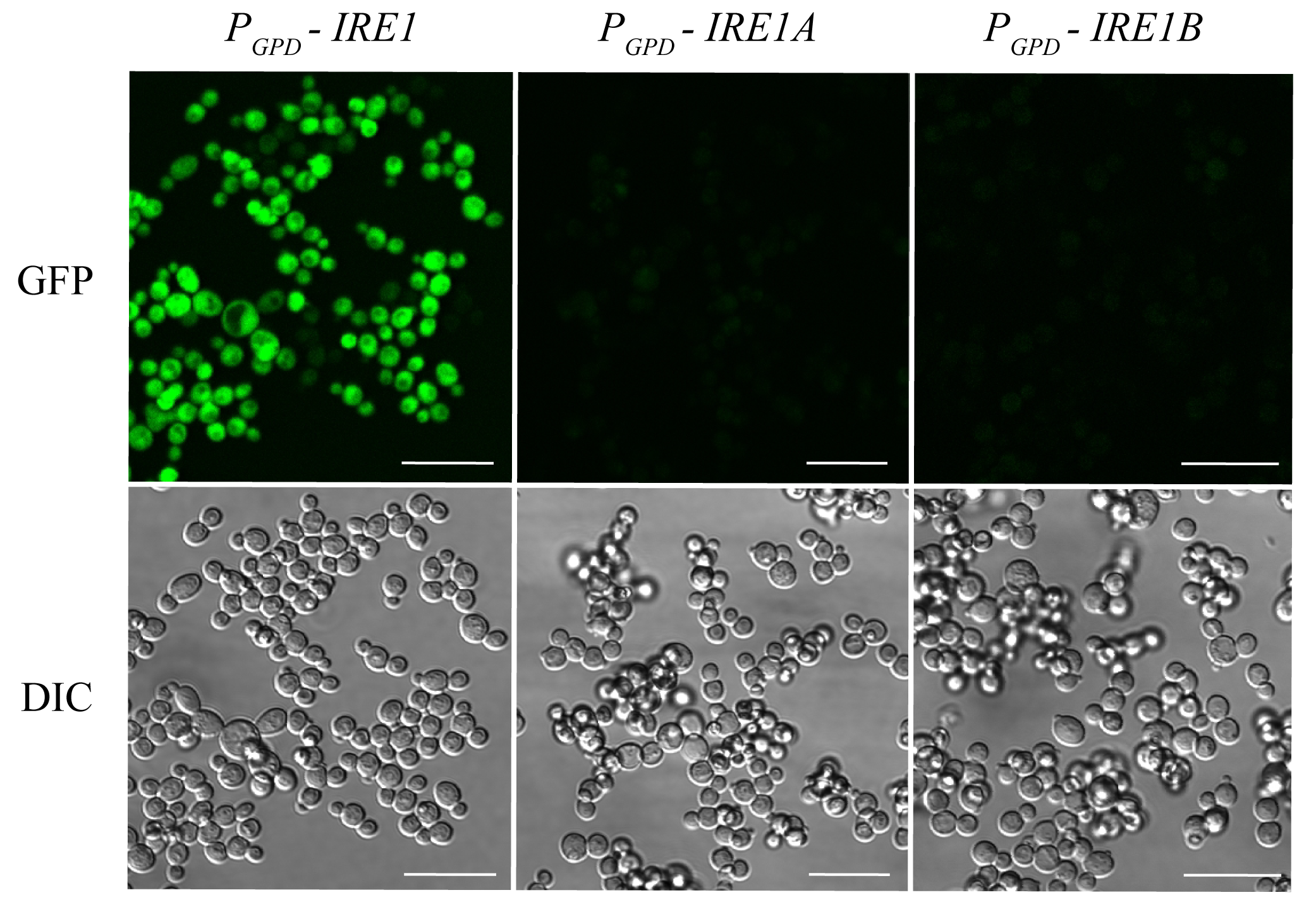

Supplement: S15 Fig — Functional complementation was tested by confocal imaging of CRY1 Δire1::KanMX6 strains with an integrated pRS304 4 x UPRE-GFP reporter. The cells were transformed with CEN-ARS plasmids expressing yeast IRE1, Arabidopsis IRE1A or IRE1B under the control of a GPD promoter. The transformed yeasts were selected and grown in the 2 x SD medium deficient in TRP and URA with 250 μg/mL G418 (10131–035, Invitrogen) and 2% raffinose, using monosodium glutamate (G1626, Sigma) as nitrogen source. ER stress was induced by 2 mM DTT in the presence of 2% glucose. Experiments were repeated three times with similar results. Bars = 20 μm. (TIF) [file pgen.1005164.s015.tif]

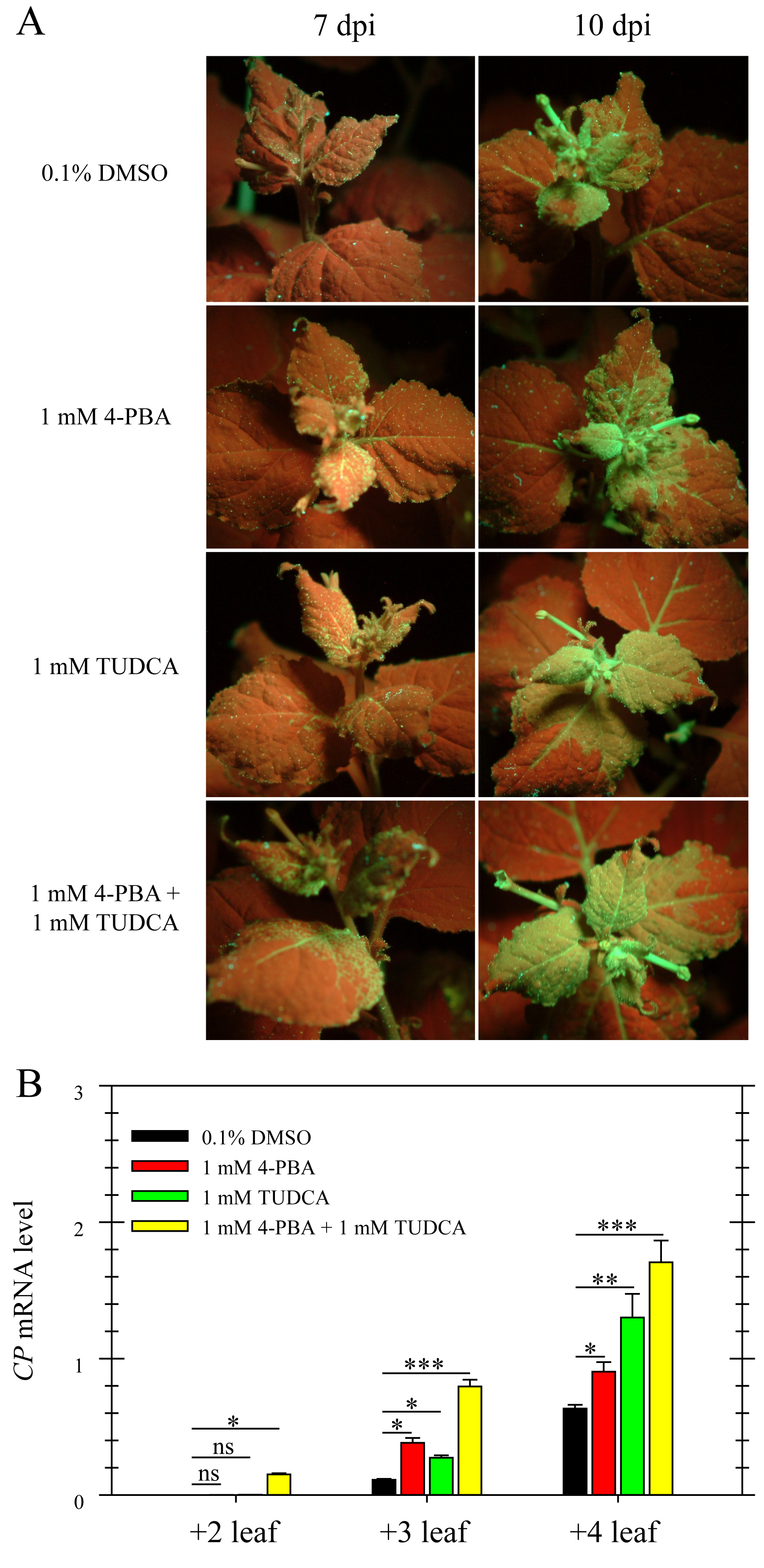

Supplement: S16 Fig — N. benthamiana seedlings were pre-treated with 0.1% DMSO, 4-PBA (1 mM), TUDCA (1 mM) or 4-PBA (1 mM) plus TUDCA (1 mM) for 6 h (see S1 Text). The pre-treated leaves were then selected to be inoculated with TuMV. After 7 and 10 dpi, pictures of N. benthamiana seedlings were taken under UV light (A), and RNA was extracted from the indicated leaves. (B) TuMV accumulation indicated by CP mRNA level was analyzed by qRT-PCR. Data represent means with SD of three experiments. *P<0.05, **P<0.01, ***P<0.001, unpaired two-tailed Student’s test, ns, non-significant. (TIF) [file pgen.1005164.s016.tif]

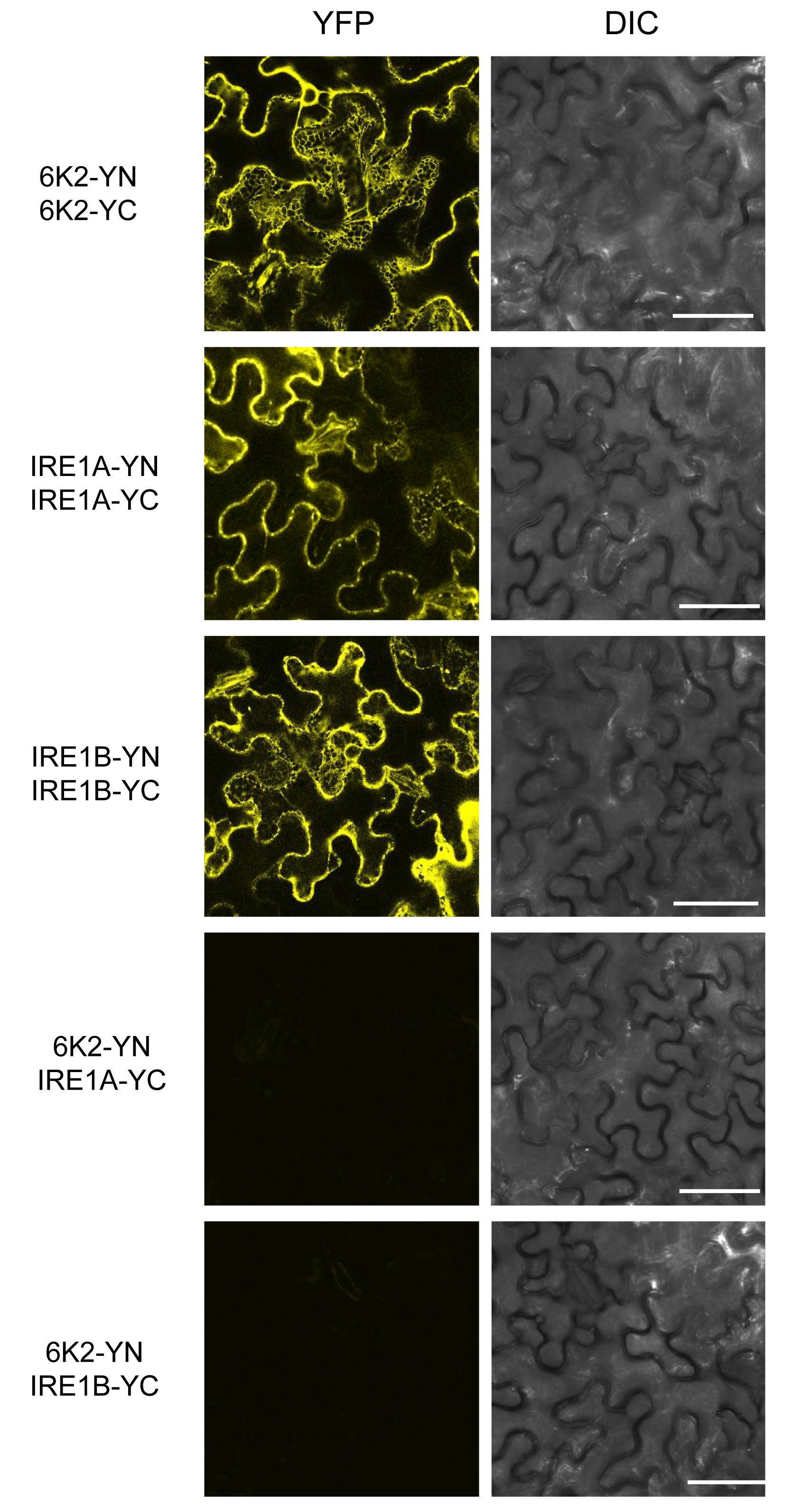

Supplement: S17 Fig — The relevant entry vectors were cloned into the Gateway version of bimolecular fluorescence complementation (BiFC) vectors to fuse the split YN and YC at the C-termini of 6K2, IRE1A and IRE1B. The sets of constructs were subjected to the transient expression system, and YFP signal was captured under the confocal at excitation 514 nm and emission 525–600 nm. BiFC assay demonstrated the homo-interactions of TuMV 6K2, IRE1A and IRE1B, but no interaction of TuMV 6K2 with IRE1A or IRE1B. Experiments were repeated three times with similar results. Bars = 50 μm. (TIF) [file pgen.1005164.s017.tif]

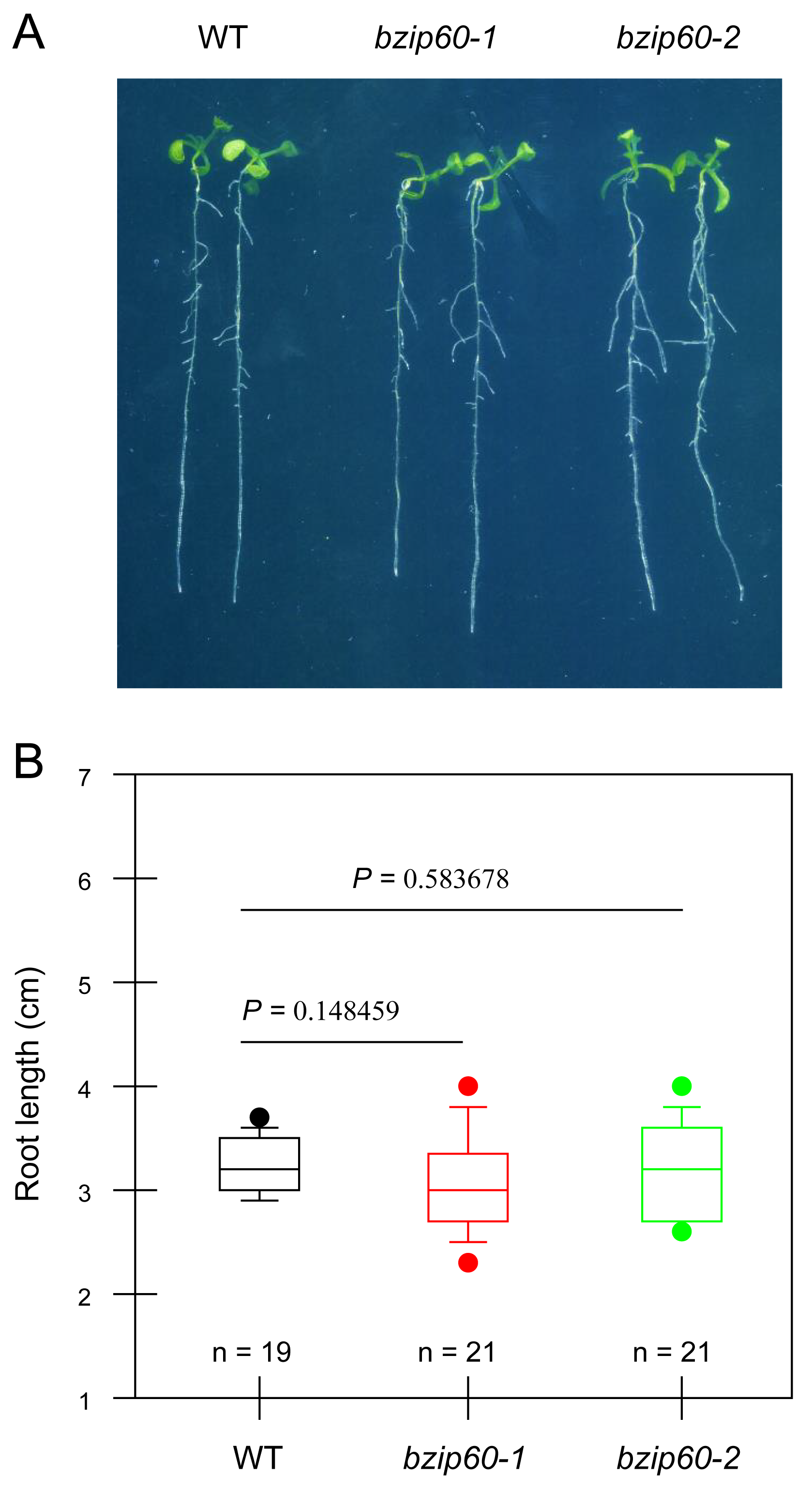

Supplement: S18 Fig — (A) The wild type, bzip60-1 and bzip60-2 mutants were grown on half-strength MS medium for 9 d after germination. (B) Root lengths were measured in 9-d-old seedlings. Box plots represent the value range and the variability of root lengths. The boundaries of each box represent the lower 25th and upper 75th percentiles, and the horizontal line within the box represents the median value. The spacing within the box indicates the degree of dispersal in the data. The lines at the top and bottom of the box (whiskers) represent the minimum and maximum. Outliers are indicated by solid circles. Statistical analysis was conducted and showed no difference in root length among wild type and mutants. (TIF) [file pgen.1005164.s018.tif]
