# Supplementary material for: The UPR Branch IRE1-bZIP60 in Plants Plays an Essential Role in Viral Infection and Is Complementary to the Only UPR Pathway in Yeast
Source: PLoS Genet. 2015 Apr 15;11(4):e1005164. doi: 10.1371/journal.pgen.1005164 (PMC4398384; doi:10.1371/journal.pgen.1005164)
Supplement: S1 Table — (DOC) [file pgen.1005164.s019.doc]

**S1 Table.** **Primers Used in this Study.**

| **Name** | **Sequences (5’-3’)** | **Purpose** | **Reference/Note** |
| --- | --- | --- | --- |
| LBb1.3 | ATTTTGCCGATTTCGGAAC | Genotyping analysis of *Arabidopsis* | Primers were designed by the T-DNA Primer Design (<http://signal.salk.edu/tdnaprimers.2.html>)  Note:  LBb1.3 for SALK lines  LB2 for SAIL lines  WiscDsLox LB for WiscDsLox lines |
| LB2 | GCTTCCTATTATATCTTCCCAAATTACCAATACA |
| WiscDsLox LB | AACGTCCGCAATGTGTTATTAAGTTG |
| bzip60-1 LP | GGAAGAAAAGTCCTCTCGGAG |
| bzip60-1 RP | CACAGCATCATCGTCTCCTTC |
| bzip60-2 LP | CAAAGATTGGCTCGTCTGAAC |
| bzip60-2 RP | TGCTTGCGTATCTTGTGATATG |
| bip2 LP | CGACAGGTGCGACTAAAAATC |
| bip2 RP | AGGTCACATTTGAAGTGGACG |
| ire1a-2 LP | TGCGTTCAGACACTAACATGC |
| ire1a-2 RP | GAAGAAGAACCGTAAATCCGC |
| ire1a-3 LP | CAAAATCTTCAGTGCTAGCGG |
| ire1a-3 RP | TATCTCCGATCCATCGTTGAC |
| ire1b-4 LP | CCTCTCGAACCCTTCAGGTAC |
| ire1b-4 RP | GAAGGAAAACGGACATCCTTC |
| bzip28 LP | TTTATCATCATTTTGGTCGCC |
| bzip28 RP | TATCCCCTAACAGGATACGGC |
| bzip17 LP | TTGTCATTCCTCGGAACAAAG |
| bzip17 RP | GGACGATCTTTACTTCCCTGC |
| dP1 F | ggggacaagtttgtacaaaaaagcaggcttcATGGCAGCAGTTACATTCGCATC | TuMV single factor cloning into the pDONR221 entry vector | The TuMV genome sequence used in this study is retrieved from GenBank (<http://www.ncbi.nlm.nih.gov/Genbank/>) with accession number EF028235  Note:  The sequences in lowercase represent the *aat*B Gateway primers |
| dP1 R | ggggaccactttgtacaagaaagctgggtcAAAGTGCACAATCTTGTGACTCA |
| dHC-Pro F | ggggacaagtttgtacaaaaaagcaggcttcATGGGAGCCAACTTCTGGAAAGGC |
| dHC-Pro R | ggggaccactttgtacaagaaagctgggtcTCCAACGCGGTAGTGTTTCAAG |
| dP3 F | ggggacaagtttgtacaaaaaagcaggcttcATGGGAACAGAATGGGAGGACACTCA |
| dP3 R | ggggaccactttgtacaagaaagctgggtcTTGATGAACCACCGCCTTTTCTT |
| d6K1 F | ggggacaagtttgtacaaaaaagcaggcttcATGGCGAAGAGACAATCCGAGCAAGA |
| d6K1 R | ggggaccactttgtacaagaaagctgggtcCTGATGGTAGACTGTAGGTTCCA |
| dCI F | ggggacaagtttgtacaaaaaagcaggcttcATGACTCTCAATGATATAGAGGATGA |
| dCI R | ggggaccactttgtacaagaaagctgggtcTTGATGGTGAACTGCCTCAAGAG |
| d6K2 F | ggggacaagtttgtacaaaaaagcaggcttcATGAACACCAGCGACATGAGCAAATT |
| d6K2 R | ggggaccactttgtacaagaaagctgggtcTTCATGGGTTACGGGTTCGGACA |
| dNIaVPg F | ggggacaagtttgtacaaaaaagcaggcttcATGGCGAAAGGTAAGAGGCAAAGG |
| dNIaVPg R | ggggaccactttgtacaagaaagctgggtcCTCGTGGTCCACTGGGACGAGTT |
| dNIaPro F | ggggacaagtttgtacaaaaaagcaggcttcATGAGTAACTCCATGTTCAGAGGGTT |
| dNIaPro R | ggggaccactttgtacaagaaagctgggtcTTGTGCGTAGACTGCCGTGCTGT |
| dNIb F | ggggacaagtttgtacaaaaaagcaggcttcATGACCCAGCAGAATCGGTGGATGTT |
| dNIb R | ggggaccactttgtacaagaaagctgggtcCTGGTGATAAACACAAGCCTCAG |
| dCP F | ggggacaagtttgtacaaaaaagcaggcttcATGGCAGGTGAAACGCTTGATGCAGG |
| dCP R | ggggaccactttgtacaagaaagctgggtcCAACCCCTGAACGCCCAGTAAGT |
| dHAC1 F | ggggacaagtttgtacaaaaaagcaggcttcATGGAAATGACTGATTTTGAAC | Cloning yeast *IRE1*, *HAC1* S and *HAC*1 U into the pDONR221 entry vector | Note:  The sequences in lowercase represent the *aat*B Gateway primers |
| dHAC1 R | ggggaccactttgtacaagaaagctgggtcTGAAGTGATGAAGAAATCATTC |
| dIRE1p F | ggggacaagtttgtacaaaaaagcaggcttcATGCGTCTACTTCGAAGAAACATGT |
| dIRE1p R | ggggaccactttgtacaagaaagctgggtcTGAATACAAAAATTCACGTAAAATTTG |
| dbZIP60 F | ggggacaagtttgtacaaaaaagcaggcttcATGGCGGAGGAATTTGGAAGCAT | Cloning *Arabidopsis* *IRE1A*, *bZIP60* U, *bZIP60* S (both *bZIP60* and *bZIP60ΔN*) as well as *bZIP60ΔCs* into the pDONR221 entry vector |
| dbZIP60 R | ggggaccactttgtacaagaaagctgggtcCGCCGCAAGGGTTAAGATTTGGT |
| dbZIP60ΔN F | ggggacaagtttgtacaaaaaagcaggcttcATGAACGATGAGAATCATCAAGAGG |
| bZIP60ΔC1 R | gggaccactttgtacaagaaagctgggtcTTGCAAACAGTAACGTAGAG |
| bZIP60ΔC2 R | gggaccactttgtacaagaaagctgggtcGTACATTAAAAACGTCCG |
| dIRE1A F | ggggacaagtttgtacaaaaaagcaggcttcATGCCGCCGAGATGTCCTTTCC |
| dIRE1A R | ggggaccactttgtacaagaaagctgggtcGATGATGTCGCATTTGAAGTACT |
| IRE1B-KpnI F | GGTACCGAATGAGAGGATCTGCACTACTTGA | Cloning *Arabidopsis* *IRE1B* into the pENTR 1A Dual Selection entry vector | Note:  The underlined nucleotides indicate KpnI and EcoRI sites. |
| IRE1B-EcoRI R | GAATTCCGGAATACAGTGGTCTTAGAGTACTTG |
| NLS1 F | CCACACAAAGTGTTGCCTCCTACGTCCAGAACCAGAAAAGCTGGTTCTGCAGCTGCTGCTGAGTAGTAGCAAACCGTCTTATACCGGC | Mutating the putative NLSs occurred in bZIP60 S and bZIP60ΔN S in the pDONR221 entry vector |  |
| NLS1 R | GCCGGTATAAGACGGTTTGCTACTACTCAGCAGCAGCTGCAGAACCAGCTTTTCTGGTTCTGGACGTAGGAGGCAACACTTTGTGTGG |
| NLS2 F | CCACACAAAGTGTTGCCTCCTACGTCCGCAACCGCAGCAGCTGGTTCTAAACGGGCTCGGGAGTAGTAGCAAACCGTCTTATACCGGC |
| NLS2 R | GCCGGTATAAGACGGTTTGCTACTACTCCCGAGCCCGTTTAGAACCAGCTGCTGCGGTTGCGGACGTAGGAGGCAACACTTTGTGTGG |
| NLS1&2 F | CCACACAAAGTGTTGCCTCCTACGTCCGCAACCGCAGCAGCTGGTTCTGCAGCAGCTGCAGAGTAGTAGCAAACCGTCTTATACCGGC |
| NLS1&2 R | GCCGGTATAAGACGGTTTGCTACTACTCTGCAGCTGCTGCAGAACCAGCTGCTGCGGTTGCGGACGTAGGAGGCAACACTTTGTGTGG |
| NLS3 F | GACGAAGAAGGAGACGATGATGCTGTGGCTGCTGCTGCTGCTGCTGCTGTAAGAAATAGAGATGCGGCGGTTAGA |
| NLS3 R | TCTAACCGCCGCATCTCTATTTCTTACAGCAGCAGCAGCAGCAGCAGCCACAGCATCATCGTCTCCTTCTTCGTC |
| NLS3Δ F | GATGATGACGAAGAAGGAGACGATGATGCTGTGGCTGTAAGAAATAGAGATGCGGCGGTTAGATCGAGAGAGA |
| NLS3Δ R | TCTCTCTCGATCTAACCGCCGCATCTCTATTTCTTACAGCCACAGCATCATCGTCTCCTTCTTCGTCATCATC |
| NLS4&5 F | GCGGTTAGATCGGCGGAGGCGGCGGCGGAATATGTACAAGATTTAGAGGCGGCGAGTGCGTATCTCGAAAGAGAATGCTTGAGACT |
| NLS4&5 R | AGTCTCAAGCATTCTCTTTCGAGATACGCACTCGCCGCCTCTAAATCTTGTACATATTCCGCCGCCGCCTCCGCCGATCTAACCGC |
| NLS(atg)-Creating F | ggggacaagtttgtacaaaaaagcaggcttcATGCAGCCTAAGAAGAAGAGAAAGGTTGGAGGA | Creating 35S::NLS-CYF reporter |  |
| NLS(atg)-Creating R | ggggaccactttgtacaagaaagctgggtcTCCTCCAACCTTTCTCTTCTTCTTAGGCTGCAT |
| bZIP60HindIII-F | CCCAAGCTTttttacaatggaactcacctgaaaataacac | Cloning bZIP60 promoter into the pMDC43 | Note:  The underlined nucleotides indicate HindIII and KpnI sites. |
| bZIP60KpnI-R | CGGGGTACCggtcaaaaaaaaaaaaatatacaaagaagaaaaaaaaaag |
| NF | ATGGCGGAGGAATTTGGAAGCA | Semi-quantitative RT-PCR for analysing *bZIP60* transcript fragments in the two *bzip60* mutants |  |
| NR | ATCCGGTGAAGACTGAAGAAAATC |
| MF | ACTAGCGATTCTGGCTCCGTTG |
| MR | GCAAACAGTAACGTAGAGACTGG |
| CF | CCCTTATATGTCCCACACAAAG |
| CR | GGCCTCGAACCCTTACATCTC |
| qbZIP60 F | CGATGATGCTGTGGCTAAAA | Quantitative RT-PCR for detecting *bZIP60* transcript fragments in the two *bzip*60 mutants | Reference 52 |
| qbZIP60 R | TCTCAAGCATTCTCTTTCGAGAT |
| bZIP60­-1218 R | ACTCCCAGAAGCCAAAGC | For 5′ RACE and analysis of T-DNA insertion into *bZIP60* genomic DNA | AAP is provided in 5′ RACE System Kit (Invitrogen, USA), in which I represents deoxyinosine |
| bZIP60-GSP R | GAACCCTTACATCTCCGACTAAC |
| AAP | GGCCACGCGTCGACTAGTACGGGIIGGGIIGGGIIG |
| bZIP60 S7 | ATTCTGGTAGCGAGATTCATGATGATG |
| bZIP60 S F | GAAGGAGACGATGATGCTGTGGCT | Semi-quantitative and quantitative (q) RT-PCR for detecting *bZIP60* S and *bZIP60* U in *Arabidopsis* | Reference 15 |
| qbZIP60 S F | CTAGGACGTATGCTTGAGTGCTTCGTT |
| qbZIP60 S R | AGCAGGGAACCCAACAGCAGACT |
| qbZIP60 U R | GCAGGGATTCCAACAAGAGCACAG |
| bZIP60 AIw F | GAAGGAGACGATGATGCTGTGGCT | Semi-quantitative RT-PCR for flanking assay in *Arabidopsis* | Reference 30 |
| bZIP60 AIw R | GAACCCTTACATCTCCGACTAAC |
| qCP F | AATCCTATACACGCCGGAGCAGAC | Semi-quantitative and quantitative (q) RT-PCR for detecting TuMV *CP* transcripts |  |
| qCP R | CTCCGTCAGTTCGTAATCAGC |
| CP F | GGCACTCAAGAAAGGCAAGG |
| CP R | CTCCGTCAGTTCGTAATCAGC |
| qBiP3 F | CACGGTTCCAGCGTATTTCAAT | Semi-quantitative and quantitative (q) RT-PCR for detecting the UPR mark expression in *Arabidopsis* | Reference 37 |
| qBiP3 R | ATAAGCTATGGCAGCACCCGTT |
| qPDI F | CTCGTGAAGCTGAGGGTATTG |
| qPDI R | TGTGCGAAATCTAACTCAGAG |
| qBiP1/2 F | TCACTTGGGAGGTGAGGACTTT |
| qBiP1/2 R | CTCACATTCCCTTCGGAGCTTA |
| qCRT F | AGACCTTAGTCTTCCAATTCTC |
| qCRT R | CCATTGTAAGTAAGGATAGCATG |
| Actin II F | CGAGGCTCCTCTTAACCCAAAGG | Semi-quantitative and quantitative RT-PCR for the internal control *Actin II* in *Arabidopsis* |  |
| Actin II R | GACACACCATCACCAGAATCCAGC |
| 18S RNA F | ATGGCCGTTCTTAGTTGGTGGAGC | Quantitative RT-PCR used in *N. benthamiana* | Reference 75 |
| 18S RNA R | AGTTAGCAGGCTGAGGTCTCGAAC |
| qNtbZIP60 S F | GGGGTTAGTTCTCCAGTGTTGTC |
| qNtbZIP60 S R | AGGGAACCCAACAGCAGACT |
