# Supplementary material for: The UPR Branch IRE1-bZIP60 in Plants Plays an Essential Role in Viral Infection and Is Complementary to the Only UPR Pathway in Yeast
Source: PLoS Genet. 2015 Apr 15;11(4):e1005164. doi: 10.1371/journal.pgen.1005164 (PMC4398384; doi:10.1371/journal.pgen.1005164)
Supplement: S2 Table — (DOC) [file pgen.1005164.s020.doc]

**S2 Table. *Arabidopsis bZIP60* Homologues Analyzed in this Study.**

| **Gene** | **Gene ID** | **Protein ID** | **Species** | **Organism** |
| --- | --- | --- | --- | --- |
| AtbZIP60 | AT1G42990 | AAK76638 | Arabidopsis | *Arabidopsis thaliana* |
| NtbZIP60 | AB281271 | BAF76429 | Tobacco | *Nicotiana tabacum* |
| GmbZIP68 | NM_001250115.2 | NP_001237044 | Soybean | *Glycine max* |
| RcbZIP60 | XM_002510694 | XP_002510740.1 | Caotor | *Ricinus communis* |
| PtZIP60 | XM_002307788 | XP_002307824 | Poplar tree | *Populus trichocarpa* |
| ScbZIP60 | EU526016 | ACB32232 | Chaco potato | *Solanum chacoense* |
| CabZIP1 | AY775332 | AAX20030 | Pepper | *Capsicum annuum* |
| CsbZIP60 | XM_004157015 | XP_004157063 | Cucumber | *Cucumis sativus* |
| CsibZIP60 | XM_006473806 | XP_006473869 | Sweet orange | *Citrus sinensis* |
| CrbZIP60 | XM_006305391 | XP_006305453 | Capsella | *Capsella rubella* |
| StbZIP60 | XM_006342130 | XP_006342192 | Potato | *Solanum tuberosum* |
| TcbZIP60 | XM_007018577 | XP_007018639 | Cacao | *Theobroma cacao* |
| VvbZIP60 | XM_003634288 | XP_003634336 | Wine grape | *Vitis vinifera* |
| SlbZIP60 | XM_004238421 | XP_004238469 | Tomato | *Solanum lycopersicum* |
| FvsbZIP60 | XM_004300038 | XP_004300086 | Vesca | *Fragaria vesca subsp. vesca* |
| OsbZIP74 | LOC_Os06g41770 | LOC_Os06g41770 | Rice | *Oryza sativa* |
| SbbZIP60 | XM_002437252 | XP_002437297 | Sorghum | *Sorghum bicolor* |
| TabZIP60 | AK330972 | AK330972 | Wheat | *Triticum aestivum* |
| HvbZIP60 | AK365505 | BAJ96708 | Barley | *Hordeum vulgare subsp. vulgare* |
| ZmbZIP60 | BT086464 | ACR36817 | Maize | *Zea mays* |
