# Supplementary material for: The UPR Branch IRE1-bZIP60 in Plants Plays an Essential Role in Viral Infection and Is Complementary to the Only UPR Pathway in Yeast
Source: PLoS Genet. 2015 Apr 15;11(4):e1005164. doi: 10.1371/journal.pgen.1005164 (PMC4398384; doi:10.1371/journal.pgen.1005164)
Supplement: S1 Text — (DOC) [file pgen.1005164.s021.doc]

**S1 Text: Supporting Methods.**

**Stress Treatment by Tm and DTT**

To determine whether the truncated *bZIP60* mRNA in the *bzip60-1* allele is spliced in response to ER stress agents, 3-week-old seedlings grown on half-strength MS agar medium with 3% (w/v) sucrose were treated with liquid MS medium containing 2 mM DTT, 5 µg/mL Tm (T7765, Sigma) or 0.1% DMSO for 2 h. Then, whole plant from the indicated genotypes was subjected to RNA extraction.

**5**′ **Rapid Amplification of cDNA Ends (5**′ **RACE)**

For 5′ RACE, total RNA was extracted with TRIzol reagent (Invitrogen, USA) from 3-week-old *bzip60-1* mutant seeding treated with 0.1% DMSO or 5 µg/mL Tm or from the systemic leaves of 6-week-old *bzip60-1* mutant plants infected with TuMV. A 5 µg of total RNA was treated with DNase I (Invitrogen, USA) according to the manual, and the first strand cDNA was obtained by a SuperScript III First-Strand Synthesis System (Invitrogen, USA) using a gene specific primer (bZIP60-GSP R) 5’-GAACCCTTACATCTCCGACTAAC-3′. The converted cDNA was tailed in a TdT-tailing reaction as described in the 5′ RACE System Kit (Invitrogen, USA). A nested PCR of the dC-tailed cDNA was then done with KOD Xtreme Hot Start DNA Polymerase (71975, EMD Millipore), using bZIP60-1218 R primer 5′-ACTCCCAGAAGCCAAAGC-3′ and Abridged Anchor Primer kit-specific oligo (AAP) 5′-GGCCACGCGTCGACTAGTACGGGIIGGGIIGGGIIG-3′ (where I represents deoxyinosine). The major band from nested amplification was cloned using the pGEM-T Easy Vector System (Promega, USA). At least 4 clones from each condition were sequenced.

**Pharmacological Molecular Chaperones Treatment**

To determine the role of UPR in viral infection, pharmacological molecular chaperones were used to introduce an artificial UPR in *N. benthamiana.* One leaf of 7-week-old *N. benthamiana* seedlings was pre-treated with 0.1% DMSO, 1 mM 4-Phenylbutyric acid (P21005, SIGMA; 4-PBA), 1 mM tauroursodeoxycholic acid (580549, EMD chemicals; TUDCA) or 1 mM 4-PBA plus 1 mM TUDCA for 6 h. The pre-treated leaves were then rubbed with the recombinant TuMV-GFP virus. After 7 dpi and 10 dpi, *N. benthamiana* seedlings were photographed under a handheld UV lamp. After 9 dpi, the second leaf above the inoculated one (+2), the third leaf above the inoculated one (+3), and the fourth leaf above the inoculated one (+4 leaf) from *N. benthamiana* seedlings were collected for RNA extraction. Each treatment contains at least 5 plants. The experiment was performed three times.
